# Phosphatidylcholine could protect the defect of zearalenone exposure on follicular development and oocyte maturation

**DOI:** 10.18632/aging.101660

**Published:** 2018-11-25

**Authors:** Fang-Nong Lai, Xue-Lian Liu, Na Li, Rui-Qian Zhang, Yong Zhao, Yan-Zhong Feng, Charles Martin Nyachoti, Wei Shen, Lan Li

**Affiliations:** 1 College of Life Sciences, Institute of Reproductive Sciences, Qingdao Agricultural University, Qingdao 266109, China; 2 Institute of Animal Sciences, Heilongjiang Academy of Agricultural Sciences, HarbinHeilongjiang 150086, China; 3 Department of Animal Science, University of Manitoba, Winnipeg, MB R3T 2N2, Canada; * Equal contribution

**Keywords:** zearalenone, follicle, oocyte, metabolomics, lysophosphatidylcholine

## Abstract

Zearalenone (ZEA) is a well-known exogenous endocrine disruptor and can lead to severe negative effects on the human and animal reproductive process. Using a follicle culture model, we have previously shown that ZEA exposure significantly affected the follicular development and antrum formation but the underlying mechanisms are not well known. Therefore, in this study, we explored the metabolomic changes of granulosa cell (GC) culture media with or without ZEA exposure. The results showed that ZEA significantly increased phosphatidylcholine or phosphatidyl ethanolamine adducts in culture medium. A comprehensive analysis with the metabolome data from follicular fluid of small and large antral follicles showed that lyso phosphatidylcholine (LPC) was accumulated during follicle growth, but was depleted by ZEA exposure. Exogenous supplement with LPC to the follicle growth media or oocyte maturation media can partly protect the defect of ZEA exposure on follicular antrum formation and oocyte maturation. Taken together, our results demonstrate that ZEA exposure hinders the follicular growth and exogenous LPC can practically protect the defect of ZEA on follicular development and oocyte maturation.

## Introduction

Mycotoxin contamination affects human food and livestock feeding diet worldwide. Among the various mycotoxins, zearalenone (ZEA), a nonsteroidal environmental endocrine disruptor, is produced by *Fusarium* fungi. *Fusarium* fungi are known to contaminate cereals such as maize, wheat, and rice, particularly under high moisture conditions. ZEA has been shown to elicit various deleterious effects on the reproductive organs of human and animals [1–15]. ZEA was detected in urine in 78.5% of a group of 163 New Jersey girls aged 9 - 10 and was associated with the early onset of breast development [1]. In a cohort study ZEA exposure led to precocious puberty and was correlated with height and weight of young girls who lived in the Tuscany area [2]. For domestic animals, the pig is the most sensitive species to the adverse effects of ZEA which induced infertility, reproductive disorders, decreased fetal viability and subsequently reduced litter size [3,9,13,14]. In mice and rats, gestational exposure to ZEA caused early fetal death and delay in fetal development and ZEA also impacted oocyte quality and follicular development, such as inhibition of oocyte maturation with abnormal spindle morphologies, disruption of actin filaments, and disturbing cortical granule extrusion [4,5,11,15]. In addition, ZEA altered DNA methylation and histone methylation and H3K4me2 and H3K9me3 was decreased in the oocyte [12]. Furthermore, ZEA interfered the steroid production of ovarian granulosa cells (GCs) via disturbing follicle stimulating hormone (FSH) - or IGF - induced progesterone production and the transcription of p450scc and 3β-hydroxysteroid dehydrogenase (HSD) [9,13]. Thus, ZEA widely affects animal and human reproductive functions.

Folliculogenesis is an ordered sequence of oocyte development and maturation which also involves the proliferation and differentiation of granulosa cells, and is crucial for mammalian reproduction [16]. During follicular development, a series of changes have been occurred. First, the primordial follicle grows into a growth follicle with somatic cells proliferation. After that, the follicular antrum is formed, with the ovarian granulosa cell proliferation and differentiation. Then, the majority of follicles become atresia and only few grow dramatically to become dominant follicle and ovulation. Follicular fluid (FF) within follicular antrum is the microenvironment of oocyte, containing hormones, growth factors, proteins and phospholipids which are partially produced by GCs. Phospholipid is the most abundant lipid in the cell membrane, including Lysophosphatidic Acid (LPA), LysophosphatidylCholine (LPC), Sphingosine-1 Phosphates and Sphingophoryl Choline [17]. Some studies have shown that LPC is metabolized into LPA by phospholipase [18,19]. In mammals, there are at least five different high affinity LPA receptors which couple the different transmembrane G protein coupled receptors to activate different signal pathways to play different biological functions, such as cell proliferation, cell survival, cell differentiation, cell gap linking and cell morphological changes [17]. LPA has been detected in many biological fluids, such as blood, plasma, tears, ascites, seminal plasma and FF [20]. However, the composition changes of FF during follicular development as well as the effects of phosphatidylcholine on metabolic of GCs are still poor understood.

Metabolomics is defined as the quantitative evaluation of endogenous metabolites in a biological sample, and it provides metabolic information that reflects the environmental and physiological status of the samples [21]. Metabolomics is widely applied in drug evaluation and discovery, searching for clinical biomarkers, and toxicology assessments. At present, studies focusing on the toxic effects of ZEA on cells or biological fluids are scarce. Therefore, in the current study, the relationship between ZEA exposure and *in vitro* porcine follicle growth was investigated with focus on the metabolomics changes (Figure S1).

## RESULTS

### ZEA exposure affected porcine follicle growth *in vitro*

**Figure 1:**
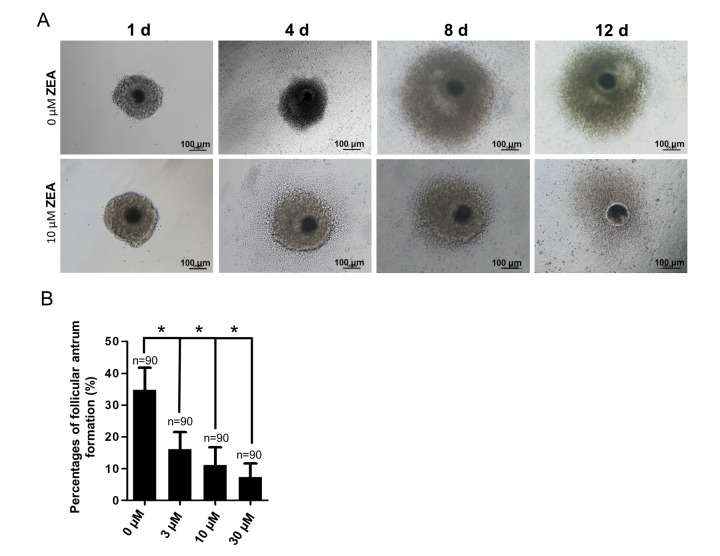
**Effect of zearalenone (ZEA) on *in vitro* ovarian follicle culture.** (**A**) The oocyte-granulosa cell complex (OGC) growth status (1 d, 4 d, 8 d and 12 d) in an *in vitro* culture model in both control group and 10 µM ZEA treatment group, in day 8 the follicle starts to form an antrum. The percentages of antrum follicle formation following treatment with 0, 3, 10 and 30 µM of ZEA are shown (**B**). * means *P* < 0.05, n = numbers of OGCs.

To analyze the effect of ZEA exposure on follicular growth, an *in vitro* model of follicle development was established. The results depicted in Figure 1A. It showed that, generally, the oocyte-granulosa cell-complexes (OGCs) could grow with GC proliferation and form an antrum on one side following culturing for 8 d. At 12 d, the antrum would enlarge and surround the oocyte. Compared with the control group, the percentage of antrum formation was significantly decreased dose-dependently from 34.75% (control) to 16.10% in the 3 μM ZEA treatment group, 11.08% in the 10 μM ZEA treatment group, and 7.30% in the 30 μM ZEA treatment group, respectively (*P* < 0.05; Figure 1B).

### Distinct granulosa cell metabolic profiling was between ZEA treated and control groups

**Figure 2:**
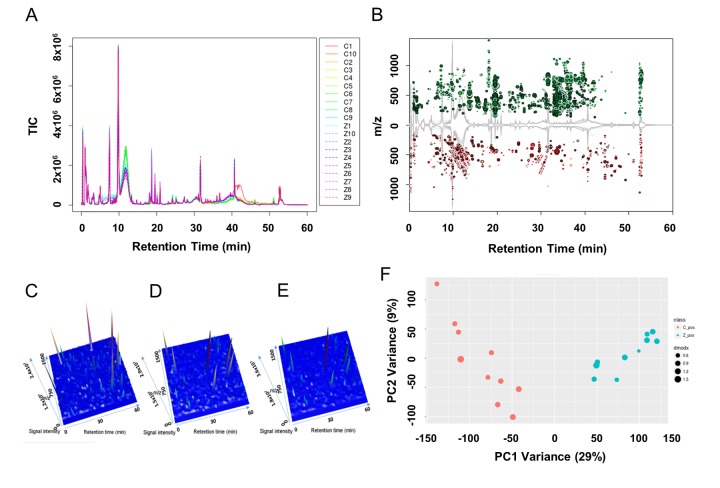
**Metabolic profiles of granulosa cell (GC) media within or without ZEA-treatment.** Utilizing the UPLC-QTOF detection method, total ion current diagrams vs. retention time of each group of GC media are shown (**A**), with Z1-Z10 mean 10 µM ZEA groups 1-10 and C1-C10 mean control groups 1-10 (0 µM ZEA). (**B**) Significantly different metabolites between the culture media with or without 10 µM ZEA treatment were marked in the cloud plot of mass to charge ratio (m/z) vs. retention time. Red point mean the ion of certain m/z showed decrease in ZEA groups compare with control groups. And green point in return, with the size of the point reflect the significance. Three-dimensional peak diagram of retention time, signal intensity, and m/z in blank control media (**C**), GC media without ZEA (**D**) or with ZEA treatment (**E**). (**F**) Principle component analysis (PCA) of the metabolic profiles among each sample between GC media without (red point) or with (cyan point) ZEA treatment.

In the vehicle control and the 10 μM ZEA treated granulosa cell culture media groups 29721 features in positive mode were detected. Among all the detected features, 5215 features had extremely different content among them (Figure 2A and 2B; *P* ≤ 0.001) with a signal intensity fold change of >= 1.50 or <= 0.67. In the 3-D plots, the results showed that the culture media of GCs supplied with 10 μM ZEA or vehicle control had similar patterns, which were different from the blank media (Figure 2C-2E). In order to evaluate the signature of each metabolic profile, principle component analysis (PCA) was performed (Figure 2F). The result showed that samples from the control and 10 μM ZEA treated GC media could be differentiated and clustered separately. Querying the METLIN metabolite database, there were a series of phospholipids and fatty acids with significant changes in the 10 μM ZEA treated GC media compared to that of control group. The adducts of phosphatidylcholine (PC) or phosphatidyl ethanolamine (PE) (C_42_H_82_NO_8_P) with the molecular weight of 760.5772 were more abundant in 10 μM ZEA treated GC media, the same trend like an adduct of eicosadienoic acid or octadecadienyl acetate with the molecular weight 305.2432 (C_20_H_36_O_2_) (Table S4 and Table S5).

After data processing, in the small and large FF groups, it was hard to point out the different metabolic features from the total ion chromatograms (Figure S3A). Therefore, we statistically analyzed the retention time and mass charge ratio (m/z) of the two groups. In total 3238 features were detected in positive mode, among them, 579 features with extremely differential content between them were detected (Figure S3B). In addition, 3-D plots showed the main peaks generally formed similar patterns in the small (Figure S3C) and large (Figure S3D) FF groups. PCA showed that the samples from small and large follicles could be differentiated (Figure S3E). To investigate representative differential metabolites between small and large FF, the most differentially expressed metabolites were analyzed. Interestingly, after querying the METLIN metabolite database, the top ten significantly up-regulated features were lecithin class enriched (Table S6 and Table S7).

### ZEA led to platelet-activating factor (PAF) and lyso phosphatidylcholine (LPC) depletion in the GC media

**Figure 3:**
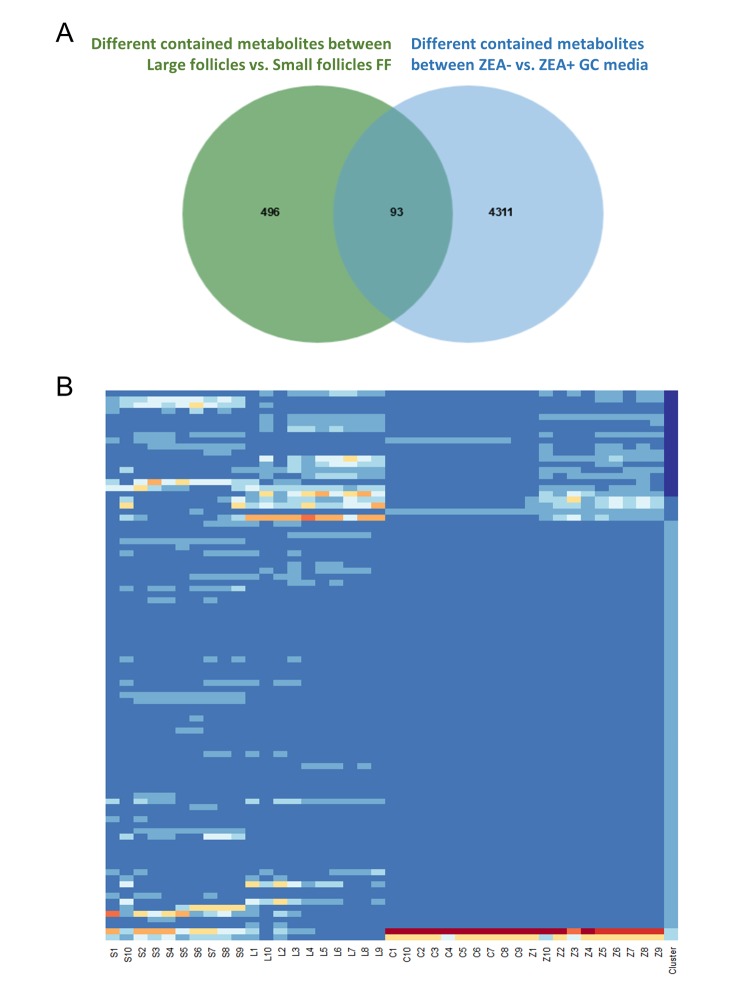
**Co-exist metabolites between GC media and Follicular fluid (FF).** Number of differential contained metabolites detected in the antral FF (green circle), and GC media (blue circle), the overlap showing co-existing metabolites in the FF and GC media (**A**). Heatmap showed the relative content of each co-existing metabolites in each group (**B**).

**Figure 4:**
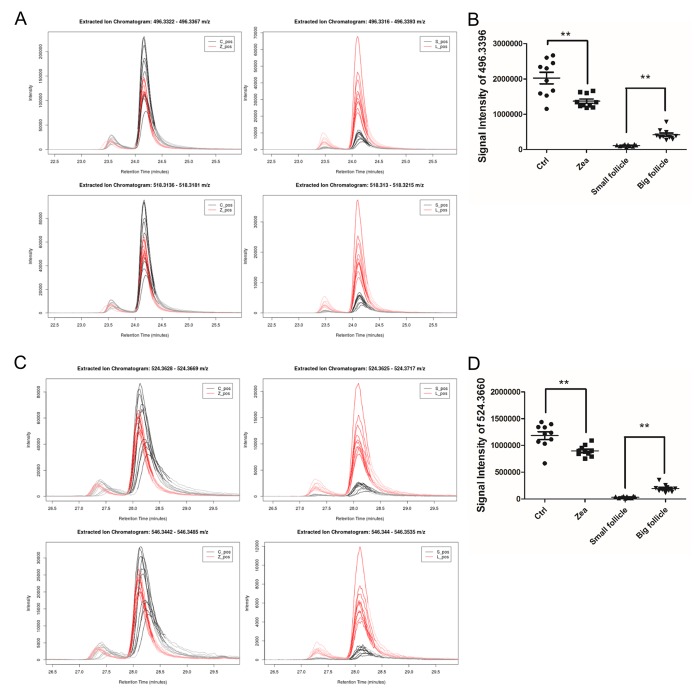
**Relative content of two potential phospholipids in each group.** The relative content of two metabolites adduct [M+H]^+^ (upper in **A**, **C**) and [M+Na]^+^ (below in **A**, **C**) with the molecular weights of 496.3324 (**A**) and 518.3168 (**C**). The left curve plots show the relative content of m/z = 496.3324 and 518.3168 in each GC media sample (**A** and **C**), while the right curve plots showed the two metabolites in each FF sample (**A** and **C**). The signal intensity of the two metabolites UPLC peaks reflect the relative content of the m/z= 496.3324 (**B**) and 518.3168 (**D**) in each group. ** means *P* < 0.01.

In total, 112 features with a trend were filtered (Table S8). After querying the potential annotations in the METLIN database, 93 potential metabolites could be queried (Figure 3A). The components of each metabolite among groups were shown in Figure 3B. We noticed the features with the molecular weight of 496.3351 (518.3168, with the formula of the parent ion C_24_H_50_NO_7_P), 524.3660 (546.3475, with the formula of the parent ion C_26_H_54_NO_7_P) (Figure 4A and 4C), 522.3505 (544.3321, with the formula of the parent ion C_26_H_52_NO_7_P) (Table S8 and Table S9). It seems that these chemicals could have a similar structure as a phospholipid (Table S8 and Table S9). All three metabolites significantly were accumulated during antrum follicle growth, while were depleted in the 10 μM ZEA treated GC culture media (*P* < 0.01; Table S8; Figure 4B and 4D).

**Figure 5:**
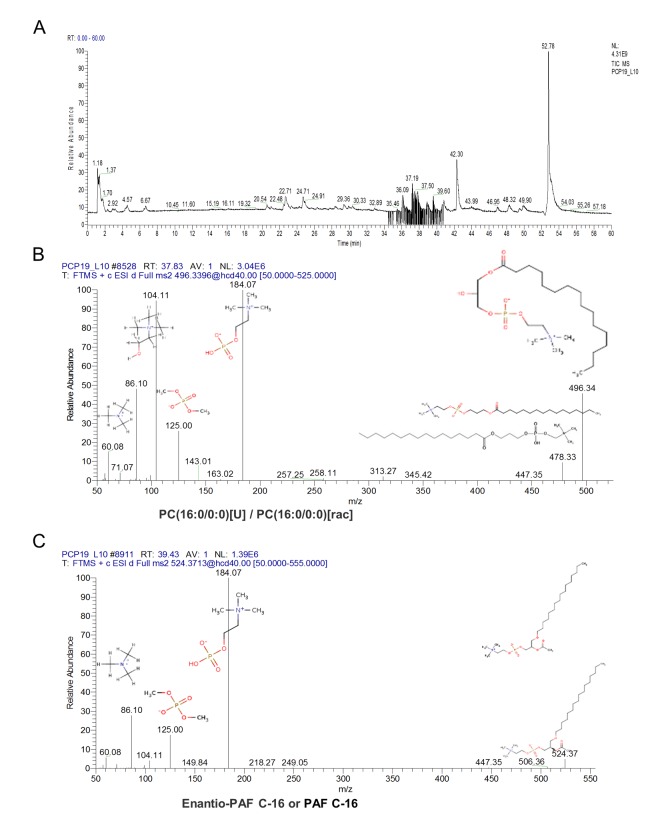
**The characteristic of the altered content lyso phosphatidylcholine (LPC) and platelet-activating factor (PAF).** Orbitrip coupled with HPLC detected altered content metabolites with the m/z = 496.3324 and 518.3168 that were continuously eluted in 34 - 41 minutes (**A**). The structure information obtained from Orbitrip showed m/z = 496.3324 is a kind of LPC (**B**), and m/z =518.3168 is a kind of PAF (**C**).

To accurately identify these ions and proposed the structures, we used Q Exactive Obitrap MS in selecting ion monitor mode to detect the daughter ion. In the total ion current plots, all 3 features were eluted between 34 - 41 min (Figure 5A). From the daughter ion information, the exact structure of the C_24_H_50_NO_7_P and C_26_H_54_NO_7_P were presented. C_24_H_50_NO_7_P with an annotation of PC (16:0/0:0) [U] / PC (16:0/0:0) is a phospholipid derivative called LPC (Figure 5B). C_26_H_54_NO_7_P with an annotation of enantio-PAF C-16 or PAF C-16 is a phospholipid activator called platelet-activating factor (Figure 5C). There was a significant increase in the content of LPC and PAF in the FF during porcine follicle growth, and 10 μM ZEA could lead to a depletion of LPC and PAF in the cultured GC media (*P* < 0.01; Figure 4B and 4D).

### LPC could protect the inhibition of ZEA on oocyte maturation

In our previous study, we found ZEA could inhibit porcine oocyte maturation [22]. Taking into account the excessive depletion of PAF and LPC in 10 μM ZEA treated GC culture media and the accumulation of these two compounds during antral follicle growth we wanted to determine whether supplementation with exogenous LPC or PAF could protect the inhibition caused by ZEA on antral follicle growth and oocyte maturation.

**Figure 6:**
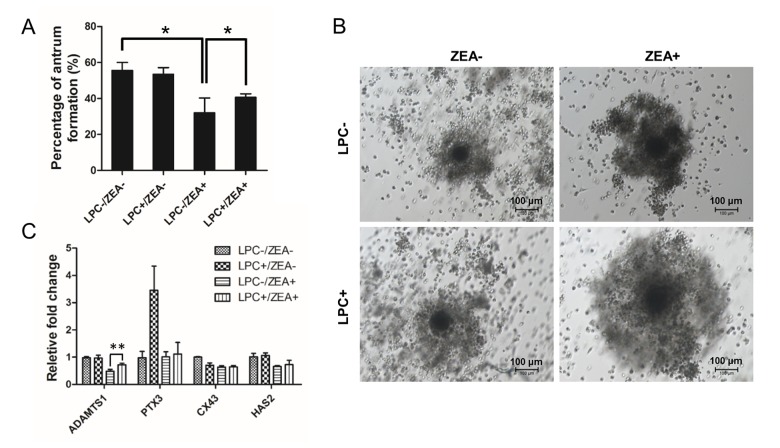
**The effect of LPC co-treated ZEA on the follicular antrum formation and** **cumulus cell (CC) expansion.** (**A**) The effect of supplementary 10 µg/mL LPC on the formation of follicular antrum of 10 µM ZEA treated OGCs after 12 d culture. (**B**) Morphology of CC expansion status in 10 µg/mL LPC and 10 µM ZEA co-treated conditions. (**C**) The effect of supplementary10 µg/mL LPC on CC expansion related gene expression of 10 µM ZEA treated cumulus oocyte complex (COCs). * or ** mean *P* < 0.05 or 0.01.

During oocyte maturation process, cumulus cells (CCs) undergo an obvious expansion process with extracellular matrix (ECM) composition. CC expansion need rapid HAS2 gene expression to synthesis hyaluronic acid-rich ECM, PTX3 gene expression to promote ECM stabilization, and gap junction protein encode CX43 gene expression [22]. For OGCs growth, 10 µg/mL LPC could protect the inhibition of 10 µM ZEA on antrum formation to some extent (*P* < 0.05; Figure 6A). The morphology of the cumulus-oocyte complexes (COCs) showed an impairment in CC expansion following ZEA treatment, while in the 10 µg/mL LPC and 10 µM ZEA co-treated groups, it seemed like LPC could at least partially protect CCs from the damage on expansion (Figure 6B). Gene expression results were also proved that CC expansion related gene ADAMTS1 expression was depressed by 10 µM ZEA treatment, the gene which was need for normal ovulation, and could be partially protected by 10 µg/mL LPC co-treated (*P* < 0.01; Figure 6C). ZEA also slightly repressed CX43 and HAS2 gene expression but not obvious, and almost no effect on PTX3 expression. We co-treated COCs with 10 µg/mL or 100 µg/mL PAF and 10 µM ZEA, the morphology results showed that PAF were unable to remit the poor CC expansion status (Figure S4A).

**Figure 7:**
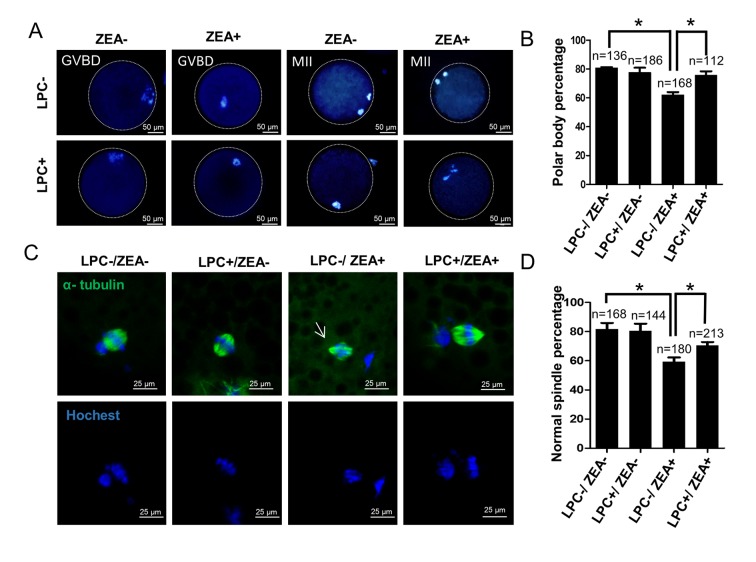
**The effect of LPC co-treated ZEA on oocyte maturation.** (**A**) The effect of 10 µg/mL LPC and 10 µM ZEA on oocyte maturation *in vitro*. (**B**) Polar body percentage of oocytes in 10 µg/mL LPC and 10 µM ZEA co-treated group. (**C**) The effect of 10 µg/mL LPC and 10 µM ZEA on the spindle assembly of oocytes, and the arrow shows the abnormal spindle assembly. (**D**) Normal spindle assembly percentage in 10 µg/mL LPC and 10 µM ZEA co-treated group. * means *P* < 0.05.

Next, we investigated the effect of 10 μM ZEA and 10 µg/mL LPC co-treatment on germinal vesicle breakdown (GVBD) and polar body extrusion (MII) (Figure 7A and 7B, FFigure S4B). Compared with the ZEA treated group (62.0 ± 5.02%), LPC could significantly protect the inhibition of ZEA on oocyte maturation (75.6 ± 6.86%; *P* < 0.05; Figure 7B). We further detected the effect of ZEA on the meiosis spindle assembly of oocytes. The result showed that compared with control group (81.7 ± 7.37%), ZEA treatment significantly decreased the percentage of normal spindle assembly in oocytes (59.3 ± 5.13%; *P* < 0.05), and LPC could protect the inhibition of ZEA on the spindles assembly (70.7 ± 3.79%, *P* < 0.05; Figure 7C and 7D).

### LPC could protect the inhibition of ZEA on parthenogenetic activation of porcine oocytes

**Figure 8:**
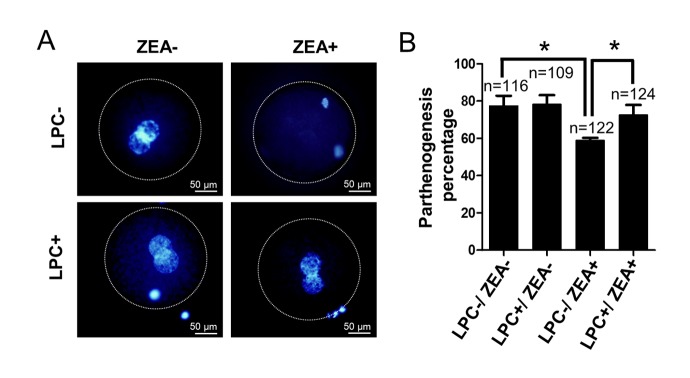
**The effect of LPC co-treated ZEA on parthenogenetic activation of oocytes.** (**A**) The effect of supplementary 10 µg/mL LPC and 10 µM ZEA on parthenogenetic activation of mature oocytes *in vitro*. (**B**) The parthenogenesis percentage of mature oocytes in 10 µg/mL LPC and 10 µM ZEA co-treated condition. * means *P* < 0.05.

In order to further study the effect of LPC on oocyte development *in vitro*, we verified the effect of LPC on parthenogenetic activation of mature oocytes *in vitro*. The result showed that compared with the control group (77.7 ± 8.96%), the parthenogenetic activation percentage of 10 μM ZEA treatment group was significantly decreased (59.0 ± 2.27%; *P* < 0.05; Figure 8A and 8B). It is interesting to note that the parthenogenetic activation percentage of oocytes reach to 72.6 ± 9.26% in 10 µg/mL LPC and 10 μM ZEA co-treated group (*P* < 0.05; Figure 8B). Compared with control and ZEA treatment groups, LPC treatment promoted cleavage and blastocyst development of parthenogenetic embryos, but not significantly (Figure S4C-S4F).

## DISCUSSION

Numerous studies have concentrated on the toxic effects or endocrine disrupting effects on female reproduction of ZEA and other endocrine disrupter. In addition, some studies investigated the metabolic profiling related to the reproductive process in humans and animals. However, there are few studies investigating the relationship between the effect of ZEA on reproductive health and the metabolic process.

In this comparative toxicological metabolomics study, we focused on the metabolomics changes caused by ZEA exposure in GC culture media. ZEA, as an endocrine disruptor and reproductive toxicant, its major active metabolite α-ZOL, combined with other mycotoxins could alter the steroidogenesis in follicles [24,25]. It is highly likely that ZEA may affect lipid metabolism in the GCs. The metabolites of FF constitute the microenvironment during which the oocyte acquires competency. We hypothesize that ZEA alters the concentration of metabolites during follicular growth which could influence the folliculogenesis and/or oocyte quality. Therefore, we compared the concentration of metabolites in small and large antral follicles to identify those that accumulate during follicle growth progression. We were then able to identify those that were significantly depleted in ZEA exposed groups. Our results showed that ZEA could alter the GC metabolic profile, and these kinds of models could easily be differentiated even with the heterogeneous factors existing in media samples. We found that ZEA altered the phospholipid steady state of the GC culture media. Potential PA with a molecular weight of 702.5563, and phosphatidic glycerol with a molecular weight of 650.4159 and 782.5462 in the ZEA treated GC media were lower than in the control group. However, potential PC or PE with a molecular weight of 759.5778 was much more abundant in the ZEA treated group than in the control. From the results of a CC lipid profile study, after an IVF cycle, pregnant and non-pregnant patients could be differentiated as those with a relatively low content of PC (m/z = 886.6428), PE (m/z = 822.4423) and PS (m/z = 750.4191 and 806.4842) which can be potential biomarkers of pregnancy [26]. Another study using Wistar rats indicated that another endocrine disruptor chemical, flavones, altered metabolomics in the FF dose dependently [27].

From the result of our *in vivo* FF metabolic assessment, intriguingly, comparing the metabolome FF isolated from small and large antral follicles, phospholipids were accumulated in the large FF group of multiple species. Potential PC and LPC (m/z = 521.3481, 523.3638, 519.3325, and 495.3325) were more abundant in the large FF group, indicating that PC synthesis may be involved in the follicle growth and oocyte maturation process. In addition, PCA analysis showed that, the metabolite composition of the FF was different and can be clustered by follicular development. For the follicle development physiology, the metabolite concentrations in the FF undertake dramatic changes, which are affected by the luteinizing hormone (LH) surge in the oestrus cycle [28]. Compared with the dominant follicle in heifers, saturated fatty acids such as palmitic acid and stearic acid are in much higher concentrations in lactating cows as detected by GC-MS [28]. Interestingly, heifers have a much higher fertility rate compared to lactating cows. In an nuclear magnetic resonance metabolomics study in sows, it was found FF from small and large follicles with distinct metabolomics signatures, glucose, lactate, and five amino acids were changed significantly [29]. It is very likely that during the ovarian follicle enlargement process the environmental and physiological factors change the capillary permeability of ovarian follicles and the secretion from follicular cells which subsequently alters the metabolite composition in FF.

Next, we compared the co-existence of metabolites in the GC media and FF, and noted that of the 112 co-existing metabolites, potential PC metabolites are made up a great proportion. Generally, PC is the lipid subclass, which constitutes 40 - 50% of total phospholipids. Among the abundant co-existing phospholipids, C_24_H_50_NO_7_P and C_26_H_54_NO_7_P accumulated during follicle enlargement and were excessively depleted in the ZEA treated GC media. It was found these two metabolites were LPC and PAF, respectively, by Orbitrap qualitative determination. When we added the two chemicals into the oocyte maturation media and follicle growth media, respectively, with ZEA co-treatment, it was found that LPC and not PAF could remit or partly remit the inhibition of ZEA on oocyte maturation, CC expansion, and follicular antrum formation. Recently, an IVF metabolomics study reported that LPC secreted by CCs during the process may act as a paracrine factor on oocytes and be involved in the acrosomal reaction [30]. In mouse hepatocytes, activated peroxisome proliferators-activated receptors could increase LPC (16:0) production [31]. In human, lysophospholipase D converts unsaturated LPC to LPA [32]. LPA, acting as a YAP (yes associated protein) co-activator, could promote the oocyte maturation and blastocyst development [33,34]. In current study, YAP proteins in GCs were decreased in the ZEA treatment group, while it was partially recovered by LPC and LPA addition (Figure S5). However, LPA seems to be more effective in the protection of the damage on COCs and GCs by ZEA. The conversion of LPC to LPA is likely to be involved in this process. Further research should be conducted to investigate whether the conversion of LPC to LPA is involved in the ZEA-induced oocyte damage remission process.

In summary, this study shows for the first time that ZEA generally alters GC metabolic process, and induces excessive depletion of LPC, and exogenously addition of LPC could remit ZEA induced inhibition and damage on the oocyte maturation process.

## MATERIALS AND METHODS

### Ethics statement and experimental design

All procedures performed in this study were reviewed and approved by the Ethics Committee of Qingdao Agricultural University.

As shown in Figure S1, the aim was to discover the most altered metabolites in the microenvironment of ZEA treated GCs and then screen the content for changed metabolites during the antral follicle growth process *in vivo*. Comparing these data, we aimed to determine the adaptive changes in the GC culture media, which could be indicative of the potential negative effects of ZEA on the viability of GCs. Metabolites were detected based on mass by using a UPLC-QTOF and qualification by using a UPLC MS/MS instrument (Figure S1).

### Follicle culture *in vitro*

Ovaries from unmated young gilts were collected from Qingdao Fu Wan Pig Production Cooperation (Qingdao, China). In order to investigate the effect of ZEA on follicular growth, we adapted an OGC culture method with minor modification [35]. Briefly, OGCs with complete GC layers were collected. After being washed three times in Dulbecco’s modified eagle medium (DMEM, Hyclone, SH30022.01, Beijing, China) three times, we separately cultured each OGC in 200 μL culture media in 96-well dishes (Corning, CLS3599, NY, USA) for 12 d within 38.5 ºC, 5% CO_2_, and 100% humidity conditions. The composition of the OGC culture media was DMEM, supplemented with 1 μg/mL estradiol (E2) (Sigma-Aldrich, E2758, MO, USA), 0.5 μg/mL FSH (R&D, 5925-FS, MN, USA), 10 mM taurine (Sigma, T8691), 2 mM hypoxanthine (Sigma, H9636), 2% polyvinylpyrrolidone (Sigma, PVP40), 1% insulin transferrin selenium (ITS, Gibco, 41400045, USA), 3 mg/mL bovine serum albumin (BSA, Sigma, A1933), and 10% fetal bovine serum (FBS, Gibco, 10099-141). Every 4 days, 100 μL old media was replaced with fresh media in each well.

ZEA was purchased from Sigma-Aldrich (Z2152), and diluted by DMSO at 2000 μM concentration and stored at - 20 ºC in darkness. For assessing the effects of ZEA on follicular growth, each well contained 0, 3, 10 or 30 μM with equal solvent concentration (0.5% DMSO).

### FF and GC media sample collection for metabolomic analysis

FF from small (< 2.0 mm) and large follicles (> 3.0 mm) was randomly collected (Figure S2A). For each ovary, we aspirated the largest and smallest 10 follicles with an 18 G injection syringe. Five ovarian FF absorption samples were pooled (each FF sample include fluid from 50 follicles) and centrifuged at 1500 rpm, 4 ºC for 10 min and then 50 μL of the supernatant was transferred into a 1.5 mL microcentrifuge tube, and then, 150 μL deionized water and 600 μL methanol were added and the mixture was mixed thoroughly. After centrifugation at 12000 rpm, 4 ºC for 15 min, the suspension was transferred into a new microcentrifuge tube, and kept frozen at - 80 ºC until required for analysis. In total, FF samples from 10 ovaries were obtained for detection.

GCs were collected from small follicles (< 2 mm). The cell pellet was resuspended with PBS for washing, centrifuged at 1500 rpm for 5 min. The recovered cells were then cultured in M199 (Hyclone, SH30253.01B) +10% FBS + 100 IU penicilin-streptomycin media, in 38.5 ºC, 5% CO_2_ condition. After 24 h the media was changed and at 72 h, the cells were transferred into 24-well plate, with 1×10^5^ cells/well. After being cultured for 24 h, the media was removed and the cells were washed 3 times with PBS and then, 1 mL fresh media supplement with 5 μL DMSO solvent (vehicle control) or ZEA 10 μM (with equivalent DMSO solvent). After being treated for 24 h, the media was collected and centrifuged at 1500 rpm, 4 ºC for 10 min. After that, 50 μL media sample was transferred into a new microcentrifuge tube and 150 μL deionized water and 600 μL methanol added and thoroughly mixed. The mixture was centrifuged at 12000 rpm, 4 ºC for 15 min, the suspension transferred into a new microcentrifuge tube and then kept frozen at - 80 ºC until required for mass spectrometry detection. After 3 collections, 10 groups of control and 10 groups of ZEA treated GC media were obtained.

### UPLC-QTOF and UPLC MS/MS metabolomics analyze of collected samples

In this study, we utilized ultra-performance liquid chromatography (Ultimate 3000UPLC, Dionex, USA)-high resolution mass spectrometry (maXis, Bruker, Germany) to identify the characteristic MS peaks of each group. For FF extraction, a 3 μL aliquot was injected into a 2.1×150 mm Agilent Zorbax SB-C18 5 μm column, using a Dionex Ultimate 3000 UPLC for LC-MS (Dionex, USA). The column was maintained at 40 ºC. The solvent gradients are shown in Table S1. With the flow rate set at 0.2 mL/min. Mass spectral analysis was performed using QTOF electrospray ionization parameters operating in positive ion mode (ESI+). Argon was used as the collision gas, while nitrogen was used as the nebulizing gas. The mass capillary voltage was set at 4500 V. The temperature of the TOF heater was set at 180 ºC, with the flow rates of the dry gas set at 6.0 L/min. Full scan data was collected in the range from 100 to 1500 mass to charge ratio (m/z).

However, as some of the fragments detected by high resolution MS still could not qualify the compounds particularly with a mass which contained multiple isomers, the exact qualification utilizing Orbitrap (Q-Exactive^TM^, Thermo Fisher, USA) was performed for the intrigued ions, because the triple quadrupole MS can provide abundant daughter ion information. The conditions of the solvent gradients of the Orbitrap coupled UPLC are shown in Table S2 at a flow rate of 0.2 mL/min.

### Metabolomics data preprocessing

The UPLC-QTOF raw data was preprocessed and analyzed by correcting the data with sodium formatting and transforming the raw data into mzXML using CompassXport software. After this, the R package xcms was used to analyze the data online (https://xcmsonline.scripps.edu/). The raw data went through filter and identify peaks, samples grouping, retention time correction, filling the missing peak, and was statistically analyzed. For identifying significantly different metabolites, a personal R script was used to calculate. For the representative features’ identification, the mass weight was inputted into the METLIN database (https://metlin.scripps.edu/) for searching. The parameter adduct ion was supposed to be likely to M+H^+^ or M+Na^+^, with the fault-tolerant of ± 10 ppm. The Orbitrap data acquisition utilized xcailibur software.

### Oocyte *in vitro* maturation and polar body extrusion assessment

COCs aspirated from antral follicles (3 - 6 mm) surrounded with compact cumulus cells were cultured in oocyte maturation media, with 0.57 mM cysteine (Sigma, A9165), 0.5 μg/mL FSH, 0.1 IU/mL LH (Sigma, L5269), 10 ng/mL epidermal growth factor (EGF) (R&D, 2028-EG), and 10% volume ratio FF in the M199 media. About 15 COCs were cultured in an 80 μL oocyte maturation media drop covered with mineral oil on 3.5 mm plates in the 39 ºC, 5% CO_2_, and saturated humidity condition. After 44 h maturation, polar body extrusion was counted to evaluate the effect of different exogenous metabolites supplement on ZEA treated oocytes. Denuded oocytes were fixed with 4% formaldehyde in PBS, then stained with Hoechst 33342 for 5 min, and mounted on slides.

### Assessment of meiotic spindle assembly

After cultured for 44 h, Denuded oocytes were fixed with 4% paraformaldehyde, after washed 3 times with PBS and permeabilized with 1% PBST for 30 mins. After washed 3 times with PBS, oocytes were blocked with 1% BSA for 1 h at room temperature, and then incubated with the α-tubulin antibody (Sigma, T6199) diluted 1: 200 for 1 h. Then washed 3 times with PBS, oocytes were incubated the second anti - fluorescein isothiocyanate (FITC) conjugated goat anti-Rabbit IgG (Beyotime, A056, Nantong, China), for 1 h. After washed 3 times, oocytes were stained with Hoechst 33342 for 5 min in dark, and then washed 3 times in PBS, and mounted on slides.

### Parthenogenetic activation of oocytes and parthenogenetic embryo culture

Embryo culture medium (PEM-5 including 108 mM NaCl, 10 mM KCl, 0.35 mM KH_2_PO_4_, 0.4 mM MgSO_4_-7H_2_O, 25.07 mM NaHCO_2_, 0.2 mM Na pyruvate, 2.0 mM Ca (lactate)-5H_2_O, 2.0 mM glutamine, 5.0 mM hypotaurine) and oocyte activating medium (PEM-5, 0.6 mM L-cysteine, 4 mg/mL BSA, 7.5 μg/mL CB (Sigma, C6762)) were balanced 2 h in advance. In addition, the electro-active solution (4.74 mg/mL mannitol, 0.01 mg/mL CaCl_2_, 0.02 mg/mL MgSO_4_, 0.02 mg/mL HEPES, 1 μg/mL BSA) was also preheated in advance. After COCs cultured for 42 h, denuded oocytes were washed 5 times with electro-active solution, and activated for 60 μs at 110 V/mm under Cell fusion apparatus (BTX, Gemini X2, Harvard Apparatus). Then oocytes were cultured with oocyte activating medium for 3 h, and finally transferred to embryo culture medium.

### RNA purification, reverse transcription and quantitative PCR

After maturation, approximately 100 COCs in each group were used for RNA purification. RNA was purified by using an RNAprep pure Tissue Kit (Tiangen, DP431, Beijing, China) according to the manufacturer’s instructions. Reverse transcription was conducted by using a Transcript One-Step gDNA Removal and cDNA Synthesis SuperMix Kit (TransGen, AT311, Beijing, China). Quantitative PCR was performed as described in our previous study [22]. The Primer information is given in Table S3. The amplification efficiency of the primers was tested (Figure S2B). Relative level of gene expression was calculated by using the 2^-ΔΔCt^ method.

### Immunofluorescent staining of YAP and ethylene diurea (EdU) in porcine COCs and GCs

After culture for 44 h, COCs were fixed with 4% paraformaldehyde (pH 7.4) for at least 30 min, at room temperature, followed by one wash in PBS and incubating with 3% BSA. After permeabilized with 1% PBST for 20 min, COCs were stained according to the protocol of Click-iTR Plus EdU Alexa FluorR 488 Imaging Kit (Thermo, C10637, USA). Then COCs were washed in PBS and saturated with PBS supplemented with 3% BSA for 5 min. Followed blocking with 10% goal serum (Boster, AR0009, Wuhan, China), COCs were incubated with the primary antibody-YAP (Bioworld, Q295, Nanjing, China) diluted 1: 200 for 1 h. After three washes in PBS, the COCs were labeled with FITC-conjugated secondary antibody diluted 1:100 for 1 h at room temperature. The nucleus of COCs were evaluated by staining with Hoechst 33342 for 5 min. Following extensive washing, samples were mounted between a coverslip and glass slide. COCs were observed under a fluorescence microscope (Olympus, BX51, Japan).

After culturing in GC media for 72 h, GCs were centrifugalized at 1500 rpm for 5 min and fixed with 4% paraformaldehyde (PH 7.4) for at least 30 min. GCs were mounted on glass slides and washed with PBS and 3% BSA for 5 min. Followed permeabilized with 1% PBST for 20 min, GCs were stained with EdU Kit. GCs were washed in PBS and blocked with PBS supplemented with 3% BSA for 5 min. YAP-stain was performed the same with COCs. Finally, GCs were examined using a confocal microscope (Leica, SP5, Germany).

### Western blot

After treatment by different concentrations of LPA, LPC or ZEA for 72 h, porcine GCs were collected for western blotting analysis according to standard methods [36]. The protein of each group was separated by SDS-PAGE and transferred to PVDF membranes. Then using 5% BSA dissolved in PBST with 0.05% Tween-20 (PH 7.4) to block for 1.5 h, the membranes were incubated with anti-GAPDH (ImmunoWay, YM3040, USA), anti-YAP (Bioworld, Q295), anti-ACTIN (Abcam, ab8226, USA) overnight, at 4 ºC. Followed be washing three times with TBST, the membranes were incubated with secondary antibodies (Beyotime, A0208 or A0216) at a dilution of 1:2000 in TBST. The band intensity was quantified using ACTIN or GAPDH as internal control and measured with IPWIN software.

### Statistical analysis

For test, at least three replicates were performed. Data is shown as mean ± SEM. Among groups, significant differences were tested using Student’s t-test or one-way analysis of variance (ANOVA) coupled Tukey HSD multiple test. *P* < 0.05 was considered as significant. Graphs and charts were plotted using R 3.1 or Graphprism 5.0.

## Supplementary Material

Supplementary Figures

Supplementary Tables 1-3

Supplementary Table 4

Supplementary Table 5

Supplementary Table 6

Supplementary Table 7

Supplementary Table 8

Supplementary Table 9
