# Supplementary material for: Phosphatidylcholine could protect the defect of zearalenone exposure on follicular development and oocyte maturation
Source: Aging (Albany NY). 2018 Nov 25;10(11):3486–506. doi: 10.18632/aging.101660 (PMC6286824; doi:10.18632/aging.101660)
Supplement: Supplementary Figures [file aging-10-101660-s001.pdf]

SUPPLEMENTARY FIGURES

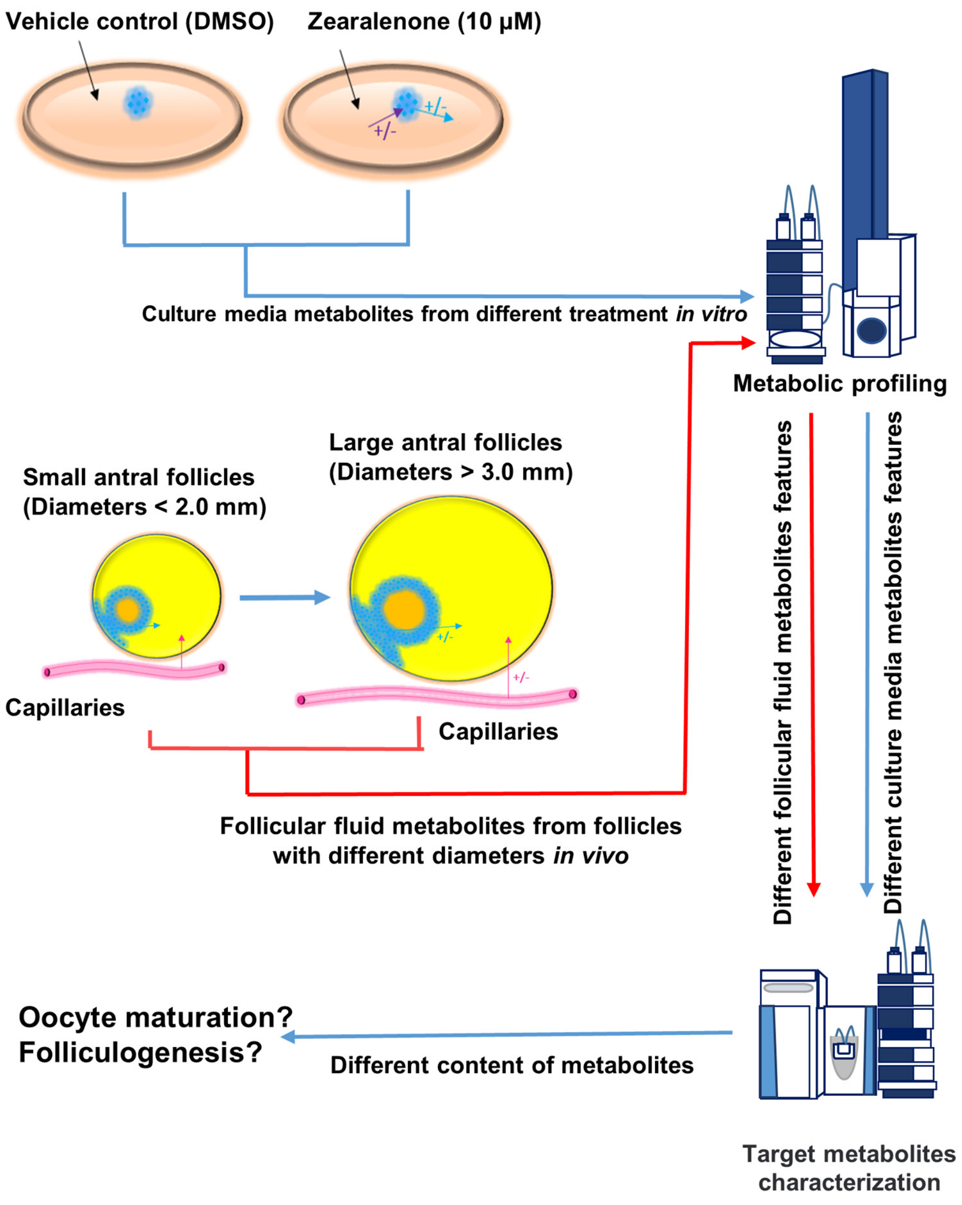

Figure S1. Schematic diagram of the experimental design. In order to compare the changes of metabolite profile composition, using the UPLC-QTOF methods, we detected the metabolomic profiles of small and large antral FF, and GC media with (+) or without (-) ZEA treatment. With regard to the targeted metabolic features, we used UPLC-MS/MS to perform structure qualitative analysis. Finally, target metabolites were exogenously added to verify whether they play a role on the effect of ZEA on oocyte or ovarian somatic cells.

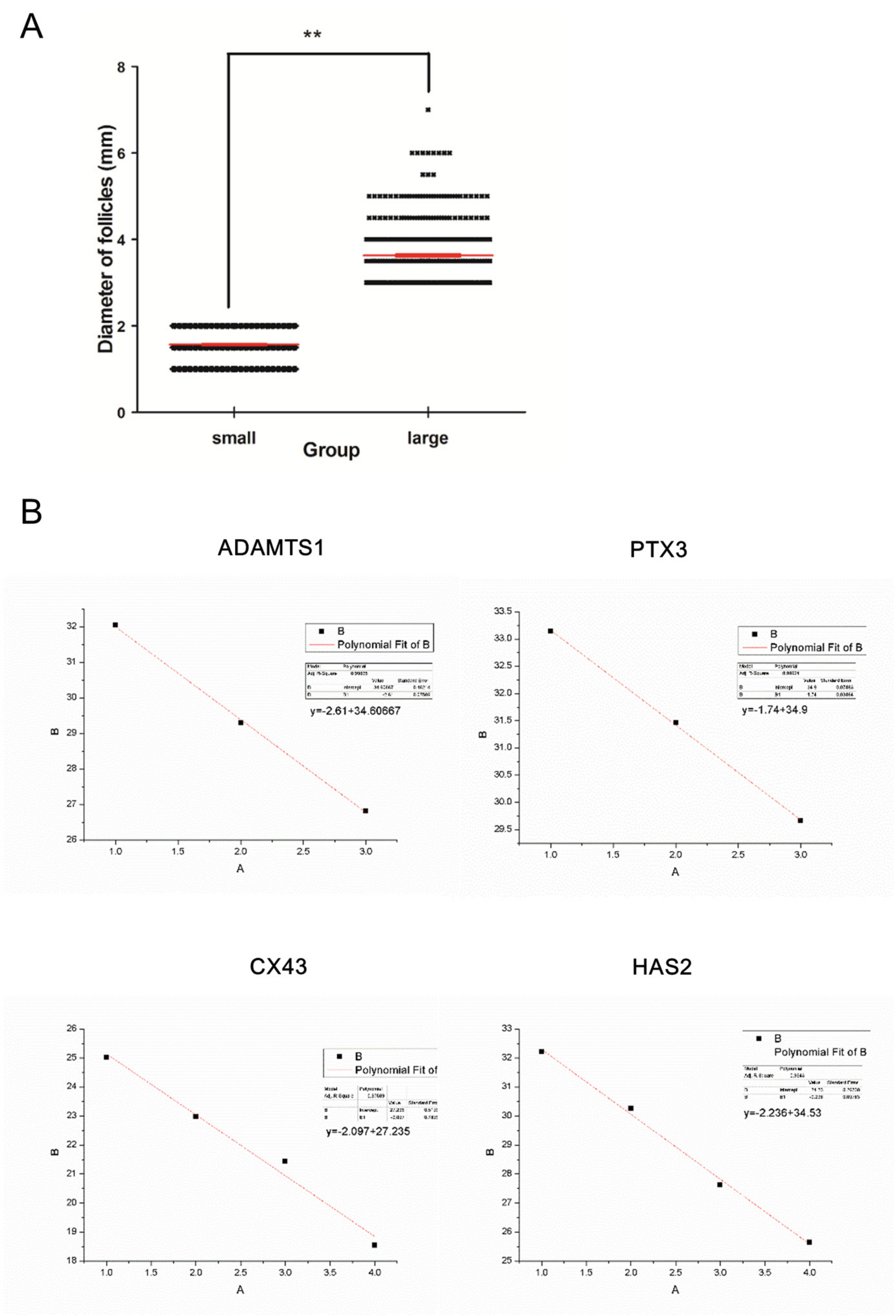

Figure S2. The diameter of antral follicles and the amplification efficiency of each primer. (**A**) The diameter of antral follicles used for FF metabolite profile detection. (**B**) The amplification efficiency of the primers of ADAMTS1, PTX3, CX43 and HAS2 genes.

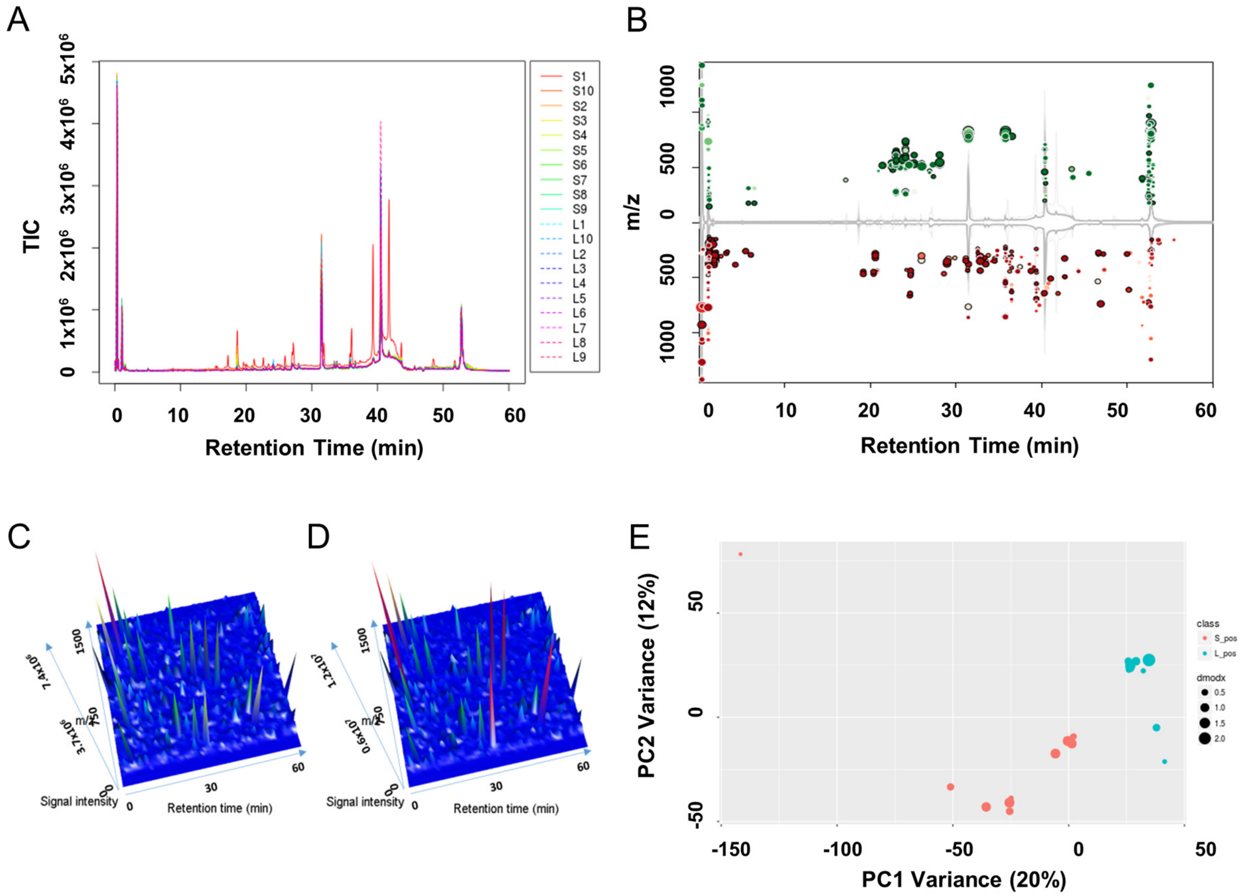

Figure S3. Metabolic profiles of FF from small and large antral follicles. Utilizing UPLC-QTOF detection, total ion current diagrams of the FF in small and large antral follicle are shown (**A**) , with S1-S9 mean small antral follicle groups 1-9 and L1-L10 mean large antral follicle groups 1-9. Significantly different metabolites between small and large antral follicle isolated FF were marked in the cloud plot (**B**). Three-dimensional peak diagram of retention time, signal intensity, and m/z in small (**C**) and large (**D**) FF samples. PCA of the metabolic profiles among each sample between small (red point) and large (cyan point) FF (**E**).

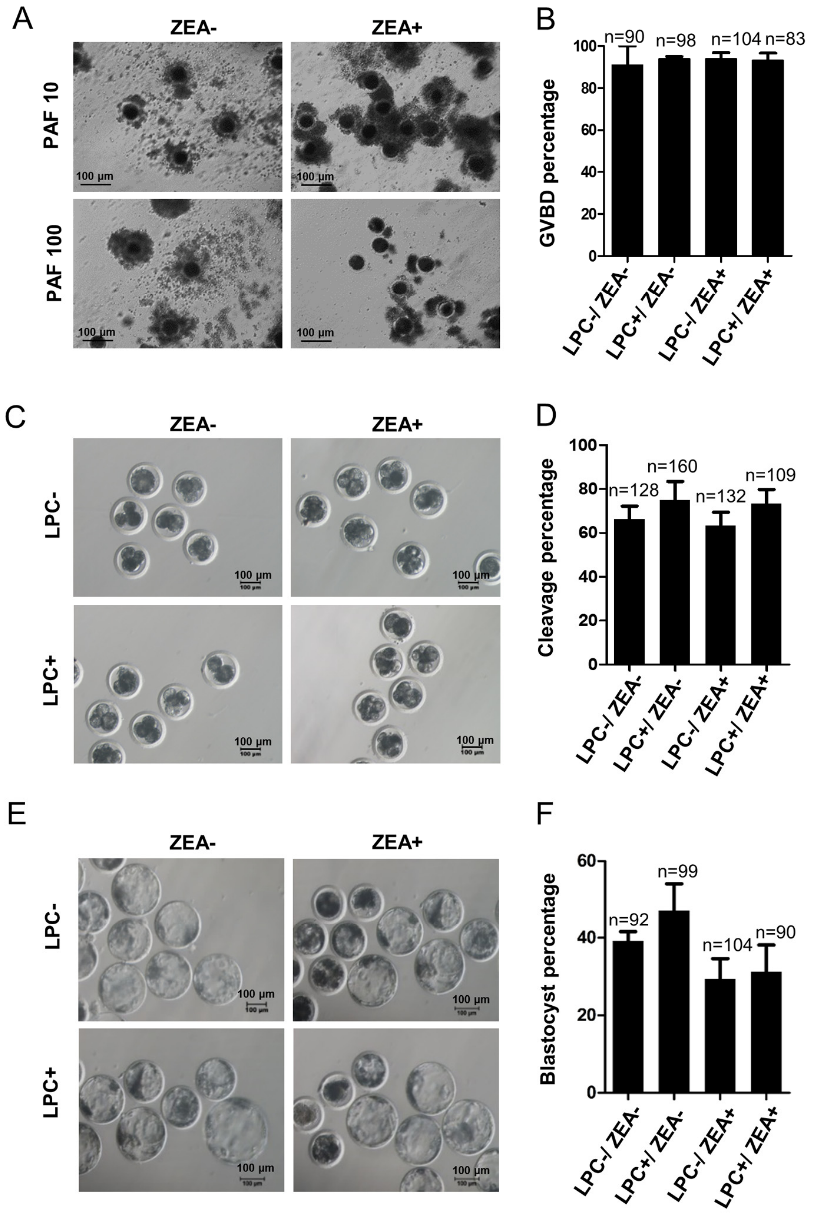

Figure S4. Morphology of CC expansion and the effect of LPC co-treated ZEA on the development of parthenogenetic oocytes. (**A**) Morphology of CC expansion status in 10 µg/mL or 100 µg/mL PAF and 10 µM ZEA co-treated conditions. (**B**) The germinal vesicle breakdown (GVBD) percentage of oocytes in 10 µg/mL LPC and 10 µM ZEA co-treated condition. (**C**-**D**) The effect of supplementary 10 µg/mL LPC and 10 µM ZEA co-treated on cleavage of oocytes. (**E**-**F**) The effect of supplementary 10 µg/mL LPC and 10 µM ZEA on early parthenogenetic embryo development in vitro.

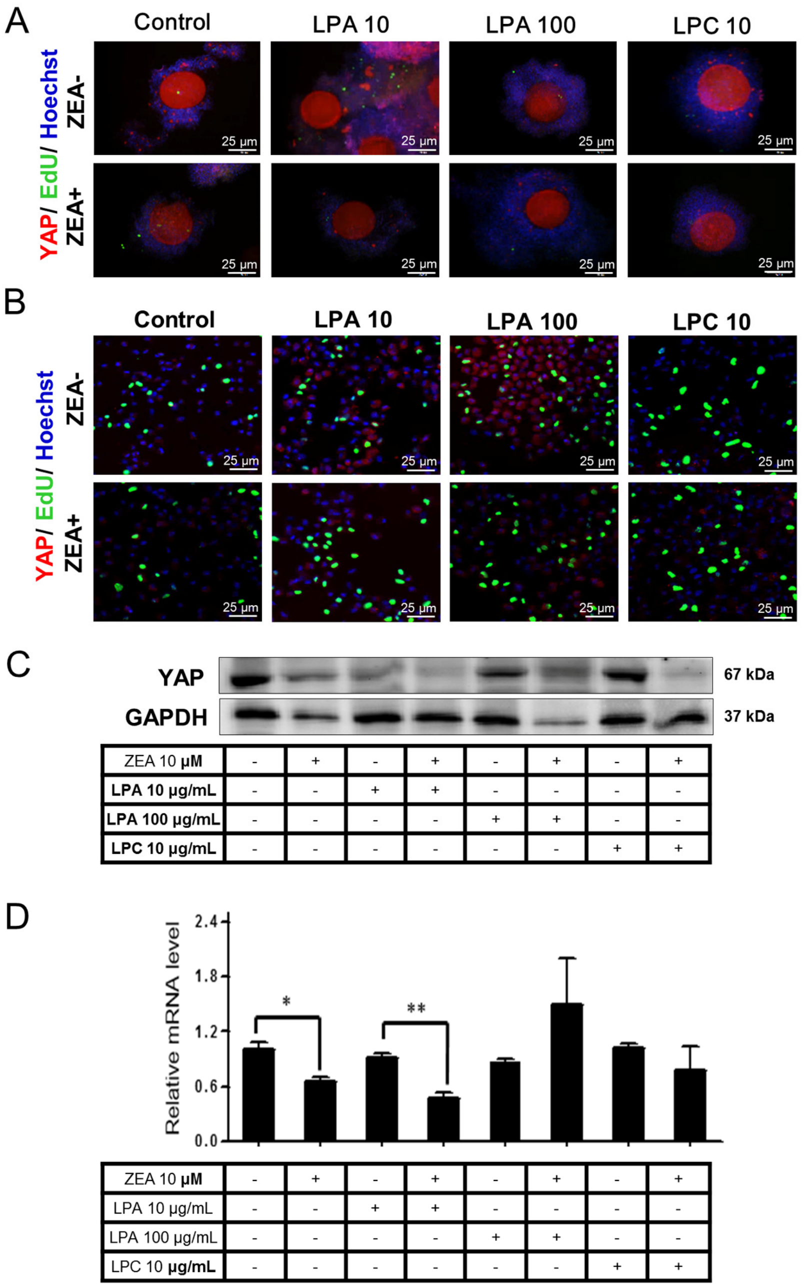

Figure S5. The effect of LPC or lysophosphatidic acid (LPA) co-treat with ZEA on the localization and expression of yes associated protein (YAP) protein. Immunofluorescence showed the cellular localization YAP and EdU in COCs (**A**) and GCs (**B**), which treated with 10 μg/mL LPA, 100 μg/mL LPA or 10 μg/mL LPC, and with or without 10 μM ZEA. Western blotting results showed the expression of YAP in GCs in LPC or LPA co-treated with ZEA (**C** and **D**).
