# Supplementary material for: Phosphatidylcholine could protect the defect of zearalenone exposure on follicular development and oocyte maturation
Source: Aging (Albany NY). 2018 Nov 25;10(11):3486–506. doi: 10.18632/aging.101660 (PMC6286824; doi:10.18632/aging.101660)
Supplement: Supplementary Tables 1-3 [file aging-10-101660-s002.pdf]

SUPPLEMENTARY TABLES

Table S1. Q-TOF coupled UPLC gradient elution.

Time (min)	A%(H_2O+1% formic acid)	B%(acetonitrile)
0	95	5
20	40	60
40	0	100
50	0	100
51	95	5
60	95	5

Table S2. Orbitrip coupled UPLC gradient elution.

Time (min)	A% (MeOH)	B% (H2O+1% formic acid)
0	5	95
20	60	40
40	95	5
50	95	5
51	5	95
60	5	95

Table S3. The primers used in RT-qPCR.

Genes	Accession number	Forward primer/Reverse primer	Fragment size (bp)
HAS2	GU990841.1	CAAACCGAGTGCTGAGTCTG<br>CACATCGCATTGTACAGCCA	151
CX43	NM_001244212.1	ACTGAGCCCCTCCAAAGACT<br>GCTCGGCACTGTAATTAGCC	191
PTX3	NM_001244783.1	TCAGTGCCTGCATTTGGGTC<br>CTACATGCCCTTGTTCAGAA	225
GAPDH	NM_001206359.1	TCGGAGTGAACGGATTTGGC<br>TGCCGTGGGTGGAATCATAC	147
ADAMTS1	DQ177331	CGTGAACAAGACCGACAAGA<br>AACTCCTCCACCACACGTTC	103

Please browse the Full Text version to see the data of Supplementary Tables:

Table S4. Differential content metabolites in the GC media of control and ZEA-treatment groups.

Table S5. Chemical prediction of the differential content metabolites in the GC media of control and ZEA-treatment groups.

Table S6. Top ten differential content metabolites in small and large follicle.

Table S7. Chemical predictions of the top ten differential content metabolites in small and large follicle isolated FF.

Table S8. Co-existing metabolites in each groups.

Table S9. Predictive structures of the co-existing metabolites.
