# Supplementary material for: Phosphatidylcholine could protect the defect of zearalenone exposure on follicular development and oocyte maturation
Source: Aging (Albany NY). 2018 Nov 25;10(11):3486–506. doi: 10.18632/aging.101660 (PMC6286824; doi:10.18632/aging.101660)
Supplement: Supplementary Table 4 [file aging-10-101660-s003.pdf]

Table S4. Differential content metabolites in the GCs culture media of control and ZEA treatment groups

Name	Fold change	Log2 |fold change|	P-value	Variation trend	Mass charge ratio (m/z)	retention time (min)	Mean value in control GCs media	Standard error in control GCs media	Mean value in ZEA supplied GCs media	Standard error in ZEA supplied GCs media
M342T20_1	193.515853	7.5963079	2.47E-10	UP	342.135181	19.529033	1585.827305	2462.681729	325922.2912	34730.2897
M320T20_1	289.123147	8.1755403	9.63E-10	UP	320.153615	19.5299	1254.756143	2040.685006	400707.2357	49276.7481
M341T20_1	248.170914	7.9551902	1.51E-09	UP	341.131248	19.528858	8020.153809	11616.12905	1990368.899	257386.251
M301T20_1	160.975847	7.3307004	2.29E-09	UP	301.140224	19.528033	5114.736118	4016.96129	823348.977	111143.363
M319T20_2	376.32141	8.5558216	2.90E-09	UP	319.150022	19.525125	6332.047234	9118.879244	2382884.94	330991.027
M761T37_1	191.750299	7.583085	4.31E-09	UP	760.577208	36.776775	968.580221	1866.429689	229745.8838	33517.1163
M659T20_1	1635.34193	10.675377	1.09E-08	UP	659.276469	19.528033	74.41380811	173.5296133	497119.3477	80206.1451
M783T37_1	13.6548079	3.7713371	1.99E-08	UP	782.558286	36.776667	15357.32772	20949.70509	211044.8238	45505.6401
M761T36_1	193.038966	7.5927483	2.25E-08	UP	760.576653	35.9955	780.3325912	1466.035803	188619.891	33073.2431
M783T36	14.4331223	3.8513115	7.01E-08	UP	782.558754	36.003217	11685.59574	16091.22525	171026.3665	39835.2144
M283T37_2	1.91940701	0.9406607	4.85E-07	UP	283.259337	36.776733	229405.7367	32796.22629	440322.978	65795.1995
M351T33_1	4.57775233	2.1946394	2.11E-06	UP	351.224616	32.993125	80749.42591	25629.17662	369650.8727	93435.7676
M292T1_1	1.16113898	0.2155407	3.13E-06	UP	291.63636	0.9567417	1249286.787	56568.36287	1450595.585	74170.909
M329T33_1	4.91273747	2.2965271	3.48E-06	UP	329.242851	33.00405	34586.76469	9518.155821	169915.6949	44921.2488
M761T38_1	141.553854	7.1452072	4.81E-06	UP	760.57685	37.927933	652.4709548	963.7836864	92359.77811	30088.9027
M308T1_8	1.13442379	0.1819597	4.91E-06	UP	308.150529	0.9551667	702712.9219	36714.43114	797174.257	21907.6097
M327T34	11.8798123	3.5704401	5.11E-06	UP	327.224803	33.564833	28287.74426	17981.26793	336053.0913	105037.701
M305T37	1.39130643	0.4764402	5.50E-06	UP	305.240976	36.77675	267414.8815	28638.11037	372056.0449	40826.6314
M305T34_1	11.8837021	3.5709124	8.15E-06	UP	305.243154	33.561917	15072.22088	9558.308589	179113.7822	58944.5088
M378T10_4	1.18656987	0.2467971	8.35E-06	UP	378.043687	9.9370583	535058.4378	39623.6109	634884.2221	17022.408
M257T36	2.09531801	1.0671692	1.01E-05	UP	257.244217	35.986425	190173.6107	35239.40539	398474.1919	84011.6624
M331T34	7.47214407	2.9015223	1.18E-05	UP	331.258585	34.295092	14864.95939	5784.146116	111073.1182	36095.7018
M353T34	7.07345287	2.8224146	1.40E-05	UP	353.240297	34.292367	28239.36285	11489.87723	199749.8023	65922.8194
M205T1_5	1.10163684	0.1396487	2.57E-05	UP	205.435266	0.9400333	1078519.723	39125.17777	1188137.06	47103.1793
M263T1_6	1.12105586	0.1648582	2.80E-05	UP	263.14287	0.94185	1322514.474	46776.08504	1482612.604	72521.113
M517T14_1	177.533374	7.4719464	5.20E-05	UP	517.162584	13.718	848.8906237	1305.839684	168173.5054	73726.8154
M250T17	1.87610516	0.9077407	7.97E-05	UP	250.174189	16.8993	88722.08489	33523.5627	166451.9614	34983.1647
M460T21	3.66875543	1.8752907	8.22E-05	UP	460.262924	20.885167	33500.0045	11137.02265	122903.3235	43454.5396
M205T5_3	1.23105691	0.2998975	0.00011	UP	205.094539	4.7426583	1298311.246	134969.6037	1598295.026	137619.786

M301T1_1	1.15165248	0.2037054	0.000114	UP	300.641475	0.9567417	689545.072	41987.09759	794116.2907	51789.9275
M761T40	96.3019501	6.5894931	0.000135	UP	760.577587	39.852233	607.6386777	1002.537757	71150.01783	35213.9322
M1070T18_2	2.89405342	1.5330916	0.000173	UP	1069.65854	18.17735	68758.5426	70280.45686	198990.8952	22978.1325
M784T32	13.3202575	3.7355501	0.000198	UP	783.56233	31.589383	4931.807441	6673.312571	65692.94499	32609.773
M783T32	13.012183	3.7017911	0.000208	UP	782.559017	31.597483	10484.12697	14087.74311	136421.3789	68077.3703
M301T36	1.46889902	0.5547352	0.00021	UP	301.20738	35.986475	140840.6687	36082.34675	206880.7206	24743.4183
M303T34_2	1.75637341	0.8125996	0.00022	UP	303.225399	33.968467	85425.14509	34597.96506	150038.4531	26682.7981
M761T33	116.174919	6.8601548	0.000223	UP	760.576004	32.97515	444.6213024	701.2608049	66894.07771	35496.0588
M512T14	133.835617	7.0643183	0.000254	UP	512.206851	13.718033	631.6707628	871.6807394	93318.55929	50401.2991
M377T10_3	1.14780162	0.1988733	0.000256	UP	377.040736	9.9368667	3128330.767	264196.2236	3590703.132	142703.671
M761T32	48.8509386	5.6103144	0.000279	UP	760.574408	31.664558	2442.956765	689.4969675	119340.7308	64371.713
M383T1_9	1.21464312	0.2805325	0.000393	UP	383.111134	1.2497417	940458.5681	119753.5568	1142321.528	32565.2546
M539T12_1	15.5872087	3.9622907	0.000459	UP	538.566314	12.072533	4165.647028	2257.028166	64930.80947	35939.9664
M1070T18_1	2.50293666	1.3236218	0.000761	UP	1069.53429	18.185633	72355.5603	70988.92583	181101.3847	27267.752
M1222T18_3	2.58385814	1.3695269	0.000927	UP	1222.03742	18.177333	45022.74306	47741.44371	116332.3811	21184.2757
M363T53_4	1.19878122	0.2615684	0.001082	UP	362.921094	52.802717	250270.6625	33961.77715	300019.7687	10981.5935
M1222T18_4	2.08076382	1.0571132	0.001101	UP	1222.17996	18.2447	72231.47836	51901.92661	150296.6469	33172.0337
M1070T18_3	2.13077883	1.0913809	0.001316	UP	1069.78472	18.2293	80794.60411	63342.90336	172155.4321	9118.77623
M761T38_2	132.296845	7.0476348	0.001693	UP	760.576864	38.370233	619.6601946	652.608573	81979.08859	58326.5962
M951T18_3	2.16622264	1.1151815	0.001739	UP	950.80871	18.19415	39445.61439	33567.08063	85447.98306	13184.5012
M301T20_2	19.3935835	4.2775075	0.001781	UP	301.139936	19.926425	3749.003928	528.1838655	72706.62087	49831.1795
M1222T18_5	1.99339015	0.9952241	0.002826	UP	1222.32362	18.269883	80016.64591	62324.97488	159504.3938	17150.0981
M1070T18_4	2.17716206	1.1224488	0.003012	UP	1069.90902	18.178725	59285.91717	55118.633	129075.0497	21774.0631
M1010T52_2	2.46512227	1.3016592	0.003049	UP	1009.73874	52.307208	30887.84411	16428.52899	76142.31224	35584.352
M761T39	131.060607	7.0340903	0.00321	UP	760.577729	39.341867	742.6492085	1335.225597	114525.0466	90427.1336
M432T19_1	2.48018765	1.3104493	0.003283	UP	432.232444	18.66515	29668.61733	16617.91292	73583.73831	34834.599
M731T10_4	1.09312191	0.1284543	0.003479	UP	731.092151	9.9200333	812814.6815	54909.86741	888505.5354	44661.2712
M308T1_1	1.09063697	0.125171	0.00357	UP	307.6494	0.9551667	3105187.27	184002.2881	3386632.03	191624.531
M951T18_4	2.16737737	1.1159504	0.003813	UP	950.920094	18.163083	40193.37669	38333.86363	87114.21489	15928.7165
M431T53_2	1.22905551	0.2975501	0.004393	UP	430.907859	52.802675	254503.5945	49010.02076	312799.0443	9996.97254
M743T12_7	1.18144992	0.2405585	0.004596	UP	743.393853	11.68125	364900.9055	54204.88016	431112.1464	30918.0089
M188T5_2	1.18722299	0.2475909	0.005364	UP	188.068276	4.7405583	2016515.019	226002.1775	2394052.991	297508.758
M281T7_1	1.33594604	0.4178617	0.005953	UP	281.091965	7.0417917	148696.0656	18533.05206	198649.9192	43721.2591
M319T20_1	65.9360384	6.0429953	0.005966	UP	319.148963	19.9084	2181.149144	366.5608047	143816.3337	125243.058

M424T10_4	31.7199092	4.9873167	0.006024	UP	424.16118	10.241125	1684.864204	241.8550653	53443.73957	45846.9403
M704T29	3.27944114	1.71345	0.007279	UP	703.567568	28.8985	18588.87697	16785.96896	61176.23213	38454.7603
M600T1_5	1.28325604	0.3598091	0.007538	UP	600.275719	1.0062917	268880.4904	18975.43242	345042.5137	70192.0807
M218T7_3	5.43245787	2.4416051	0.008537	UP	218.076117	6.7235333	23456.45977	12212.46878	127426.2295	98193.5021
M166T3_3	1.12216776	0.1662884	0.009196	UP	166.084428	2.87385	3700968.946	312418.3611	4153108.027	375156.508
M575T12_12	10.1702491	3.3462831	0.010059	UP	575.270771	12.014133	5150.290855	1077.705115	52379.74085	46009.0944
M1070T18_5	1.77165813	0.8251002	0.014874	UP	1070.03649	18.197083	44715.02574	32905.92042	79219.7389	22718.3064
M356T10_3	1.07839708	0.1088885	0.014993	UP	356.062181	9.92	1477144.343	103137.0008	1592948.152	88413.3419
M178T7	6.5777806	2.7176009	0.015156	UP	178.084609	6.7373833	5258.324095	2403.822376	34588.10221	30990.8421
M339T41_2	4.30870922	2.1072557	0.015418	UP	339.34115	41.081817	13772.47067	6710.634907	59341.57136	48276.1901
M1223T18_1	1.76660031	0.8209757	0.015589	UP	1222.60992	18.211067	52939.1903	40874.61086	93522.38998	22424.4369
M124T41	1.70423356	0.7691231	0.016658	UP	124.086039	41.325208	107898.491	52014.92986	183884.2291	73471.3269
M744T12_3	1.22136592	0.2884955	0.017411	UP	743.895674	11.6858	262136.4688	39689.65502	320164.5494	56752.2846
M338T41_2	2.28532644	1.1924003	0.017758	UP	338.337707	40.644583	1423512.388	925331.6994	3253190.497	1920775.56
M1069T18_4	1.90024909	0.9261885	0.017773	UP	1069.40838	18.23625	64204.50607	47100.05521	122004.554	51789.9798
M312T10	1.30739273	0.3866926	0.018136	UP	312.122785	9.8017917	127322.8119	30197.68043	166460.9188	36599.6614
M124T42	1.81068498	0.8565356	0.018431	UP	124.086015	41.720817	73153.66483	17909.91843	132458.242	64724.8345
M657T10_4	1.129605	0.1758184	0.020738	UP	657.358661	9.5501417	334782.2655	44940.77074	378171.7223	28431.2427
M340T41_1	1.9087002	0.9325905	0.023843	UP	340.343724	40.644583	36632.69554	21081.09405	69920.83324	36002.6454
M615T1_9	1.43736309	0.5234245	0.024694	UP	615.294128	0.9920833	96458.96418	7481.488184	138646.5546	49408.1459
M697T9_3	1.11846871	0.1615249	0.025613	UP	697.352731	9.4308	277143.4168	31437.95583	309976.2408	28807.4577
M339T41_1	1.99866789	0.9990388	0.026741	UP	339.340774	40.644583	308524.8797	200352.9071	616638.7692	340949.849
M288T53	1.34076553	0.423057	0.032009	UP	287.884894	53.357517	150569.0337	44890.19526	201877.7705	53240.4591
M167T3_2	1.09887345	0.1360252	0.033056	UP	167.087501	2.87385	351682.4764	26864.6403	386454.5347	38746.5908
M310T10_9	1.27949781	0.3555777	0.03385	UP	310.126096	9.8018167	4399801.063	1201062.199	5629535.806	1193399.57
M1098T51	1.8742888	0.9063433	0.044984	UP	1097.7894	51.053717	31244.50471	24598.89474	58561.22528	31439.6096
M516T12_7	2.74869488	-1.4587468	2.07E-10	DOWN	516.28226	11.817058	813307.1042	87255.15585	295888.4639	94232.0939
M675T11_3	1.44020236	-0.5262715	8.13E-10	DOWN	675.379941	11.298917	733947.6351	45145.52368	509614.2413	39835.0775
M487T12_6	2.89831455	-1.5352142	1.30E-09	DOWN	487.266405	11.568	461469.8997	62201.91921	159220.0889	56350.5667
M502T12_3	3.19801122	-1.677175	1.37E-09	DOWN	501.941549	11.685617	559555.5651	54984.33024	174969.8567	80891.0407
M487T12_4	2.96539069	-1.5682222	1.97E-09	DOWN	486.932028	11.568017	652136.8811	86555.43588	219916.0074	88768.7923
M494T12_8	2.11394305	-1.0799365	1.97E-09	DOWN	494.27991	11.678292	293863.136	24082.94993	139011.8507	33591.7363
M609T11_4	1.58942663	-0.6685064	2.19E-09	DOWN	609.34086	10.861375	612547.4205	47021.32824	385388.9262	45741.3873
M502T12_6	2.83362256	-1.5026476	2.82E-09	DOWN	502.275791	11.685558	249007.2836	21628.72583	87875.95328	35009.7444

M517T12_2	2.92322505	-1.5475609	3.89E-09	DOWN	516.616881	11.7979	604831.3958	58481.01775	206905.519	89485.2303
M472T11_8	2.88336901	-1.5277555	5.94E-09	DOWN	472.256753	11.4336	493201.8273	74631.53111	171050.5405	62161.2399
M524T12_2	2.1651129	-1.1144423	6.09E-09	DOWN	523.630122	11.9212	280199.7741	15633.32691	129415.7797	32293.3008
M675T11_2	1.43710263	-0.5231631	6.76E-09	DOWN	674.878075	11.298808	1152350.415	69477.71277	801856.7285	81512.0429
M547T12_13	2.2562844	-1.1739489	8.49E-09	DOWN	547.285205	12.0389	380814.7174	44605.67737	168779.5727	49220.7008
M517T12_6	2.88875017	-1.5304454	9.18E-09	DOWN	516.951124	11.7931	272107.0599	22526.10658	94195.42847	40983.742
M587T11_1	1.59352572	-0.6722223	1.09E-08	DOWN	586.826705	10.695033	861367.9673	75359.62166	540542.2448	69217.6816
M546T12_6	2.97095199	-1.5709253	1.10E-08	DOWN	545.967367	11.934558	518109.8422	80800.28648	174391.8593	74171.6469
M473T11_2	2.87677837	-1.5244541	1.14E-08	DOWN	472.590985	11.433517	324571.3951	50607.99194	112824.6089	44371.4111
M609T11_2	1.58445492	-0.6639866	1.51E-08	DOWN	608.839226	10.86135	1056535.311	93746.38262	666813.109	85508.4888
M546T12_3	2.39934323	-1.2626396	1.54E-08	DOWN	545.632927	11.944592	689099.0626	60823.88491	287203.2042	98869.8492
M465T11_5	2.3713698	-1.2457207	1.58E-08	DOWN	464.929332	11.436033	224802.4855	32272.126	94798.57821	26271.0145
M575T12_9	2.19336224	-1.1331441	1.79E-08	DOWN	574.983428	12.152733	431943.9741	32343.43146	196932.3472	57392.141
M561T12_4	2.24266267	-1.1652126	2.71E-08	DOWN	560.642434	11.93505	310412.0113	27890.64435	138412.261	44305.0776
M562T12_8	1.69247471	-0.7591343	3.02E-08	DOWN	561.960131	12.139817	294323.4582	27463.56108	173901.2449	30452.9888
M480T12_1	2.23345823	-1.1592793	5.02E-08	DOWN	479.605008	11.566533	271098.7558	33625.82812	121380.7145	39946.0964
M489T12_3	2.82093545	-1.4961737	5.06E-08	DOWN	488.918536	11.55525	316883.581	44321.43997	112332.8014	55094.4752
M509T12_6	2.20844037	-1.1430279	7.89E-08	DOWN	508.954994	11.7979	295944.5768	25096.86042	134006.1431	44461.7871
M548T12_3	1.96718588	-0.9761333	8.81E-08	DOWN	547.61924	12.031117	289758.1555	27135.58646	147295.7683	40307.8387
M504T12_2	2.7751867	-1.4725848	9.12E-08	DOWN	503.594129	11.681717	346004.0839	62368.31162	124677.7682	48860.7905
M502T12_1	2.63359337	-1.3970326	1.03E-07	DOWN	501.607191	11.68575	773421.2032	69862.00287	293675.2548	132744.57
M529T12_10	2.40894178	-1.2683995	1.04E-07	DOWN	529.305798	11.921267	817226.549	124580.9384	339247.1145	51455.3994
M515T12_1	2.54597899	-1.3482205	1.25E-07	DOWN	514.63057	11.803508	764333.6573	129474.4805	300212.0842	65740.8756
M612T51_1	3.09544805	-1.6301482	1.33E-07	DOWN	612.17541	51.095683	738650.9216	145835.4488	238624.881	97302.2588
M565T11_5	1.59255348	-0.6713418	1.46E-07	DOWN	565.314811	10.524967	332742.5713	35051.13042	208936.514	31116.2691
M486T12_3	2.78895394	-1.4797241	1.50E-07	DOWN	485.948921	11.565767	163312.8712	30161.52895	58557.0342	16662.1441
M458T11_1	2.93346022	-1.5526034	1.56E-07	DOWN	457.581346	11.298225	307928.1906	59352.10717	104970.9787	46875.4936
M587T11_4	1.59599822	-0.674459	1.56E-07	DOWN	587.328065	10.7095	477442.6156	52565.77339	299149.8428	38625.2215
M719T12_3	1.25506521	-0.3277623	1.92E-07	DOWN	718.903114	11.558033	820505.7277	37265.54249	653755.4555	49845.4516
M503T12_5	2.63201108	-1.3961656	2.06E-07	DOWN	503.259693	11.685833	483481.0384	86729.0922	183692.6305	77529.8614
M653T11_2	1.39373161	-0.4789528	2.24E-07	DOWN	652.864962	11.147675	1163501.843	92894.014	834810.5421	89522.7472
M501T12_2	2.46777137	-1.3032087	2.35E-07	DOWN	500.624192	11.6858	217161.0429	38591.59648	87998.85022	31760.4826
M697T11_2	1.326002	-0.407083	2.40E-07	DOWN	696.890458	11.432458	1003090.103	52784.11545	756477.0667	75218.9209
M515T12_3	2.42501433	-1.2779933	2.42E-07	DOWN	515.29917	11.800425	259697.0071	45993.46876	107090.9166	26521.4555

M548T12_9	2.89915188	-1.5356309	2.67E-07	DOWN	547.953912	12.022133	152848.1865	30613.6592	52721.68997	18594.5561
M458T11_5	2.99020324	-1.5802435	2.92E-07	DOWN	457.91574	11.2987	207906.1054	42816.25578	69529.08848	30348.2938
M500T12_3	2.77898093	-1.4745559	3.21E-07	DOWN	499.955349	11.685575	664065.2109	125973.0293	238959.974	58188.2066
M474T11_6	2.73918994	-1.4537493	3.26E-07	DOWN	474.243321	11.431	260318.9891	47486.16204	95035.02668	46772.2113
M565T11_1	1.5526573	-0.6347394	4.38E-07	DOWN	564.813765	10.524292	603291.4815	67811.59587	388554.1788	52957.9237
M509T12_11	2.59079917	-1.3733972	4.42E-07	DOWN	509.289613	11.800958	245554.0796	47746.32527	94779.27993	27613.899
M486T12_1	2.89264145	-1.5323875	4.46E-07	DOWN	485.614462	11.568017	363815.2066	70272.21558	125772.6591	27802.7128
M500T12_4	2.86110272	-1.5165713	5.54E-07	DOWN	500.2897	11.685833	501760.6268	99709.57516	175373.1609	42121.9088
M450T11_10	2.43835708	-1.2859094	5.72E-07	DOWN	450.254647	11.3144	164014.6694	31470.55504	67264.41773	22622.1305
M518T12_6	2.49708391	-1.3202443	5.99E-07	DOWN	517.934752	11.795225	455739.49	46298.5786	182508.6808	87656.8914
M446T10_1	1.51105955	-0.5955605	6.32E-07	DOWN	445.744382	10.474058	343593.5365	33477.6061	227385.8349	35833.964
M518T12_9	2.5858144	-1.3706187	6.97E-07	DOWN	518.269262	11.803467	326274.5332	59297.49762	126178.636	61154.2809
M471T11_1	3.07724541	-1.6216395	7.32E-07	DOWN	470.604948	11.433583	344226.3792	73294.25653	111861.8546	31927.6876
M559T12_8	2.32113616	-1.2148312	7.35E-07	DOWN	558.990469	12.0371	437775.576	82800.13768	188604.0052	57476.6612
M515T12_2	2.39664864	-1.2610184	7.91E-07	DOWN	514.965048	11.79845	592330.8868	111956.2376	247149.6558	94515.1597
M460T11_1	2.97113515	-1.5710142	8.57E-07	DOWN	459.56771	11.298975	198277.0097	44072.87482	66734.42964	25998.3952
M575T12_13	1.85531461	-0.8916638	9.10E-07	DOWN	575.317574	12.052775	252732.3978	29609.65607	136220.7773	39036.2972
M561T12_10	2.23704766	-1.161596	9.70E-07	DOWN	560.976825	12.04045	210844.4608	36230.58851	94251.21556	35697.8567
M610T51	2.87732819	-1.5247298	9.71E-07	DOWN	610.176386	51.08745	2026574.852	445917.8228	704325.2351	252317.367
M543T10_1	1.64763474	-0.7203965	1.05E-06	DOWN	542.801212	10.35325	404874.174	40355.25781	245730.5392	54001.3895
M471T11_4	3.22120307	-1.6875996	1.39E-06	DOWN	470.93914	11.433583	237600.9096	53140.69918	73761.54318	19417.3058
M564T9_2	1.33307023	-0.4147528	1.43E-06	DOWN	564.352168	8.8873	504503.778	40994.76485	378452.5129	38938.8429
M608T9_3	1.33183874	-0.4134194	1.63E-06	DOWN	608.377799	9.2309667	509960.217	42355.51836	382899.3727	38785.8552
M489T12_1	3.05770813	-1.6124507	1.88E-06	DOWN	488.584519	11.5642	454714.1052	109607.7417	148710.762	65855.3243
M520T9_3	1.34559203	-0.4282411	2.15E-06	DOWN	520.326345	8.5074333	424396.5903	33807.49344	315397.6708	37208.9652
M404T10_2	1.49225103	-0.5774902	2.20E-06	DOWN	403.734369	10.357233	336468.1101	28203.89515	225476.8829	40184.6007
M474T11_5	3.09994523	-1.6322427	2.37E-06	DOWN	473.908603	11.430833	391269.0046	96597.72092	126218.0381	55754.9306
M588T12_8	1.57204641	-0.6526438	2.41E-06	DOWN	588.00626	12.1502	241624.3238	32166.88247	153700.5029	19539.3354
M456T11_6	3.11681787	-1.6400739	2.51E-06	DOWN	456.26409	11.303433	133745.4046	32025.87833	42910.88232	13617.9057
M611T51	2.82236736	-1.4969058	2.93E-06	DOWN	611.176757	51.087517	1037065.219	249114.2221	367445.1572	153171.207
M445T11_1	2.68116614	-1.4228606	3.73E-06	DOWN	444.557924	11.161958	221277.0355	52688.18008	82530.14697	33509.1077
M426T11_1	1.41386495	-0.4996443	4.05E-06	DOWN	425.747363	10.591775	299682.0507	29512.71612	211959.4603	30715.632
M531T12_12	2.4912934	-1.3168949	5.04E-06	DOWN	531.292168	11.921217	606404.396	60551.68777	243409.4663	137583.495
M473T11_7	2.57353915	-1.3637537	5.09E-06	DOWN	472.9255	11.433517	146382.3818	31630.46557	56879.79598	30968.4349

M519T12_2	2.82308723	-1.4972737	5.31E-06	DOWN	518.60323	11.803567	166200.8966	42011.37159	58872.03731	26331.6685
M590T12_3	1.90997919	-0.9335569	5.78E-06	DOWN	589.658441	12.149983	237116.8399	17044.80419	124146.2948	42802.9149
M613T51	2.83509854	-1.5033989	5.84E-06	DOWN	613.174942	51.1042	273701.709	69012.90858	96540.45728	33982.4806
M540T10	2.03480675	-1.0248918	6.03E-06	DOWN	540.323509	10.356167	191118.6689	36979.02879	93924.72722	14709.052
M421T10_5	1.9172224	-0.9390177	6.44E-06	DOWN	421.253972	10.239475	180955.9967	33005.45258	94384.45768	12705.6705
M521T10_1	1.59633635	-0.6747647	7.38E-06	DOWN	520.787494	10.168142	229878.2663	33835.4395	144003.6534	26116.203
M443T11_3	2.77424577	-1.4720956	7.71E-06	DOWN	442.905901	11.1644	179104.9274	46195.95061	64559.86328	30670.8152
M673T11_1	1.64609788	-0.7190501	7.96E-06	DOWN	672.902072	11.298167	461525.1997	73386.45649	280375.3087	47072.8449
M530T12_5	3.26772788	-1.7082878	8.60E-06	DOWN	529.974411	11.81845	235059.6359	65127.37946	71933.66305	28661.5216
M533T12_3	2.22077811	-1.1510653	8.73E-06	DOWN	532.944199	11.921217	303097.0785	66810.00728	136482.3786	50615.7959
M650T11_3	1.73435521	-0.7943994	1.09E-05	DOWN	650.3874	11.1484	700828.1545	118242.7937	404085.708	42160.5709
M424T10_1	1.49835008	-0.5833747	1.10E-05	DOWN	423.731584	10.239542	342016.5671	42476.50375	228262.1216	42125.3023
M584T11_3	1.9017028	-0.9272918	1.13E-05	DOWN	584.348993	10.696058	436945.0102	81227.18108	229765.1399	24415.1009
M445T11_3	2.63046225	-1.3953163	1.37E-05	DOWN	444.89263	11.149742	143498.2253	37870.75248	54552.47464	21675.7568
M620T11_5	1.34916439	-0.4320661	1.41E-05	DOWN	619.861263	11.013025	317927.9889	20024.48937	235648.0736	35124.5003
M533T12_1	2.35412436	-1.2351905	1.46E-05	DOWN	532.609574	11.820267	382771.9237	94278.22713	162596.3052	54044.368
M684T46	3.62262677	-1.8570362	1.49E-05	DOWN	684.19492	46.076483	1566620.912	485296.5858	432454.4068	313835.974
M493T12_2	2.21100797	-1.1447042	1.55E-05	DOWN	492.628464	11.6891	154901.5527	36287.94892	70059.24666	24564.8
M631T11_1	1.46532803	-0.5512237	1.66E-05	DOWN	630.851923	11.01305	1190732.333	113746.0859	812604.6236	162030.781
M443T10_3	1.79868768	-0.8469447	1.67E-05	DOWN	443.266714	10.474058	187814.917	35096.22658	104417.7479	13599.7082
M543T10_4	1.69828715	-0.7640804	1.79E-05	DOWN	543.302337	10.348508	209742.3299	21875.11073	123502.2769	37639.7581
M459T11_10	2.78557005	-1.4779726	1.96E-05	DOWN	459.233651	11.299	319705.0386	89673.16925	114771.8538	44479.5402
M485T12_5	3.06327791	-1.6150763	2.16E-05	DOWN	485.27982	11.568042	543221.8244	152347.0491	177333.51	40229.952
M562T11_3	1.88192524	-0.9122093	2.24E-05	DOWN	562.336192	10.529517	299812.029	60308.74451	159311.3384	20993.9886
M585T11_1	1.85441583	-0.8909648	2.25E-05	DOWN	584.851141	10.693867	246869.9379	47579.89821	133125.4481	12554.3766
M530T12_2	2.80403631	-1.487505	2.64E-05	DOWN	529.640102	11.819933	535190.8411	155551.9572	190864.4478	80861.4232
M664T11_2	1.42987306	-0.5158871	2.93E-05	DOWN	663.886676	11.297958	336318.8051	43628.21187	235208.8553	37069.0496
M382T10_1	1.51496915	-0.5992884	2.97E-05	DOWN	381.721516	10.118083	278126.1394	33323.93459	183585.3484	41540.3081
M672T11_2	1.67268172	-0.742163	3.17E-05	DOWN	672.39988	11.2987	691845.6566	123565.2666	413614.6457	41262.9974
M604T12_8	1.72940558	-0.7902762	3.19E-05	DOWN	604.334898	12.440667	180713.1998	10669.97435	104494.4011	33713.1326
M524T12_4	1.92745466	-0.9466969	3.24E-05	DOWN	523.964641	11.912067	208498.0172	14359.10399	108172.7222	44519.3325
M631T11_5	1.43750824	-0.5235702	3.38E-05	DOWN	631.35343	11.01305	700331.3678	54402.62076	487184.2459	98748.8324
M563T11_1	1.85835945	-0.8940296	3.59E-05	DOWN	562.837641	10.535717	164180.1745	34043.38443	88346.83437	10973.4994
M629T11_2	1.75850837	-0.8143522	3.98E-05	DOWN	628.87593	11.013067	403738.0537	76999.46318	229591.204	17510.5339

M537T12_3	2.38990891	-1.2569556	3.98E-05	DOWN	536.65362	11.933025	138285.6487	37486.7221	57862.30932	16405.6767
M607T11_1	1.79220528	-0.8417359	4.17E-05	DOWN	606.862888	10.85705	323305.1318	63756.62684	180395.1458	15269.114
M402T10_1	1.49481378	-0.5799658	4.30E-05	DOWN	401.718855	9.987475	215739.4071	32050.97208	144325.2736	26757.8865
M628T11_2	1.76599106	-0.820478	4.43E-05	DOWN	628.374495	11.013067	645677.7398	127555.6581	365617.7856	36667.5215
M560T12_7	2.03112584	-1.0222796	4.82E-05	DOWN	560.307814	11.93505	376591.4173	33135.5165	185410.1846	89737.4274
M545T12_2	1.62664243	-0.7018972	4.92E-05	DOWN	544.649388	12.023858	262667.9904	40777.61255	161478.6294	44416.4833
M651T11_1	1.70283376	-0.7679376	5.23E-05	DOWN	650.889617	11.16415	436968.2694	83256.93656	256612.4063	22125.5652
M499T12_1	2.91037289	-1.541204	5.62E-05	DOWN	498.637966	11.685783	147399.5485	44665.78795	50646.27599	10423.353
M391T41	1.30865615	-0.3880861	5.77E-05	DOWN	391.279633	40.661425	1300548.157	135730.7202	993804.339	126511.585
M694T11_2	1.58354407	-0.663157	5.90E-05	DOWN	694.412836	11.431225	623554.3801	105063.2163	393771.4092	90355.3024
M553T12_8	1.8172731	-0.8617752	6.11E-05	DOWN	552.980616	12.13975	207789.5466	45229.66411	114341.3977	17684.4576
M606T11_4	1.79862106	-0.8468913	6.14E-05	DOWN	606.361318	10.859175	549985	114493.9475	305781.4752	28901.156
M544T12_6	1.89251554	-0.9203051	6.44E-05	DOWN	543.980777	11.829983	415988.1984	71726.84361	219807.0188	93414.3039
M478T12_5	2.97919647	-1.5749233	6.70E-05	DOWN	478.287817	11.56805	96655.89482	30275.02737	32443.61221	7055.3349
M465T11_9	1.68019754	-0.7486309	6.76E-05	DOWN	465.279802	10.679492	162424.2274	31766.72759	96669.72112	10429.1694
M676T11_2	1.46453292	-0.5504406	8.46E-05	DOWN	675.881402	11.297958	307091.4832	49231.33647	209685.6126	21779.3024
M531T12_7	2.03748012	-1.026786	8.67E-05	DOWN	530.957555	11.720958	454144.4923	36595.944	222895.1771	114758.154
M498T12_4	2.84800281	-1.5099506	8.83E-05	DOWN	498.303599	11.685833	205636.0645	65676.22389	72203.60315	17931.3228
M695T11_2	1.40658371	-0.4921954	9.46E-05	DOWN	694.914597	11.433558	432612.2865	55387.15022	307562.4169	56631.8273
M469T11_3	3.75817815	-1.9100335	0.000104	DOWN	468.952807	11.43355	88047.27615	32071.92769	23428.18052	7171.10369
M588T12_11	1.64112388	-0.7146841	0.000115	DOWN	588.340798	12.243508	259412.0521	40534.06061	158069.7569	50346.6915
M513T12_5	2.40914162	-1.2685192	0.000121	DOWN	513.312983	11.803558	189459.8649	55175.64894	78642.06224	7755.35239
M573T12_11	2.04242217	-1.0302811	0.000132	DOWN	573.331132	12.13945	384624.8776	103729.5299	188318.0091	41375.6127
M544T12_8	2.14656745	-1.1020315	0.000162	DOWN	544.315377	12.021983	596027.526	166523.6816	277665.4072	33822.8948
M653T11_5	1.34323426	-0.4257109	0.000163	DOWN	653.366655	11.155592	725282.3176	43681.71533	539952.2199	100671.973
M456T11_4	3.32711295	-1.7342708	0.000164	DOWN	455.929516	11.300217	212438.5691	78556.34094	63850.72363	19864.743
M377T10_2	1.5370607	-0.6201741	0.000177	DOWN	377.040548	9.50195	183444.7212	8140.732323	119347.7404	34126.5586
M543T12_5	1.86476365	-0.8989928	0.000177	DOWN	542.663476	12.022083	219856.1943	55429.79022	117900.3001	19850.6574
M642T11_2	1.35438677	-0.4376398	0.000182	DOWN	641.874547	11.147717	333447.6879	14879.28674	246198.2767	47270.4258
M513T12_2	2.30096597	-1.2022396	0.000192	DOWN	512.978894	11.804008	249110.4813	76822.29268	108263.4359	24295.3052
M511T12_4	1.46341469	-0.5493386	0.000225	DOWN	510.962779	11.923667	285874.3674	50894.2422	195347.4773	28674.7165
M451T11_2	2.5902439	-1.373088	0.000258	DOWN	450.588487	11.298967	118038.9311	41025.94541	45570.58544	13226.5488
M697T11_4	1.38953148	-0.4745985	0.000299	DOWN	697.392132	11.431225	685867.4522	111850.1201	493596.195	45427.9591
M686T11_2	1.45440834	-0.5404324	0.000299	DOWN	685.899864	11.433533	307183.5573	55714.07298	211208.6062	30973.6543

M559T12_12	1.917086	-0.9389151	0.000316	DOWN	559.324854	12.129883	253808.9279	69606.61489	132393.0842	16249.2761
M484T12_1	3.11117379	-1.637459	0.000323	DOWN	483.628332	11.568108	151204.1539	58508.69836	48600.35598	10546.5305
M568T12_4	1.66743249	-0.7376284	0.000323	DOWN	567.655783	12.235458	157081.7895	36921.86516	94205.78663	19195.8639
M537T12_8	1.92176452	-0.9424316	0.000361	DOWN	536.988009	12.026375	127291.6221	35831.39936	66236.84664	22308.4894
M716T12_5	1.36002593	-0.4436342	0.000364	DOWN	716.425397	11.5642	533578.2398	83636.60705	392329.4604	47932.2907
M528T12_3	2.02699482	-1.0193424	0.000396	DOWN	527.654015	11.910133	275922.3163	82550.85879	136123.8392	19084.2969
M329T10_3	1.39409628	-0.4793302	0.000404	DOWN	329.18291	9.5501917	236290.6541	37018.45498	169493.784	31339.2
M511T12_2	1.47652366	-0.5622045	0.000438	DOWN	510.627588	11.921275	372530.3238	73268.95571	252302.3051	35624.8246
M528T12_6	1.97437408	-0.9813954	0.000471	DOWN	527.988195	11.9212	214142.1906	64052.97368	108460.799	16247.9434
M496T24_2	1.47605035	-0.5617419	0.000473	DOWN	496.333236	23.558625	287033.1843	56735.60703	194460.2938	19907.7984
M632T11_2	1.35601916	-0.4393776	0.000543	DOWN	631.855001	11.013067	266551.2441	21930.55433	196568.9365	43709.7089
M698T11_3	1.28668408	-0.3636579	0.000571	DOWN	697.893968	11.433517	285735.101	34167.0086	222070.9081	34049.1103
M557T12_7	1.71475941	-0.7780062	0.000686	DOWN	557.00444	12.129883	237496.8842	63789.36887	138501.5777	25507.9542
M525T28	1.3736175	-0.4579803	0.000735	DOWN	525.367459	28.13325	330489.2575	57504.69932	240597.7334	34189.4899
M673T11_2	1.75703274	-0.8131411	0.000769	DOWN	673.403275	11.298225	198642.1601	55199.5859	113055.469	11678.3992
M528T12_10	1.83320907	-0.8743713	0.000817	DOWN	528.322475	11.921167	103284.1963	30884.73035	56340.65312	14533.5248
M542T12_12	1.72386359	-0.7856456	0.000848	DOWN	542.328935	12.021983	267810.2903	74311.68722	155354.6881	35354.2624
M717T12_2	1.5277725	-0.6114297	0.000864	DOWN	716.927605	11.567267	415316.6601	94877.86091	271844.5717	29135.794
M654T35	2.06199431	-1.0440404	0.00099	DOWN	654.323052	35.044725	88398.79863	30433.33901	42870.53464	16673.1261
M652T10	1.21256722	-0.2780647	0.000992	DOWN	652.403339	9.5501917	415626.1445	35740.80927	342765.4459	45749.9099
M686T46	3.52431407	-1.8173425	0.001219	DOWN	686.193387	46.0846	679810.4979	338213.8736	192891.577	140336.371
M467T11_1	3.56258515	-1.8329245	0.001439	DOWN	466.582824	11.225033	63509.51921	32196.09715	17826.80737	6009.13422
M589T11_3	1.2130563	-0.2786465	0.001529	DOWN	589.304735	10.710142	712711.6341	84241.30052	587533.8472	61713.9749
M557T12_10	1.69119489	-0.7580429	0.001559	DOWN	557.33867	12.13975	196518.3312	57723.72439	116200.8782	19136.8089
M685T46	3.24163044	-1.6967196	0.001688	DOWN	685.195303	46.07735	1037065.015	521327.8001	319920.8033	192817.488
M572T12_11	1.60622244	-0.6836717	0.00174	DOWN	572.013898	12.050283	113180.8352	31175.81538	70463.98577	9846.59315
M499T12_3	2.84254436	-1.5071829	0.001973	DOWN	498.972116	11.685833	77858.52297	37351.45979	27390.43377	7761.79483
M517T12_10	3.82741798	-1.9363715	0.002094	DOWN	517.285293	11.6005	66992.98588	37055.17791	17503.44131	9698.3892
M805T41	1.19415406	-0.255989	0.002251	DOWN	804.537539	40.6614	1160793.886	124584.2149	972063.7594	111899.095
M804T41_1	1.20525726	-0.2693411	0.002261	DOWN	803.533776	40.661458	2539102.233	246311.1243	2106689.021	293013.38
M373T10_5	1.25176011	-0.3239581	0.002286	DOWN	373.208288	10.117	241992.9763	24585.19596	193322.1672	34764.3731
M688T46	3.82020004	-1.9336482	0.002362	DOWN	688.191449	46.07735	100544.8244	56324.73404	26319.25641	8420.00478
M738T12_6	1.25465625	-0.3272921	0.002434	DOWN	738.438495	11.683558	419783.2533	61514.93543	334580.2921	43041.1236
M481T12_6	1.15705522	-0.2104577	0.003053	DOWN	481.277665	11.690983	321044.9782	31081.61413	277467.2923	25233.9652

M497T24_1	1.42780764	-0.5138016	0.003059	DOWN	497.336717	24.164383	481908.2957	114621.2099	337516.2599	31736.2787
M496T24_1	1.47642846	-0.5621115	0.003125	DOWN	496.333625	24.16435	2024389.083	519632.0884	1371139.3	189287.939
M574T12_6	1.52895821	-0.612549	0.003408	DOWN	573.999949	12.052283	133944.6304	19610.3908	87605.16111	36591.5609
M524T28_2	1.3172016	-0.3974762	0.003412	DOWN	524.364233	28.124725	1185410.291	228857.4887	899945.9882	100015.446
M695T11_3	1.55822659	-0.639905	0.003431	DOWN	695.416053	11.432458	188727.9532	54604.44123	121117.1434	12153.4272
M572T12_5	1.48366109	-0.5691616	0.003945	DOWN	571.679384	12.24065	184169.1504	47719.26875	124131.5501	28592.7289
M318T9	1.40377467	-0.4893114	0.00458	DOWN	318.160856	9.2320833	169831.7264	32236.07459	120982.1849	35132.925
M490T7_3	1.17457138	-0.2321344	0.004676	DOWN	490.124914	6.838125	245915.3904	30876.71571	209366.0665	12203.6303
M546T28_1	1.3290056	-0.4103472	0.004907	DOWN	546.345736	28.133	468358.3685	99012.11274	352412.6376	35049.6074
M559T12_3	1.60928821	-0.6864227	0.005163	DOWN	558.65606	11.946008	370365.7102	59781.37595	230142.5613	118499.986
M523T25_3	1.72519759	-0.7867616	0.005269	DOWN	523.351334	24.55665	83381.63324	30203.50657	48331.64249	11643.1038
M532T12_4	2.56763651	-1.360441	0.005303	DOWN	531.62637	11.722467	189169.4307	100232.6588	73674.5369	24981.0702
M590T11_1	1.17144771	-0.2282926	0.005547	DOWN	589.806349	10.709825	394767.6914	48353.46044	336991.3044	28747.7441
M600T11_2	1.22156515	-0.2887308	0.006234	DOWN	600.326316	10.85705	215982.2534	24368.02558	176807.8062	31338.349
M760T48_2	4.09555973	-2.0340606	0.006314	DOWN	760.211452	47.722867	180494.2447	121048.8977	44070.71479	46946.9251
M758T48	3.84367762	-1.9424873	0.006949	DOWN	758.211304	47.738183	294813.3963	197352.1811	76700.8645	68753.1807
M523T31_1	1.30216727	-0.3809148	0.00704	DOWN	523.238593	31.10335	217852.747	45315.3953	167300.1257	20311.0621
M526T12_4	1.33469776	-0.4165131	0.007119	DOWN	525.637502	12.027517	255976.922	48959.70638	191786.4328	45494.4499
M558T12_4	1.87564979	-0.9073905	0.00767	DOWN	557.673123	12.1229	105462.2221	45574.96077	56227.03273	9913.34732
M806T41	1.17562322	-0.2334258	0.007829	DOWN	805.539461	40.661458	269483.1232	25502.51837	229225.758	33593.2514
M227T0_1	1.04765037	-0.0671573	0.008288	DOWN	226.948737	0.3181	2448364.143	48542.97003	2337004.998	102086.912
M223T12	1.32473459	-0.4057033	0.008329	DOWN	223.09099	12.454167	411887.9551	70770.72578	310921.1146	80949.7709
M719T12_8	1.21691491	-0.2832283	0.008336	DOWN	719.404726	11.560775	582684.3936	82581.71407	478820.9815	73707.9632
M518T24_1	1.43250923	-0.5185444	0.008982	DOWN	518.315021	24.164367	840566.4155	240435.2852	586779.0571	79307.1105
M666T11_2	1.28983303	-0.3671843	0.009422	DOWN	666.365363	11.298992	232058.5294	48927.44523	179913.6198	22865.3529
M574T12_4	1.43098368	-0.5170072	0.009653	DOWN	573.665657	12.048492	261126.6203	38727.58712	182480.5023	73125.6195
M540T12_7	1.28519389	-0.361986	0.009684	DOWN	539.977913	12.129883	240871.2711	51275.01176	187420.181	17482.4653
M406T10_6	1.23242345	-0.301498	0.010186	DOWN	406.212001	10.357233	244419.6008	36989.54168	198324.3668	34774.4094
M759T48	2.92634263	-1.5490987	0.011138	DOWN	759.212622	47.736083	228183.1809	146556.4028	77975.55168	59995.6043
M522T25_3	1.36285353	-0.4466305	0.011142	DOWN	522.348735	25.156483	1743586.204	459794.2562	1279364.336	114171.246
M706T10_3	1.17558037	-0.2333732	0.011619	DOWN	706.220047	9.7677	222225.3194	20726.4969	189034.5612	30421.173
M727T10_3	1.18545481	-0.2454407	0.01166	DOWN	727.198284	9.751825	246490.4783	11649.13357	207929.0382	38391.8145
M523T25_2	1.30217736	-0.380926	0.012092	DOWN	523.351785	25.1565	455449.2757	105100.9144	349759.7869	39956.5793
M611T11_4	1.16610243	-0.2216945	0.012341	DOWN	611.31788	10.846042	835593.6758	81327.65403	716569.7046	106883.46

M519T24_2	1.36988184	-0.4540515	0.012634	DOWN	519.318222	24.164325	201344.2548	54630.30762	146979.2862	19751.2398
M726T36	6.6972363	-2.7435659	0.013272	DOWN	725.545791	35.993767	63949.36635	55946.70395	9548.620286	5363.79842
M687T46	2.76582141	-1.467708	0.013489	DOWN	687.192825	46.076483	248509.7808	160229.6868	89850.26284	66187.3651
M544T25_1	1.28634287	-0.3632752	0.013905	DOWN	544.330177	25.156483	683557.5916	156569.3611	531396.1062	48371.8604
M726T37	5.90931297	-2.5629904	0.013987	DOWN	725.54573	36.776667	59170.5126	51081.63962	10013.09508	5718.8259
M705T10_2	1.19878526	-0.2615733	0.014939	DOWN	705.217785	9.7677	657700.7091	64764.127	548639.3022	107230.448
M525T12_7	1.34999474	-0.4329538	0.015796	DOWN	525.302074	12.033208	332173.7625	80982.43673	246055.5973	61033.8094
M659T35	1.5765916	-0.656809	0.015891	DOWN	659.278251	35.044767	112770.0821	38614.90493	71527.77068	29608.2007
M739T12_2	1.30582344	-0.3849598	0.016571	DOWN	738.94024	11.685817	301642.0046	43995.34891	230997.5421	70286.9653
M567T11_3	1.17202521	-0.2290036	0.016586	DOWN	567.290981	10.524292	562111.977	82100.14965	479607.411	51393.7222
M413T0_3	1.39259522	-0.477776	0.017205	DOWN	413.260672	0.311325	100679.7139	3836.597397	72296.46664	30768.0614
M485T31	1.10593605	-0.145268	0.017366	DOWN	485.283125	31.092817	409947.2642	40701.33174	370678.9959	21034.8214
M576T11_7	1.35668731	-0.4400882	0.019306	DOWN	576.337591	10.693517	123028.2029	35599.12928	90682.79909	10307.2272
M634T11_4	1.25141488	-0.3235602	0.021759	DOWN	634.332604	11.013017	228642.6693	46428.57064	182707.3283	33569.4547
M522T25_2	1.26388228	-0.3378621	0.021854	DOWN	522.347912	24.567617	261880.8276	56032.67096	207203.4974	38658.4411
M353T33_2	2.32403769	-1.2166335	0.022517	DOWN	353.261427	33.276817	76510.9112	49969.26605	32921.5449	8669.12196
M384T10_6	1.25346501	-0.3259217	0.022845	DOWN	384.19921	10.11525	253504.6637	42244.64079	202243.1117	49392.8771
M489T19_1	23.2152288	-4.5369996	0.02476	DOWN	489.160396	18.733417	48224.42247	54230.93042	2077.275352	370.907408
M494T22	1.81623627	-0.8609519	0.025725	DOWN	494.317783	21.980583	103317.2957	53046.91733	56885.38295	23271.2445
M545T25_2	1.2796423	-0.3557406	0.02679	DOWN	545.333344	25.156558	190980.4585	49186.95458	149245.1902	14972.9873
M730T12_2	1.32717376	-0.4083573	0.027081	DOWN	729.925178	11.685783	191299.0284	40897.83227	144140.1526	46451.4486
M741T12_2	1.2054544	-0.2695771	0.028186	DOWN	740.915807	11.683092	644298.7322	131241.8709	534486.1912	36954.9729
M194T8_1	1.20152431	-0.2648658	0.028202	DOWN	194.078672	7.5491667	409595.6877	81408.27208	340896.7128	28309.9024
M426T10_3	1.17018399	-0.2267354	0.029085	DOWN	426.209236	10.239542	256366.352	36128.36444	219082.0876	34138.6459
M705T37	3.11738194	-1.6403349	0.030096	DOWN	704.568078	36.768367	38019.80086	31654.21741	12206.58791	4316.4201
M761T48_2	2.49261787	-1.3176617	0.032696	DOWN	761.210186	47.723467	77591.19034	57025.16952	31128.39372	19221.3558
M704T36	2.71717808	-1.4421091	0.035227	DOWN	703.564482	35.986458	83537.04192	67196.72356	30744.0438	9744.73344
M704T37_2	2.90558333	-1.5388278	0.035725	DOWN	703.56497	36.776567	90186.67476	75527.03527	31050.38144	10988.7289
M704T34_2	2.69717215	-1.4314476	0.036187	DOWN	703.56441	34.440817	66308.46683	53370.03945	24596.59934	9026.23392
M540T12_11	1.27758076	-0.3534145	0.038121	DOWN	540.312265	12.133183	211099.6473	47833.32313	165233.8968	43675.3043
M1070T19_1	2.00279162	-1.0020123	0.038191	DOWN	1069.53373	18.58125	60582.94209	39414.75463	30249.24884	5126.25014
