# Supplementary material for: Phosphatidylcholine could protect the defect of zearalenone exposure on follicular development and oocyte maturation
Source: Aging (Albany NY). 2018 Nov 25;10(11):3486–506. doi: 10.18632/aging.101660 (PMC6286824; doi:10.18632/aging.101660)
Supplement: Supplementary Table 5 [file aging-10-101660-s004.pdf]

Table S5. Prediction chemicals of the differential content metabolites in the GCs culture media of control and ZEA-treatment groups.

Mol_ID	Input mass	Adduct	Mass	D-ppm	Name	Formula	CAS	KEGG	MS/MS	Link
67869	659.28	[M+Na]+	636.284	5	Thalsimine	C38H40N2O7	5525-36-0	C09661	NO	metabo_info.php?molid=67869
82180	725.55	[M+Na]+	702.556	0	PA(O-18:0/19:1(9Z))	C40H79O7P			NO	metabo_info.php?molid=82180
82196	725.55	[M+Na]+	702.556	0	PA(O-20:0/17:1(9Z))	C40H79O7P			NO	metabo_info.php?molid=82196
82263	725.55	[M+Na]+	702.556	0	PA(P-16:0/21:0)	C40H79O7P			NO	metabo_info.php?molid=82263
82284	725.55	[M+Na]+	702.556	0	PA(P-18:0/19:0)	C40H79O7P			NO	metabo_info.php?molid=82284
82304	725.55	[M+Na]+	702.556	0	PA(P-20:0/17:0)	C40H79O7P			NO	metabo_info.php?molid=82304
82180	725.55	[M+Na]+	702.556	0	PA(O-18:0/19:1(9Z))	C40H79O7P			NO	metabo_info.php?molid=82180
82196	725.55	[M+Na]+	702.556	0	PA(O-20:0/17:1(9Z))	C40H79O7P			NO	metabo_info.php?molid=82196
82263	725.55	[M+Na]+	702.556	0	PA(P-16:0/21:0)	C40H79O7P			NO	metabo_info.php?molid=82263
82284	725.55	[M+Na]+	702.556	0	PA(P-18:0/19:0)	C40H79O7P			NO	metabo_info.php?molid=82284
82304	725.55	[M+Na]+	702.556	0	PA(P-20:0/17:0)	C40H79O7P			NO	metabo_info.php?molid=82304
92066	760.21	[M+H]+	759.198	7	Delphinidin 3-lathyroside 5-glucoside	C32H39O21			NO	metabo_info.php?molid=92066
68069	517.29	[M+Na]+	494.297	1	Thiobinupharidine	C30H42N2O2S	30343-72-7	C09990	NO	metabo_info.php?molid=68069
49167	468.95	[M+Na]+	445.961	4	Luteolin 7,3\'-disulfate	C15H10O12S2			NO	metabo_info.php?molid=49167
50415	468.95	[M+Na]+	445.961	4	Kaempferol 3,7-di-O-sulfate	C15H10O12S2			NO	metabo_info.php?molid=50415
2477	684.19	[M+Na]+	661.211	7	Spectinomycin adenylate	C24H36N7O13P	27544-31-6	C03580	NO	metabo_info.php?molid=2477
3587	529.97	[M+Na]+	506.986	1	3\'-Phosphoadenylyl sulfate (PAPS)	C10H15N5O13P2S	482-67-7	C00053	YES	metabo_info.php?molid=3587
65998	529.97	[M+Na]+	506.986	1	2\'-Phosphoadenylylsulfate	C10H15N5O13P2S		C03568	NO	metabo_info.php?molid=65998
51151	685.2	[M+H]+	684.19	3	5,2\',4\'-Trihydroxy-3,7,5\'-trimethoxyflavone 2\'-galactosyl-(1->4)-glucoside	C30H36O18			NO	metabo_info.php?molid=51151
51347	685.2	[M+H]+	684.19	3	Chrysosplenol C 6,4\'-diglucoside	C30H36O18			NO	metabo_info.php?molid=51347
51384	685.2	[M+H]+	684.19	3	Veronicafolin 3-glucosyl-(1->3)-galactoside	C30H36O18			NO	metabo_info.php?molid=51384

51694	685.2	[M+H]+	684.19	3	3,5,2\'-Trihydroxy-7,8,4\'-trimethoxyflavone 5-glucosyl-(1->2)-galactoside	C30H36O18			NO	metabo_info.php?molid=51694
51715	685.2	[M+H]+	684.19	3	7,4\'-Dihydroxy-3,5,6,8-tetramethoxyflavone 4\'-glucosyl-(1->3)-galactoside	C30H36O18			NO	metabo_info.php?molid=51715
51733	685.2	[M+H]+	684.19	3	Limocitrol 3-neohesperidoside	C30H36O18			NO	metabo_info.php?molid=51733
69675	545.97	[M+Na]+	522.973	9	Adenosine 5-O-(3-thiotriphophate)	C10H16N5O12P3S		C13742	NO	metabo_info.php?molid=69675
82180	703.56	[M+H]+	702.556	1	PA(O-18:0/19:1(9Z))	C40H79O7P			NO	metabo_info.php?molid=82180
82196	703.56	[M+H]+	702.556	1	PA(O-20:0/17:1(9Z))	C40H79O7P			NO	metabo_info.php?molid=82196
82263	703.56	[M+H]+	702.556	1	PA(P-16:0/21:0)	C40H79O7P			NO	metabo_info.php?molid=82263
82284	703.56	[M+H]+	702.556	1	PA(P-18:0/19:0)	C40H79O7P			NO	metabo_info.php?molid=82284
82304	703.56	[M+H]+	702.556	1	PA(P-20:0/17:0)	C40H79O7P			NO	metabo_info.php?molid=82304
44057	487.27	[M+H]+	486.262	5	3α-HYDROXYDEOXODIHYDROGEDUNIN	C28H38O7			NO	metabo_info.php?molid=44057
44451	487.27	[M+H]+	486.262	5	1,3-DIDEACETYLDEOXYKHIVORIN	C28H38O7			YES	metabo_info.php?molid=44451
43758	487.27	[M+H]+	486.262	5	3beta-HYDROXYDEOXODIHYDROGEDUNIN	C28H38O7			NO	metabo_info.php?molid=43758
84268	487.27	[M+H]+	486.262	5	15beta-Hydroxynicandrin B	C28H38O7			NO	metabo_info.php?molid=84268
89072	487.27	[M+H]+	486.262	5	(4beta,5beta,6beta,14beta,15alpha,20S,22R)-5,6-Epoxy-4,14,15-trihydroxy-1-oxowitha-2,24-dienolide	C28H38O7	222609-98-5		NO	metabo_info.php?molid=89072
89699	487.27	[M+H]+	486.262	5	Withaperuvin C	C28H38O7	81644-34-0		NO	metabo_info.php?molid=89699
89769	487.27	[M+H]+	486.262	5	24,25-Epoxywithanolide D	C28H38O7			NO	metabo_info.php?molid=89769
95541	487.27	[M+H]+	486.262	5	Eucalyptone	C28H38O7	172617-99-1		NO	metabo_info.php?molid=95541

428	487.27	[M+H]+	486.262	5	Bongkrekic acid	C28H38O7	11076-19-0		NO	metabo_info.php?molid=428
73475	500.29	[M+H]+	499.278	8	Aconine	C25H41NO9	509-20-6	C19990	NO	metabo_info.php?molid=73475
69483	498.3	[M+Na]+	475.315	0	Dinoprost tromethamine	C24H45NO8	38562-01-5	C12786	NO	metabo_info.php?molid=69483
51968	613.17	[M+H]+	612.169	2	Okanin 3\',4\'-diglucoside	C27H32O16			NO	metabo_info.php?molid=51968
51970	613.17	[M+H]+	612.169	2	Okanin 4\'-gentiobioside	C27H32O16			NO	metabo_info.php?molid=51970
52849	613.17	[M+H]+	612.169	2	Eriodictyol 5,3\'-di-O-glucoside	C27H32O16			NO	metabo_info.php?molid=52849
53093	613.17	[M+H]+	612.169	2	5,6,7,4\'-Tetrahydroxyflavanone 6,7-diglucoside	C27H32O16			NO	metabo_info.php?molid=53093
87060	613.17	[M+H]+	612.169	2	Safflomin A	C27H32O16	78281-02-4		NO	metabo_info.php?molid=87060
94666	613.17	[M+H]+	612.169	2	Carthamidin 6,7-diglucoside	C27H32O16			NO	metabo_info.php?molid=94666
95046	613.17	[M+H]+	612.169	2	Aromadendrin 3,7-diglucoside	C27H32O16	80212-10-8		NO	metabo_info.php?molid=95046
95152	613.17	[M+H]+	612.169	2	Hydroxysafflor yellow A	C27H32O16	146087-19-6		NO	metabo_info.php?molid=95152
67110	502.28	[M+Na]+	479.288	3	Delcorine	C26H41NO7	52358-55-1	C08675	NO	metabo_info.php?molid=67110
51982	611.18	[M+H]+	610.169	1	Okanin 4-methyl ether 4\'-O-(6\'\'-O-p-coumaroylglucoside)	C31H30O13			NO	metabo_info.php?molid=51982
53094	611.18	[M+H]+	610.169	1	4\'-O-Methylcarthamidin 7-(2-p-coumaroylglucoside)	C31H30O13			NO	metabo_info.php?molid=53094
558	611.18	[M+Na]+	588.184	5	Etoposide	C29H32O13	33419-42-0	C01576	YES	metabo_info.php?molid=558
48042	611.18	[M+Na]+	588.184	5	Dalbinol O-glucoside	C29H32O13			NO	metabo_info.php?molid=48042
48578	611.18	[M+Na]+	588.184	5	Matteuorienate C	C29H32O13			NO	metabo_info.php?molid=48578
704	611.18	[M+Na]+	588.184	5	cis-Etoposide	C29H32O13	100007-56-5		NO	metabo_info.php?molid=704

45727	459.23	[M+H]+	458.228	3	17-trifluoromethylphenyl-13,14-dihydro trinor PGF2α	C24H33F3O5	NA		YES	metabo_info.php?molid=45727
43704	459.23	[M+H]+	458.23	8	1,3-DIDEACETYL-7-DEACETOXY-7-OXOKHIVORIN	C26H34O7			YES	metabo_info.php?molid=43704
43842	459.23	[M+H]+	458.23	8	1-DEACETOXY-1-OXO-3,7-DIDEACETYLKHIVORIN	C26H34O7			YES	metabo_info.php?molid=43842
53373	459.23	[M+H]+	458.23	8	fumagillin	C26H34O7		C0966 8	YES	metabo_info.php?molid=53373
67233	459.23	[M+H]+	458.23	8	Hellebrigenin 3-acetate	C26H34O7	#####	C0880 7	NO	metabo_info.php?molid=67233
71444	459.23	[M+H]+	458.23	8	Cinobufotalin	C26H34O7	1108-68-5	C1693 2	NO	metabo_info.php?molid=71444
90186	459.23	[M+H]+	458.23	8	Hydroxystrobilurin D	C26H34O7	11348 9-65-9		NO	metabo_info.php?molid=90186
49554	687.19	[M+Na]+	664.2	4	Pectolinarigenin 7-(4\'\'\'-acetylrutinoside)	C31H36O16			NO	metabo_info.php?molid=49554
49563	687.19	[M+Na]+	664.2	4	Scutellarein 6,4\'-dimethyl ether 7-(2\'\'\'-acetylrutinoside)	C31H36O16			NO	metabo_info.php?molid=49563
49564	687.19	[M+Na]+	664.2	4	Scutellarein 6,4\'-dimethyl ether 7-(3\'\'\'-acetylrutinoside)	C31H36O16			NO	metabo_info.php?molid=49564
71033	516.28	[M+Na]+	493.29	5	Syringolin A	C24H39N5O6		C1570 1	NO	metabo_info.php?molid=71033
93009	474.24	[M+Na]+	451.251	6	Enterocin 900	C31H33NO2	17953 0-08-6		NO	metabo_info.php?molid=93009
82180	703.56	[M+H]+	702.556	1	PA(O-18:0/19:1(9Z))	C40H79O7P			NO	metabo_info.php?molid=82180
82196	703.56	[M+H]+	702.556	1	PA(O-20:0/17:1(9Z))	C40H79O7P			NO	metabo_info.php?molid=82196
82263	703.56	[M+H]+	702.556	1	PA(P-16:0/21:0)	C40H79O7P			NO	metabo_info.php?molid=82263
82284	703.56	[M+H]+	702.556	1	PA(P-18:0/19:0)	C40H79O7P			NO	metabo_info.php?molid=82284
82304	703.56	[M+H]+	702.556	1	PA(P-20:0/17:0)	C40H79O7P			NO	metabo_info.php?molid=82304
82180	703.56	[M+H]+	702.556	1	PA(O-18:0/19:1(9Z))	C40H79O7P			NO	metabo_info.php?molid=82180
82196	703.56	[M+H]+	702.556	1	PA(O-20:0/17:1(9Z))	C40H79O7P			NO	metabo_info.php?molid=82196
82263	703.56	[M+H]+	702.556	1	PA(P-16:0/21:0)	C40H79O7P			NO	metabo_info.php?molid=82263
82284	703.56	[M+H]+	702.556	1	PA(P-18:0/19:0)	C40H79O7P			NO	metabo_info.php?molid=82284

82304	703.56	[M+H]+	702.556	1	PA(P-20:0/17:0)	C40H79O7P			NO	metabo_info.php?molid=82304
73359	503.26	[M+H]+	502.25	4	Streptothricin F	C19H34N8O8	3808-42-2	C19785	NO	metabo_info.php?molid=73359
44045	503.26	[M+H]+	502.257	8	1,3-DIDEACETYLKHIVORIN	C28H38O8			NO	metabo_info.php?molid=44045
86914	503.26	[M+H]+	502.257	8	Cavipetin B	C28H38O8	128530-03-0		NO	metabo_info.php?molid=86914
87608	503.26	[M+H]+	502.257	8	28-Hydroxywithanolide E	C28H38O8	188346-67-0		NO	metabo_info.php?molid=87608
89633	503.26	[M+H]+	502.257	8	Withaperuvin G	C28H38O8			NO	metabo_info.php?molid=89633
89687	503.26	[M+H]+	502.257	8	4beta-Hydroxywithanolide E	C28H38O8	54334-04-2		NO	metabo_info.php?molid=89687
89859	503.26	[M+H]+	502.257	8	Ixocarpalactone B	C28H38O8	71801-44-0		NO	metabo_info.php?molid=89859
89907	503.26	[M+H]+	502.257	8	Withaperuvin F	C28H38O8			NO	metabo_info.php?molid=89907
42025	509.29	[M+H]+	508.278	9	26,26,26,27,27,27-hexafluoro-1αa-hydroxyvitamin D3 / 26,26,26,27,27,27-hexafluoro-1αa-hydroxycholecalciferol	C27H38F6O2			NO	metabo_info.php?molid=42025
42026	509.29	[M+H]+	508.278	9	26,26,26,27,27,27-hexafluoro-25-hydroxyvitamin D3 / 26,26,26,27,27,27-hexafluoro-25-hydroxycholecalciferol	C27H38F6O2			NO	metabo_info.php?molid=42026
80002	509.29	[M+H]+	508.28	4	PG(18:2(9Z,12Z)/0:0)	C24H45O9P			NO	metabo_info.php?molid=80002
41876	509.29	[M+Na]+	486.298	4	Hydrocortisone cypionate	C29H42O6		C08176	NO	metabo_info.php?molid=41876
48835	761.21	[M+Na]+	738.216	6	Apigenin 7-rhamnosyl-(1->6)-(4\'\'-E-p-methoxycinnamoylglucoside)	C37H38O16			NO	metabo_info.php?molid=48835
57800	531.29	[M+H]+	530.288	5	Proscillaridin A	C30H42O8		C12816	NO	metabo_info.php?molid=57800

86915	531.29	[M+H]+	530.288	5	Cavipetin A	C30H42O8	12853 0-02-9		NO	metabo_info.php?molid=86915
88915	531.29	[M+H]+	530.288	5	7,12-Dihydroxy-3,11,15,23-tetraoxolanost-8-en-26-oic acid	C30H42O8	97653 -94-6		NO	metabo_info.php?molid=88915
89538	531.29	[M+H]+	530.288	5	Physapubescin	C30H42O8	74747 -52-7		NO	metabo_info.php?molid=89538
90625	531.29	[M+H]+	530.288	5	Ganoderic acid N	C30H42O8	11024 1-19-5		NO	metabo_info.php?molid=90625
91455	531.29	[M+H]+	530.288	5	Ganoderic acid theta	C30H42O8	29467 4-15-0		NO	metabo_info.php?molid=91455
41673	515.3	[M+H]+	514.293	2	Cucurbitacin I	C30H42O7		C0880 0	NO	metabo_info.php?molid=41673
53678	515.3	[M+H]+	514.293	2	(-)-Euphomine A	C30H42O7			NO	metabo_info.php?molid=53678
69323	515.3	[M+H]+	514.293	2	Stigmatellin A	C30H42O7	91682 -96-1	C1214 8	NO	metabo_info.php?molid=69323
90677	515.3	[M+H]+	514.293	2	Ganoderenic acid A	C30H42O7	10066 5-40-5		NO	metabo_info.php?molid=90677
90897	515.3	[M+H]+	514.293	2	Ganoderic acid C1	C30H42O7	95311 -97-0		NO	metabo_info.php?molid=90897
90968	515.3	[M+H]+	514.293	2	Ganoderenic acid B	C30H42O7	10066 5-41-6		NO	metabo_info.php?molid=90968
91453	515.3	[M+H]+	514.293	2	Ganoderic acid xi	C30H42O7	29467 4-09-2		NO	metabo_info.php?molid=91453
91545	515.3	[M+H]+	514.293	2	Ganosporelactone B	C30H42O7	13800 8-05-6		NO	metabo_info.php?molid=91545
94196	515.3	[M+H]+	514.293	2	Ganoderic acid J	C30H42O7	10044 0-26-4		NO	metabo_info.php?molid=94196
94204	515.3	[M+H]+	514.293	2	Ganoderic acid V1	C30H42O7	15003 3-91-3		NO	metabo_info.php?molid=94204
2098	515.3	[M+H]+	514.294	3	Probucol spiroquinone	C31H46O2S2	81075 -13-0		NO	metabo_info.php?molid=2098
43221	353.26	[M+H]+	352.251	7	isopropamide	C23H32N2O			YES	metabo_info.php?molid=43221
85059	353.26	[M+H]+	352.251	7	AB005	C23H32N2O	89515 5-25-6		NO	metabo_info.php?molid=85059

49683	512.98	[M+Na]+	489.988	4	6-Hydroxyluteolin 6,3\'-dimethyl ether 7,4\'-disulfate	C17H14O13S2			NO	metabo_info.php?molid=49683
51068	512.98	[M+Na]+	489.988	4	Ombuin 3,3\'-di-O-sulfate	C17H14O13S2			NO	metabo_info.php?molid=51068
67229	547.29	[M+H]+	546.283	9	Decoside	C30H42O9	111508-63-5	C08860	NO	metabo_info.php?molid=67229
71004	654.32	[M+H]+	653.321	8	A 80987	C37H43N5O6	144141-97-9	C15661	NO	metabo_info.php?molid=71004
93717	530.96	[M+Na]+	507.967	2	Tartrazine calcium lake	C16H12CaN4O9S2			NO	metabo_info.php?molid=93717
72537	527.99	[M+H]+	526.985	7	Sulfluramid	C10H6F17NO2S	4151-50-2	C18766	NO	metabo_info.php?molid=72537
5984	527.99	[M+H]+	526.986	8	2-Amino-4-oxo-6-(erythro-1,2,3-trihydroxyprolyl)-7,8-dihydroxypteridine triphosphate	C9H16N5O15P3			NO	metabo_info.php?molid=5984
81200	559.32	[M+H]+	558.317	1	PI(O-16:0/0:0)	C25H51O11P			NO	metabo_info.php?molid=81200
41667	559.32	[M+H]+	558.319	3	Cucurbitacin B	C32H46O8	6199-67-3	C08794	YES	metabo_info.php?molid=41667
45669	559.32	[M+Na]+	536.34	7	13(S)-HODE-biotin	C28H48N4O4S	NA		YES	metabo_info.php?molid=45669
41666	575.32	[M+H]+	574.314	6	Cucurbitacin A	C32H46O9		C08793	NO	metabo_info.php?molid=41666
88916	575.32	[M+H]+	574.314	6	Ganoderic acid alpha	C32H46O9	220181-81-7		NO	metabo_info.php?molid=88916
91171	575.32	[M+H]+	574.314	6	Ganoderic acid K	C32H46O9	104700-95-0		NO	metabo_info.php?molid=91171
6706	575.32	[M+Na]+	552.33	2	Lithocholate 3-O-glucuronide	C30H48O9			NO	metabo_info.php?molid=6706
68091	575.32	[M+Na]+	552.331	4	Pandamine	C31H44N4O5	10233-81-5	C10012	NO	metabo_info.php?molid=68091
78873	673.4	[M+Na]+	650.416	2	PG(12:0/15:1(9Z))	C33H63O10P			NO	metabo_info.php?molid=78873
78895	673.4	[M+Na]+	650.416	2	PG(13:0/14:1(9Z))	C33H63O10P			NO	metabo_info.php?molid=78895
78935	673.4	[M+Na]+	650.416	2	PG(14:1(9Z)/13:0)	C33H63O10P			NO	metabo_info.php?molid=78935
78986	673.4	[M+Na]+	650.416	2	PG(15:1(9Z)/12:0)	C33H63O10P			NO	metabo_info.php?molid=78986
92150	523.35	[M+H]+	522.34	6	Polysorbate 20	C26H50O10	9005-64-5		NO	metabo_info.php?molid=92150

71023	523.35	[M+Na]+	500.361	1	Vicenistatin	C30H48N2O4	150999-05-6	C15688	NO	metabo_info.php?molid=71023
40316	542.33	[M+H]+	541.317	8	PC(20:5(5Z,8Z,11Z,14Z,17Z)/0:0)	C28H48NO7P			NO	metabo_info.php?molid=40316
65656	561.96	[M+Na]+	538.968	5	GTP-gamma-S	C10H16N5O13P3S		C01806	NO	metabo_info.php?molid=65656
723	561.96	[M+Na]+	538.968	5	6-Thioguanosine triphosphate	C10H16N5O13P3S	17670-19-8		NO	metabo_info.php?molid=723
92968	465.28	[M+Na]+	442.293	5	Ascorbyl stearate	C24H42O7	10605-09-1		NO	metabo_info.php?molid=92968
65429	588.34	[M+Na]+	565.352	0	Antillatoxin B (red color)	C33H47N3O5			YES	metabo_info.php?molid=65429
6132	587.33	[M+H]+	586.319	3	Presqualene diphosphate	C30H52O7P2		C03428	NO	metabo_info.php?molid=6132
53853	587.33	[M+H]+	586.319	3	all-trans-hexaprenyl diphosphate	C30H52O7P2		C01230	NO	metabo_info.php?molid=53853
67094	587.33	[M+H]+	586.325	7	Inuline	C32H46N2O8	22413-78-1	C08659	NO	metabo_info.php?molid=67094
80015	587.33	[M+Na]+	564.343	6	PG(22:2(13Z,16Z)/0:0)	C28H53O9P			NO	metabo_info.php?molid=80015
67384	565.31	[M+H]+	564.31	4	Bonafousine	C35H40N4O3	60820-65-7	C09050	NO	metabo_info.php?molid=67384
95773	609.34	[M+H]+	608.335	2	24-Acetyl- 25-cinnamoylvulgaroside	C36H48O8	172616-89-6		NO	metabo_info.php?molid=95773
81199	609.34	[M+Na]+	586.348	5	PI(O-18:0/0:0)	C27H55O11P			NO	metabo_info.php?molid=81199
63699	609.34	[M+Na]+	586.351	1	6,8a-Seco-6,8a-deoxy-5-oxoavermectin \'\'2a\'\' aglycone	C34H50O8		C11969	NO	metabo_info.php?molid=63699
67637	659.28	[M+Na]+	636.284	8	Calafatimine	C38H40N2O7	77793-42-1	C09369	NO	metabo_info.php?molid=67637
67869	659.28	[M+Na]+	636.284	8	Thalsimine	C38H40N2O7	5525-36-0	C09661	NO	metabo_info.php?molid=67869
72231	377.04	[M+Na]+	354.049	6	Thiodicarb	C10H18N4O4S3	59669-26-0	C18423	YES	metabo_info.php?molid=72231
66420	377.04	[M+Na]+	354.054	6	WIN56291	C16H16Cl2N2O3		C06490	NO	metabo_info.php?molid=66420
67283	675.38	[M+H]+	674.367	8	Cimicifugoside	C37H54O11	66176-93-0	C08935	NO	metabo_info.php?molid=67283

68083	675.38	[M+H]+	674.379	9	Hymenocardine	C37H50N6O6	19771-56-3	C10004	NO	metabo_info.php?molid=68083
66652	631.35	[M+H]+	630.345	1	Remikiren	C33H50N4O6S	126222-34-2	C07465	NO	metabo_info.php?molid=66652
86851	497.34	[M+Na]+	474.346	3	Dehydrocarpaine II	C28H46N2O4	72362-03-9		NO	metabo_info.php?molid=86851
912	329.18	[M+H]+	328.179	9	Labetalol	C19H24N2O3	36894-69-6	C07063	YES	metabo_info.php?molid=912
66416	329.18	[M+H]+	328.179	9	Compound V(S)	C19H24N2O3		C06494	NO	metabo_info.php?molid=66416
73469	329.18	[M+H]+	328.179	9	Gelsedine	C19H24N2O3	7096-96-0	C19982	NO	metabo_info.php?molid=73469
64710	329.18	[M+Na]+	306.194	1	W123	C17H26N2O3	NA		YES	metabo_info.php?molid=64710
35919	329.18	[M+Na]+	306.196	7	9-chloro-10-hydroxy-hexadecanoic acid	C16H31ClO3		C13949	NO	metabo_info.php?molid=35919
96785	329.18	[M+Na]+	306.196	7	9-hydroxy-10-chloro-hexadecanoic acid	C16H31ClO3			NO	metabo_info.php?molid=96785
67520	519.32	[M+H]+	518.316	9	Hodgkinsine	C33H38N6	18210-71-4	C09211	NO	metabo_info.php?molid=67520
68035	525.3	[M+Na]+	502.308	8	Kolanone	C33H42O4	81827-55-6	C09948	NO	metabo_info.php?molid=68035
65437	520.33	[M+Na]+	497.335	3	Tumonoic Acid I	C27H47NO7			YES	metabo_info.php?molid=65437
81188	653.37	[M+H]+	652.359	0	PI(22:2(13Z,16Z)/0:0)	C31H57O12P			NO	metabo_info.php?molid=81188
89978	653.37	[M+Na]+	630.377	1	2,3,23-Triacetylsericic acid	C36H54O9	58096-15-4		NO	metabo_info.php?molid=89978
90636	653.37	[M+Na]+	630.377	1	Ganoderic acid Mb	C36H54O9			NO	metabo_info.php?molid=90636
90981	653.37	[M+Na]+	630.377	1	Ganoderic acid Mc	C36H54O9			NO	metabo_info.php?molid=90981
65447	608.38	[M+H]+	607.372	2	Malyngamide J	C33H53NO9			YES	metabo_info.php?molid=65447
92842	391.28	[M+Na]+	368.293	5	Octadecyl fumarate	C22H40O4	1741-93-1		NO	metabo_info.php?molid=92842
92150	523.35	[M+H]+	522.34	7	Polysorbate 20	C26H50O10	9005-64-5		NO	metabo_info.php?molid=92150
71023	523.35	[M+Na]+	500.361	2	Vicenistatin	C30H48N2O4	150999-05-6	C15688	NO	metabo_info.php?molid=71023

68077	666.37	[M+H]+	665.358	0	Amphibine B	C39H47N5O5	38541-74-1	C09998	NO	metabo_info.php?molid=68077
92249	666.37	[M+H]+	665.358	0	Jubanine C	C39H47N5O5	159226-00-3		NO	metabo_info.php?molid=92249
65656	539.98	[M+H]+	538.968	5	GTP-gamma-S	C10H16N5O13P3S		C01806	NO	metabo_info.php?molid=65656
723	539.98	[M+H]+	538.968	5	6-Thioguanosine triphosphate	C10H16N5O13P3S	17670-19-8		NO	metabo_info.php?molid=723
92150	545.33	[M+Na]+	522.34	6	Polysorbate 20	C26H50O10	9005-64-5		NO	metabo_info.php?molid=92150
85482	540.31	[M+Na]+	517.323	0	Retapamulin	C30H47NO4S	224452-66-8		NO	metabo_info.php?molid=85482
77743	738.44	[M+Na]+	715.442	9	PS(13:0/18:3(6Z,9Z,12Z))	C37H66NO10P			NO	metabo_info.php?molid=77743
77744	738.44	[M+Na]+	715.442	9	PS(13:0/18:3(9Z,12Z,15Z))	C37H66NO10P			NO	metabo_info.php?molid=77744
77782	738.44	[M+Na]+	715.442	9	PS(14:1(9Z)/17:2(9Z,12Z))	C37H66NO10P			NO	metabo_info.php?molid=77782
77945	738.44	[M+Na]+	715.442	9	PS(17:2(9Z,12Z)/14:1(9Z))	C37H66NO10P			NO	metabo_info.php?molid=77945
78031	738.44	[M+Na]+	715.442	9	PS(18:3(6Z,9Z,12Z)/13:0)	C37H66NO10P			NO	metabo_info.php?molid=78031
78062	738.44	[M+Na]+	715.442	9	PS(18:3(9Z,12Z,15Z)/13:0)	C37H66NO10P			NO	metabo_info.php?molid=78062
72057	738.44	[M+Na]+	715.447	3	2,2',3,3',4',5,6'-Heptabromodiphenyl ether	C12H3Br7O		C18139	NO	metabo_info.php?molid=72057
72058	738.44	[M+Na]+	715.447	3	2,2',3,4,4',5',6-Heptabromodiphenyl ether	C12H3Br7O	207122-16-5	C18140	NO	metabo_info.php?molid=72058
65956	373.21	[M+H]+	372.201	0	(Ac)2-L-Lys-D-Ala-D-Ala	C16H28N4O6		C03326	NO	metabo_info.php?molid=65956
1799	373.21	[M+Na]+	350.221	3	Perindopril lactam	C19H30N2O4	129970-98-5		NO	metabo_info.php?molid=1799
85136	406.21	[M+Na]+	383.225	5	JWH011	C27H29NO	155471-13-9		YES	metabo_info.php?molid=85136
85148	406.21	[M+Na]+	383.225	5	JWH182	C27H29NO	824960-02-3		YES	metabo_info.php?molid=85148
85150	406.21	[M+Na]+	383.225	5	JWH213	C27H29NO	824959-83-3		YES	metabo_info.php?molid=85150
78851	600.33	[M+Na]+	577.338	1	PS(22:2(13Z,16Z)/0:0)	C28H52NO9P			NO	metabo_info.php?molid=78851
90958	719.4	[M+Na]+	696.408	9	Momordicoside E	C37H60O12	78887-74-8		NO	metabo_info.php?molid=90958

92882	719.4	[M+Na]+	696.408	9	Glucosyl passiflorate	C37H60O12	1392-82-1		NO	metabo_info.php?molid=92882
58223	589.3	[M+H]+	588.295	4	D-Urobilin	C33H40N4O6	3947-38-4	C05795	NO	metabo_info.php?molid=58223
73308	589.3	[M+H]+	588.295	4	Dinoflagellate luciferin	C33H40N4O6		C19704	NO	metabo_info.php?molid=73308
36732	194.08	[M+Na]+	171.09	0	N-butanoyl-lhomoserine lactone	C8H13NO3	67605-85-0	C11837	YES	metabo_info.php?molid=36732
68218	194.08	[M+Na]+	171.09	0	Crotanecine	C8H13NO3	5096-50-4	C10284	NO	metabo_info.php?molid=68218
6386	194.08	[M+Na]+	171.09	0	(3-Methylcrotonyl)glycine methyl ester	C8H13NO3			NO	metabo_info.php?molid=6386
1E+05	804.54	[M+Na]+	781.547	1	PI-Cer(d18:0/16:0)	C40H80NO11P			NO	metabo_info.php?molid=103135
64483	727.2	[M+H]+	726.191	0	Lampranthin II	C34H34N2O16	32074-65-0	C08552	NO	metabo_info.php?molid=64483
67055	727.2	[M+H]+	726.191	0	Gomphrenin-V	C34H34N2O16	16955-52-5	C08547	NO	metabo_info.php?molid=67055
52735	727.2	[M+H]+	726.195	5	Prunin 3\'\',6\'\'-di-p-coumarate	C39H34O14			NO	metabo_info.php?molid=52735
46739	805.54	[M+H]+	804.531	2	1-(8-[3]-ladderane-octanoyl)-2-(8-[3]-ladderane-octanyl)-sn-glycero-3-phospho-(1\'-sn-glycerol)	C46H77O9P			NO	metabo_info.php?molid=46739
79896	805.54	[M+Na]+	782.546	5	PG(O-18:0/20:5(5Z,8Z,11Z,14Z,17Z))	C44H79O9P			NO	metabo_info.php?molid=79896
79931	805.54	[M+Na]+	782.546	5	PG(P-16:0/22:4(7Z,10Z,13Z,16Z))	C44H79O9P			NO	metabo_info.php?molid=79931
79976	805.54	[M+Na]+	782.546	5	PG(P-20:0/18:4(6Z,9Z,12Z,15Z))	C44H79O9P			NO	metabo_info.php?molid=79976
79994	805.54	[M+Na]+	782.546	5	PG(P-18:0/20:4(5Z,8Z,11Z,14Z))	C44H79O9P			NO	metabo_info.php?molid=79994
71005	567.29	[M+H]+	566.278	9	Dmp 323	C35H38N2O5	151867-81-1	C15662	NO	metabo_info.php?molid=71005

6655	567.29	[M+H]+	566.289	9	Mesoporphyrin IX	C34H38N4O4			NO	metabo_info.php?molid=6655
69112	611.32	[M+H]+	610.311	1	BQ 123	C31H42N6O7	136553-81-6	C11587	NO	metabo_info.php?molid=69112
63688	611.32	[M+Na]+	588.33	1	Avermectin B2b aglycone	C33H48O9		C11955	NO	metabo_info.php?molid=63688
40874	485.28	[M+H]+	484.28	8	PG(16:0/0:0)[U]	C22H45O9P			YES	metabo_info.php?molid=40874
46733	485.28	[M+H]+	484.28	8	PG(16:0/0:0)	C22H45O9P			NO	metabo_info.php?molid=46733
57617	485.28	[M+Na]+	462.298	8	3-dehydroecdysone	C27H42O6		C02513	NO	metabo_info.php?molid=57617
57625	485.28	[M+Na]+	462.298	8	2-dehydroecdysone	C27H42O6			NO	metabo_info.php?molid=57625
57638	485.28	[M+Na]+	462.298	8	shidasterone	C27H42O6			NO	metabo_info.php?molid=57638
67216	485.28	[M+Na]+	462.298	8	Podecdysone B	C27H42O6	22612-27-7	C08832	NO	metabo_info.php?molid=67216
91159	485.28	[M+Na]+	462.298	8	Lucidenic acid M	C27H42O6	110241-33-3		NO	metabo_info.php?molid=91159
69492	226.95	[M+H]+	225.944	9	2,5-Dichloro-4-oxohex-2-enedioate	C6H4Cl2O5		C12835	NO	metabo_info.php?molid=69492
92458	356.06	[M+H]+	355.051	9	Quinoline yellow	C18H13NO5S	8004-92-0		NO	metabo_info.php?molid=92458
72505	356.06	[M+H]+	355.055	0	Fluchloralin	C12H13ClF3N3O4	33245-39-5	C18730	NO	metabo_info.php?molid=72505
3939	356.06	[M+Na]+	333.073	0	bromodiphenhydramine	C17H20BrNO			NO	metabo_info.php?molid=3939
58054	731.09	[M+Na]+	708.105	3	Diguanosine diphosphate	C20H26N10O15P2	34692-44-9		NO	metabo_info.php?molid=58054
72013	731.09	[M+Na]+	708.105	3	5\'-Phosphoguanylyl(3\'->5\')guanosine	C20H26N10O15P2		C18076	NO	metabo_info.php?molid=72013
92919	167.09	[M+H]+	166.082	8	3-(4-Methyl-3-pentenyl)thiophene	C10H14S	62469-65-2		NO	metabo_info.php?molid=92919
67863	697.35	[M+H]+	696.341	6	Thalicarpine	C41H48N2O8	5373-42-2	C09655	NO	metabo_info.php?molid=67863
67283	697.35	[M+Na]+	674.367	4	Cimicifugoside	C37H54O11	66176-93-0	C08935	NO	metabo_info.php?molid=67283
85819	263.14	[M+H]+	262.135	1	Isoleucyl-Methionine	C11H22N2O3S			NO	metabo_info.php?molid=85819
85838	263.14	[M+H]+	262.135	1	Leucyl-Methionine	C11H22N2O3S			NO	metabo_info.php?molid=85838
85878	263.14	[M+H]+	262.135	1	Methionyl-Isoleucine	C11H22N2O3S			NO	metabo_info.php?molid=85878
85879	263.14	[M+H]+	262.135	1	Methionyl-Leucine	C11H22N2O3S			NO	metabo_info.php?molid=85879

87844	263.14	[M+H]+	262.136	0	Alnustone	C19H18O	33457-62-4		NO	metabo_info.php?molid=87844
66981	263.14	[M+Na]+	240.151	8	Dehydrofalcarinone	C17H20O	#####	C0844 5	NO	metabo_info.php?molid=66981
7089	166.08	[M+Na]+	143.095	3	Proline betaine	C7H13NO2		C1017 2	NO	metabo_info.php?molid=7089
66118	166.08	[M+Na]+	143.095	3	4-(Trimethylammonio)but-2-enoate	C7H13NO2		C0411 4	NO	metabo_info.php?molid=66118
86954	166.08	[M+Na]+	143.095	3	L-2-Amino-3-methylenehexanoic acid	C7H13NO2	29784-96-1		NO	metabo_info.php?molid=86954
93560	166.08	[M+Na]+	143.095	3	3beta,6beta-Dihydroxynortropane	C7H13NO2			NO	metabo_info.php?molid=93560
6589	166.08	[M+Na]+	143.095	3	1-Aminocyclohexanecarboxylic acid	C7H13NO2			YES	metabo_info.php?molid=6589
84924	657.36	[M+Na]+	634.372	3	Pandaroside B	C35H54O10			NO	metabo_info.php?molid=84924
2038	657.36	[M+Na]+	634.372	3	Digitoxigenin bisdigitoxoside	C35H54O10	16479-50-8		NO	metabo_info.php?molid=2038
68633	308.15	[M+H]+	307.142	4	Convoline	C16H21NO5	89783-61-9	C1085 5	NO	metabo_info.php?molid=68633
93249	308.15	[M+H]+	307.142	4	5-Amino-2,3-dihydro-6-(3-hydroxy-4-methoxy-1-oxobutyl)-2,2-dimethyl-4H-1-benzopyran-4-one	C16H21NO5	130767-47-4		NO	metabo_info.php?molid=93249
3186	308.15	[M+H]+	307.145	6	(±)-Desethylhydroxychloroquine	C16H22ClN3O	4298-15-1		NO	metabo_info.php?molid=3186
72295	308.15	[M+H]+	307.145	6	Tebuconazole	C16H22ClN3O	107534-96-3	C1848 9	YES	metabo_info.php?molid=72295
72231	377.04	[M+Na]+	354.049	6	Thiodicarb	C10H18N4O4S3	59669-26-0	C1842 3	YES	metabo_info.php?molid=72231
66420	377.04	[M+Na]+	354.054	6	WIN56291	C16H16Cl2N2O3		C0649 0	NO	metabo_info.php?molid=66420
92458	378.04	[M+Na]+	355.051	8	Quinoline yellow	C18H13NO5S	8004-92-0		NO	metabo_info.php?molid=92458
72505	378.04	[M+Na]+	355.055	0	Fluchloralin	C12H13ClF3N3O4	33245-39-5	C1873 0	NO	metabo_info.php?molid=72505

693	188.07	[M+H]+	187.059	9	1-(2-Hydroxyethyl)-2-hydroxymethyl-5-nitroimidazole	C6H9N3O4	NA		NO	metabo_info.php?molid=693
66434	188.07	[M+H]+	187.062	7	Deethylatrazine	C6H10ClN5	6190-65-4	C06559	NO	metabo_info.php?molid=66434
28	188.07	[M+Na]+	165.079	0	L-Phenylalanine	C9H11NO2	63-91-2	C00079	YES	metabo_info.php?molid=28
3934	188.07	[M+Na]+	165.079	0	benzocaine	C9H11NO2	#####	C07527	NO	metabo_info.php?molid=3934
63528	188.07	[M+Na]+	165.079	0	D-Phenylalanine	C9H11NO2	673-06-3	C02265	NO	metabo_info.php?molid=63528
65707	188.07	[M+Na]+	165.079	0	DL-Phenylalanine	C9H11NO2	150-30-1	C02057	YES	metabo_info.php?molid=65707
72021	188.07	[M+Na]+	165.079	0	Tricaine	C9H11NO2	582-33-2	C18090	NO	metabo_info.php?molid=72021
72521	188.07	[M+Na]+	165.079	0	Metolcarb	C9H11NO2	1129-41-5	C18747	NO	metabo_info.php?molid=72521
73234	188.07	[M+Na]+	165.079	0	4-Hydroxy-1-(3-pyridinyl)-1-butanone	C9H11NO2	59578-62-0	C19565	NO	metabo_info.php?molid=73234
73241	188.07	[M+Na]+	165.079	0	5-(3-Pyridyl)-2-hydroxytetrahydrofuran	C9H11NO2	53798-73-5	C19578	NO	metabo_info.php?molid=73241
89305	188.07	[M+Na]+	165.079	0	Gentiatibetine	C9H11NO2	26005-36-7		NO	metabo_info.php?molid=89305
89395	188.07	[M+Na]+	165.079	0	Ethyl 2-aminobenzoate	C9H11NO2	87-25-2		NO	metabo_info.php?molid=89395
89753	188.07	[M+Na]+	165.079	0	Methyl N-methylanthranilate	C9H11NO2	85-91-6		NO	metabo_info.php?molid=89753
855	188.07	[M+Na]+	165.079	0	N-(4-Hydroxyphenyl)propanamide	C9H11NO2	1693-37-4		NO	metabo_info.php?molid=855
2018	188.07	[M+Na]+	165.079	0	2-amino-4\'-hydroxy-Propiophenone	C9H11NO2	18259-42-2		NO	metabo_info.php?molid=2018
2183	188.07	[M+Na]+	165.079	0	2-Propylisonicotinic acid	C9H11NO2	57663-82-8		NO	metabo_info.php?molid=2183
5937	188.07	[M+Na]+	165.079	0	4-(3-Pyridyl)-butanoic acid	C9H11NO2			NO	metabo_info.php?molid=5937
48047	383.11	[M+H]+	382.105	3	Neobanone	C21H18O7			NO	metabo_info.php?molid=48047
48201	383.11	[M+H]+	382.105	3	Hildecarpidin	C21H18O7			NO	metabo_info.php?molid=48201
49036	383.11	[M+H]+	382.105	3	Velloquercetin 4\'-methyl ether	C21H18O7			NO	metabo_info.php?molid=49036

49943	383.11	[M+H]+	382.105	3	Artonin K	C21H18O7			NO	metabo_info.php?molid=49943
49958	383.11	[M+H]+	382.105	3	Artoindonesianin T	C21H18O7			NO	metabo_info.php?molid=49958
51130	383.11	[M+H]+	382.105	3	Velloquercetin 3-methyl ether	C21H18O7			NO	metabo_info.php?molid=51130
51141	383.11	[M+H]+	382.105	3	Sarothranol	C21H18O7			NO	metabo_info.php?molid=51141
51489	383.11	[M+H]+	382.105	3	8-Hydroxygalangin 3-methyl ether 8-angelate	C21H18O7			NO	metabo_info.php?molid=51489
51490	383.11	[M+H]+	382.105	3	3,5,7-Trihydroxy-8-methoxyflavone 3-angelate	C21H18O7			NO	metabo_info.php?molid=51490
51493	383.11	[M+H]+	382.105	3	5,7,8-Trihydroxy-3-methoxyflavone 8-((E)-2-methylbut-2-enoate)	C21H18O7			NO	metabo_info.php?molid=51493
53182	383.11	[M+H]+	382.105	3	Lisetin	C21H18O7			NO	metabo_info.php?molid=53182
63798	383.11	[M+H]+	382.105	3	12-Deoxyaklanonic acid	C21H18O7		C12420	NO	metabo_info.php?molid=63798
68373	383.11	[M+H]+	382.105	3	Austrobailignan 1	C21H18O7	55955-07-2	C10546	NO	metabo_info.php?molid=68373
89187	383.11	[M+H]+	382.105	3	Mollicellin A	C21H18O7	68455-06-1		NO	metabo_info.php?molid=89187
89188	383.11	[M+H]+	382.105	3	Mollicellin B	C21H18O7	68455-07-2		NO	metabo_info.php?molid=89188
41192	383.11	[M+Na]+	360.121	2	Elephantopin	C19H20O7		C09403	NO	metabo_info.php?molid=41192
47303	383.11	[M+Na]+	360.121	2	9,10-Dihydro-8-hydroxy-10-methyl-8H-pyrano[2,3-h]epicatechin	C19H20O7			NO	metabo_info.php?molid=47303
47304	383.11	[M+Na]+	360.121	2	Pyranochromene	C19H20O7			NO	metabo_info.php?molid=47304
47369	383.11	[M+Na]+	360.121	2	Elaeocyanidin	C19H20O7			NO	metabo_info.php?molid=47369
48176	383.11	[M+Na]+	360.121	2	8-Hydroxy-3,4,9,10-tetramethoxypterocarpan	C19H20O7			NO	metabo_info.php?molid=48176
48299	383.11	[M+Na]+	360.121	2	Abruquinone A	C19H20O7			NO	metabo_info.php?molid=48299
52167	383.11	[M+Na]+	360.121	2	2\',5\'-Dihydroxy-4,3\',4\',6\'-tetramethoxychalcone	C19H20O7			NO	metabo_info.php?molid=52167
52168	383.11	[M+Na]+	360.121	2	4,3\'-Hydroxy-2\',4\',5\',6\'-methoxychalcone	C19H20O7			NO	metabo_info.php?molid=52168

52940	383.11	[M+Na]+	360.121	2	5,7,3\'-Trihydroxy-4\',5\'-dimethoxy-6,8-di-C-methylflavanone	C19H20O7			NO	metabo_info.php?molid=52940
53016	383.11	[M+Na]+	360.121	2	Heteroflavanone A	C19H20O7			NO	metabo_info.php?molid=53016
53069	383.11	[M+Na]+	360.121	2	5-Hydroxy-7,3\',4\',5\'-tetramethoxyflavanone	C19H20O7			NO	metabo_info.php?molid=53069
53105	383.11	[M+Na]+	360.121	2	Agestricin C	C19H20O7			NO	metabo_info.php?molid=53105
53108	383.11	[M+Na]+	360.121	2	5-Hydroxy-6,7,3\',4\'-tetramethoxyflavanone	C19H20O7			NO	metabo_info.php?molid=53108
53109	383.11	[M+Na]+	360.121	2	Agecorynin E	C19H20O7			NO	metabo_info.php?molid=53109
53162	383.11	[M+Na]+	360.121	2	5-Hydroxy-6,7,8,4\'-tetramethoxyflavanone	C19H20O7			NO	metabo_info.php?molid=53162
67724	383.11	[M+Na]+	360.121	2	Goyazensolide	C19H20O7	60066-35-5	C09467	NO	metabo_info.php?molid=67724
67818	383.11	[M+Na]+	360.121	2	Vernodalin	C19H20O7	21871-10-3	C09576	NO	metabo_info.php?molid=67818
70442	383.11	[M+Na]+	360.121	2	2,3-Dihydro-2-(4-hydroxyphenyl)-5,6,7,8-tetramethoxy-4H-1-benzopyran-4-one	C19H20O7	70460-59-2	C14940	NO	metabo_info.php?molid=70442
90406	383.11	[M+Na]+	360.121	2	Gibberellin A59	C19H20O7	78333-20-7		NO	metabo_info.php?molid=90406
95618	383.11	[M+Na]+	360.121	2	Edulisin IV	C19H20O7	158446-38-9		NO	metabo_info.php?molid=95618
909	310.13	[M+H]+	309.119	0	Ketotifen	C19H19NOS	34580-13-7		YES	metabo_info.php?molid=909
2897	310.13	[M+H]+	309.121	7	Tranylcypromine glucuronide	C15H19NO6			NO	metabo_info.php?molid=2897
2261	600.28	[M+H]+	599.274	9	8\'-Hydroxydihydroergotamine	C33H37N5O6	90650-44-5		NO	metabo_info.php?molid=2261
40326	312.12	[M+H]+	311.113	6	PC(3:1(2E)/0:0)[U]	C11H22NO7P			NO	metabo_info.php?molid=40326
40354	312.12	[M+H]+	311.113	6	PC(0:0/3:1(2E))	C11H22NO7P			NO	metabo_info.php?molid=40354
67600	312.12	[M+H]+	311.116	0	Actinodaphnine	C18H17NO4	517-69-1	C09322	NO	metabo_info.php?molid=67600

67661	312.12	[M+H]+	311.116	0	Cularicine	C18H17NO4	#####	C09398	NO	metabo_info.php?molid=67661
68444	312.12	[M+H]+	311.116	0	Acronidine	C18H17NO4	518-68-3	C10629	NO	metabo_info.php?molid=68444
86806	312.12	[M+H]+	311.116	0	Launobine	C18H17NO4	20497-21-6		NO	metabo_info.php?molid=86806
89205	312.12	[M+H]+	311.116	0	Norisodomesticine	C18H17NO4	80151-84-4		NO	metabo_info.php?molid=89205
89206	312.12	[M+H]+	311.116	0	(S)-Nandigerine	C18H17NO4	31520-97-5		NO	metabo_info.php?molid=89206
89969	312.12	[M+H]+	311.116	0	(+)-Norushinsunine N-oxide	C18H17NO4			NO	metabo_info.php?molid=89969
91512	312.12	[M+H]+	311.116	0	Annocherine B	C18H17NO4			NO	metabo_info.php?molid=91512
1904	312.12	[M+Na]+	289.131	6	Benzoylecgonine	C16H19NO4	519-09-5	C10847	NO	metabo_info.php?molid=1904
68462	312.12	[M+Na]+	289.131	6	Balfourodine	C16H19NO4	484-61-7	C10647	NO	metabo_info.php?molid=68462
69340	312.12	[M+Na]+	289.131	6	Zephyranthine	C16H19NO4	2030-55-9	C12170	NO	metabo_info.php?molid=69340
69354	312.12	[M+Na]+	289.131	6	Pseudolycorine	C16H19NO4	29429-03-6	C12187	NO	metabo_info.php?molid=69354
1401	312.12	[M+Na]+	289.131	6	7,8-Dihydro-14-hydroxynormorphine	C16H19NO4	58477-92-2		NO	metabo_info.php?molid=1401
1905	312.12	[M+Na]+	289.131	6	Norcocaine	C16H19NO4	18717-72-1		NO	metabo_info.php?molid=1905
85586	312.12	[M+Na]+	289.135	3	Chloropyramine	C16H20ClN3	59-32-5		NO	metabo_info.php?molid=85586
45230	281.09	[M+H]+	280.085	0	AG-494	C16H12N2O3	133550-35-3		YES	metabo_info.php?molid=45230
71489	281.09	[M+H]+	280.085	0	Methyl nigakinone	C16H12N2O3	18110-87-7	C16996	NO	metabo_info.php?molid=71489
2834	281.09	[M+Na]+	258.104	3	Hydroxythiopental	C11H18N2O3S	104328-21-4		NO	metabo_info.php?molid=2834
38963	1069.9	[M+Na]+	1046.92	4	TG(22:0/22:3(10Z,13Z,16Z)/22:3(10Z,13Z,16Z))[iso3]	C69H122O6			NO	metabo_info.php?molid=38963
39010	1069.9	[M+Na]+	1046.92	4	TG(22:1(13Z)/22:3(10Z,13Z,16Z)/22:2(13Z,16Z))[iso6]	C69H122O6			NO	metabo_info.php?molid=39010

39014	1069.9	[M+Na]+	1046.92	4	TG(22:1(13Z)/22:1(13Z)/ 22:4(7Z,10Z,13Z,16Z))[is	C69H122O6			NO	metabo_info.php?molid=39014
39019	1069.9	[M+Na]+	1046.92	4	TG(22:0/22:1(13Z)/22:5(7 Z,10Z,13Z,16Z,19Z))[iso6	C69H122O6			NO	metabo_info.php?molid=39019
39026	1069.9	[M+Na]+	1046.92	4	TG(22:0/22:0/22:6(4Z,7Z, 10Z,13Z,16Z,19Z))[iso3]	C69H122O6			NO	metabo_info.php?molid=39026
39030	1069.9	[M+Na]+	1046.92	4	TG(22:0/22:2(13Z,16Z)/2 2:4(7Z,10Z,13Z,16Z))[iso	C69H122O6			NO	metabo_info.php?molid=39030
39057	1069.9	[M+Na]+	1046.92	4	TG(22:2(13Z,16Z)/22:2(1 3Z,16Z)/22:2(13Z,16Z))	C69H122O6			NO	metabo_info.php?molid=39057
99024	1069.9	[M+Na]+	1046.92	4	TG(22:1(11Z)/22:1(11Z)/ 22:4(7Z,10Z,13Z,16Z))[is	C69H122O6			NO	metabo_info.php?molid=99024
1E+05	1069.9	[M+Na]+	1046.92	4	TG(22:0/22:1(11Z)/22:5(7 Z,10Z,13Z,16Z,19Z))[iso6	C69H122O6			NO	metabo_info.php?molid=102714
1E+05	1069.9	[M+Na]+	1046.92	4	TG(22:1(11Z)/22:2(13Z,1 6Z)/22:3(10Z,13Z,16Z))[is	C69H122O6			NO	metabo_info.php?molid=102717
82180	703.57	[M+H]+	702.556	5	PA(O-18:0/19:1(9Z))	C40H79O7P			NO	metabo_info.php?molid=82180
82196	703.57	[M+H]+	702.556	5	PA(O-20:0/17:1(9Z))	C40H79O7P			NO	metabo_info.php?molid=82196
82263	703.57	[M+H]+	702.556	5	PA(P-16:0/21:0)	C40H79O7P			NO	metabo_info.php?molid=82263
82284	703.57	[M+H]+	702.556	5	PA(P-18:0/19:0)	C40H79O7P			NO	metabo_info.php?molid=82284
82304	703.57	[M+H]+	702.556	5	PA(P-20:0/17:0)	C40H79O7P			NO	metabo_info.php?molid=82304
69711	460.26	[M+Na]+	437.27	8	ORG 20599	C25H40ClNO3		C1380 4	NO	metabo_info.php?molid=69711
1799	351.22	[M+H]+	350.221	9	Perindopril lactam	C19H30N2O4	129970-98-5		NO	metabo_info.php?molid=1799
34827	329.24	[M+Na]+	306.256	6	5,8,11-eicosatrienoic acid	C20H34O2			NO	metabo_info.php?molid=34827
34829	329.24	[M+Na]+	306.256	6	11,14,17-eicosatrienoic acid	C20H34O2			NO	metabo_info.php?molid=34829
34830	329.24	[M+Na]+	306.256	6	Podocarpic acid	C20H34O2			NO	metabo_info.php?molid=34830
35043	329.24	[M+Na]+	306.256	6	2E,4E,8Z-eicosatrienoic acid	C20H34O2			NO	metabo_info.php?molid=35043
35044	329.24	[M+Na]+	306.256	6	5(Z),11(Z),14(Z)-Eicosatrienoic acid	C20H34O2	7019-85-4		YES	metabo_info.php?molid=35044
35045	329.24	[M+Na]+	306.256	6	5(Z),8(Z),11(Z)-Eicosatrienoic Acid	C20H34O2	20590-32-3		YES	metabo_info.php?molid=35045
35046	329.24	[M+Na]+	306.256	6	7,10,13-Eicosatrienoic acid	C20H34O2			NO	metabo_info.php?molid=35046

35047	329.24	[M+Na]+	306.256	6	7,11,14-Eicosatrienoic acid	C20H34O2			NO	metabo_info.php?molid=35047
35048	329.24	[M+Na]+	306.256	6	7Z,10Z,13Z-eicosatrienoic acid	C20H34O2			NO	metabo_info.php?molid=35048
35049	329.24	[M+Na]+	306.256	6	7Z,11Z,14E-eicosatrienoic acid	C20H34O2			NO	metabo_info.php?molid=35049
35050	329.24	[M+Na]+	306.256	6	7Z,11Z,14Z-eicosatrienoic acid	C20H34O2			NO	metabo_info.php?molid=35050
35051	329.24	[M+Na]+	306.256	6	8Z,12E,14Z-eicosatrienoic acid	C20H34O2	NA		NO	metabo_info.php?molid=35051
259	329.24	[M+Na]+	306.256	6	Eicosatrienoic acid	C20H34O2	27070-56-0		NO	metabo_info.php?molid=259
43737	329.24	[M+Na]+	306.256	6	LARIXOL	C20H34O2			NO	metabo_info.php?molid=43737
45006	329.24	[M+Na]+	306.256	6	Linolenic Acid ethyl ester	C20H34O2	1191-41-9		YES	metabo_info.php?molid=45006
45018	329.24	[M+Na]+	306.256	6	9(Z),11(E),13(E)-Octadecatrienoic Acid ethyl ester	C20H34O2	42021-86-3		YES	metabo_info.php?molid=45018
34828	329.24	[M+Na]+	306.256	6	Dihomo-γ-Linolenic Acid	C20H34O2	1783-84-2	C03242	YES	metabo_info.php?molid=34828
35042	329.24	[M+Na]+	306.256	6	11(Z),14(Z),17(Z)-Eicosatrienoic Acid	C20H34O2	17046-59-2	C16522	YES	metabo_info.php?molid=35042
45143	329.24	[M+Na]+	306.256	6	Pinolenic Acid ethyl ester	C20H34O2	493015-74-0		YES	metabo_info.php?molid=45143
45191	329.24	[M+Na]+	306.256	6	5(Z),8(Z),14(Z)-Eicosatrienoic Acid	C20H34O2	90105-02-5		YES	metabo_info.php?molid=45191
45776	329.24	[M+Na]+	306.256	6	(5Z,9E,14Z)-icosa-5,9,14-trienoic acid	C20H34O2			NO	metabo_info.php?molid=45776
45901	329.24	[M+Na]+	306.256	6	Oncobic acid	C20H34O2			NO	metabo_info.php?molid=45901
46394	329.24	[M+Na]+	306.256	6	3Z,6Z,9Z-Octadecatrienyl acetate	C20H34O2			NO	metabo_info.php?molid=46394
46395	329.24	[M+Na]+	306.256	6	9Z,12Z,15Z-Octadecatrienyl acetate	C20H34O2			NO	metabo_info.php?molid=46395
53579	329.24	[M+Na]+	306.256	6	plaunotol	C20H34O2		C1327 3	NO	metabo_info.php?molid=53579

67404	329.24	[M+Na]+	306.256	6	2,7,11-Cembratrien-4,6-diol	C20H34O2	57605-80-8	C09072	NO	metabo_info.php?molid=67404
70385	329.24	[M+Na]+	306.256	6	2alpha-Methyl-5alpha-androstane-3alpha,17beta-diol	C20H34O2		C14882	NO	metabo_info.php?molid=70385
70552	329.24	[M+Na]+	306.256	6	2alpha-Methyl-5alpha-androstane-3beta,17beta-diol	C20H34O2		C15058	NO	metabo_info.php?molid=70552
70847	329.24	[M+Na]+	306.256	6	17alpha-Methyl-5alpha-androstane-3alpha,17beta-diol	C20H34O2		C15376	NO	metabo_info.php?molid=70847
70868	329.24	[M+Na]+	306.256	6	17-Methyl-5alpha-androstane-11beta,17beta-diol	C20H34O2		C15399	NO	metabo_info.php?molid=70868
73667	329.24	[M+Na]+	306.256	6	18-methyl-8Z,11Z,14Z-nonadecatrienoic acid	C20H34O2			NO	metabo_info.php?molid=73667
73853	329.24	[M+Na]+	306.256	6	5, 8, 11-icosatrienoic acid; C20:3n-9,12,15	C20H34O2			NO	metabo_info.php?molid=73853
73854	329.24	[M+Na]+	306.256	6	11, 14, 17-icosatrienoic acid; C20:3n-3,6,9	C20H34O2			NO	metabo_info.php?molid=73854
74048	329.24	[M+Na]+	306.256	6	C20:3n-12,16,18	C20H34O2			NO	metabo_info.php?molid=74048
74049	329.24	[M+Na]+	306.256	6	Sciadonic acid	C20H34O2			NO	metabo_info.php?molid=74049
74050	329.24	[M+Na]+	306.256	6	7,10,13-Eicosatrienoic acid	C20H34O2			NO	metabo_info.php?molid=74050
74051	329.24	[M+Na]+	306.256	6	7,11,14-Eicosatrienoic acid	C20H34O2			NO	metabo_info.php?molid=74051
74052	329.24	[M+Na]+	306.256	6	7Z,10Z,13Z-eicosatrienoic acid	C20H34O2			NO	metabo_info.php?molid=74052
74053	329.24	[M+Na]+	306.256	6	7Z,11Z,14E-eicosatrienoic acid	C20H34O2	81861-75-8		NO	metabo_info.php?molid=74053
74054	329.24	[M+Na]+	306.256	6	7Z,11Z,14Z-eicosatrienoic acid	C20H34O2			NO	metabo_info.php?molid=74054
74055	329.24	[M+Na]+	306.256	6	C20:3n-6,8,12	C20H34O2			NO	metabo_info.php?molid=74055
74347	329.24	[M+Na]+	306.256	6	eicosa-5Z,8Z,14Z-trienoic acid	C20H34O2			NO	metabo_info.php?molid=74347
74399	329.24	[M+Na]+	306.256	6	20:3(5Z,13Z,16Z)	C20H34O2			NO	metabo_info.php?molid=74399

91848	329.24	[M+Na]+	306.256	6	Sagittariol	C20H34O2	56497-92-8		NO	metabo_info.php?molid=91848
96550	329.24	[M+Na]+	306.256	6	γ-Linolenic Acid ethyl ester	C20H34O2	31450-14-3		YES	metabo_info.php?molid=96550
97248	329.24	[M+Na]+	306.256	6	(E,E)-3,7,11-Trimethyl-2,6,10-dodecatrienyl 3-methylbutanoate	C20H34O2			NO	metabo_info.php?molid=97248
97250	329.24	[M+Na]+	306.256	6	(E,E)-3,7,11-Trimethyl-2,6,10-dodecatrienyl pentanoate	C20H34O2			NO	metabo_info.php?molid=97250
3E+05	329.24	[M+Na]+	306.256	6	Isoincensole	C20H34O2			YES	metabo_info.php?molid=265077
3E+05	329.24	[M+Na]+	306.256	6	Incensole	C20H34O2			YES	metabo_info.php?molid=265078
89303	178.08	[M+H]+	177.079	9	Plantagonine	C10H11NO2	21857-97-6		NO	metabo_info.php?molid=89303
92517	178.08	[M+H]+	177.079	9	2-Propenyl 2-aminobenzoate	C10H11NO2	7493-63-2		NO	metabo_info.php?molid=92517
94586	178.08	[M+H]+	177.079	9	5-[2H-Pyrrol-4-(3H)-ylidenemethyl]-2-furanmethanol	C10H11NO2			NO	metabo_info.php?molid=94586
96729	178.08	[M+H]+	177.079	9	MDAI	C10H11NO2	155344-90-4		YES	metabo_info.php?molid=96729
44702	178.08	[M+Na]+	155.095	4	2-Amino-2-Norbornanecarboxylic acid	C8H13NO2	20448-79-7		NO	metabo_info.php?molid=44702
43570	178.08	[M+Na]+	155.095	4	ARECOLINE	C8H13NO2	300-08-3	C10129	YES	metabo_info.php?molid=43570
43998	178.08	[M+Na]+	155.095	4	SCOPOLINE	C8H13NO2	487-27-4	C10866	YES	metabo_info.php?molid=43998
64442	178.08	[M+Na]+	155.095	4	Retronecine	C8H13NO2	480-85-3	C06177	NO	metabo_info.php?molid=64442
43431	331.26	[M+Na]+	308.272	6	Eicosa-5Z,8Z-dienoic acid (20:2, n-12)	C20H36O2			YES	metabo_info.php?molid=43431
35037	331.26	[M+Na]+	308.272	6	11Z,15Z-eicosadienoic acid	C20H36O2			NO	metabo_info.php?molid=35037
35038	331.26	[M+Na]+	308.272	6	5,11-Eicosadienoic acid	C20H36O2			NO	metabo_info.php?molid=35038
35039	331.26	[M+Na]+	308.272	6	7,13-Eicosadienoic acid	C20H36O2			NO	metabo_info.php?molid=35039

35040	331.26	[M+Na]+	308.272	6	8,11-Eicosadienoic acid	C20H36O2			NO	metabo_info.php?molid=35040
35041	331.26	[M+Na]+	308.272	6	8Z,11Z-eicosadienoic acid	C20H36O2			NO	metabo_info.php?molid=35041
45005	331.26	[M+Na]+	308.272	6	Linoleic Acid ethyl ester	C20H36O2	544-35-4		YES	metabo_info.php?molid=45005
24088	331.26	[M+Na]+	308.272	6	11,14-trans-Eicosadienoic acid	C20H36O2			YES	metabo_info.php?molid=24088
46383	331.26	[M+Na]+	308.272	6	3E,13E-Octadecadienyl acetate	C20H36O2			NO	metabo_info.php?molid=46383
46384	331.26	[M+Na]+	308.272	6	2E,13Z-Octadecadienyl acetate	C20H36O2			NO	metabo_info.php?molid=46384
46385	331.26	[M+Na]+	308.272	6	3E,13Z-Octadecadienyl acetate	C20H36O2			NO	metabo_info.php?molid=46385
46389	331.26	[M+Na]+	308.272	6	2Z,13E-Octadecadienyl acetate	C20H36O2			NO	metabo_info.php?molid=46389
46390	331.26	[M+Na]+	308.272	6	3Z,13E-Octadecadienyl acetate	C20H36O2			NO	metabo_info.php?molid=46390
46391	331.26	[M+Na]+	308.272	6	2Z,13Z-Octadecadienyl acetate	C20H36O2			NO	metabo_info.php?molid=46391
46392	331.26	[M+Na]+	308.272	6	3Z,13Z-Octadecadienyl acetate	C20H36O2			NO	metabo_info.php?molid=46392
46393	331.26	[M+Na]+	308.272	6	9Z,12Z-Octadecadienyl acetate	C20H36O2			NO	metabo_info.php?molid=46393
53595	331.26	[M+Na]+	308.272	6	sclareol	C20H36O2		C09183	YES	metabo_info.php?molid=53595
62964	331.26	[M+Na]+	308.272	6	11(Z),14(Z)-Eicosadienoic Acid	C20H36O2	2091-39-6	C16525	YES	metabo_info.php?molid=62964
64791	331.26	[M+Na]+	308.272	6	8(Z),14(Z)-Eicosadienoic Acid	C20H36O2	135498-07-6		YES	metabo_info.php?molid=64791
73840	331.26	[M+Na]+	308.272	6	11, 14-icosadienoic acid; C20:2n-6,9	C20H36O2			NO	metabo_info.php?molid=73840
74043	331.26	[M+Na]+	308.272	6	C20:2n-5,9	C20H36O2			NO	metabo_info.php?molid=74043
74044	331.26	[M+Na]+	308.272	6	C20:2n-9,15	C20H36O2			NO	metabo_info.php?molid=74044
74045	331.26	[M+Na]+	308.272	6	C20:2n-7,13	C20H36O2			NO	metabo_info.php?molid=74045
74046	331.26	[M+Na]+	308.272	6	8,11-Eicosadienoic acid	C20H36O2			NO	metabo_info.php?molid=74046
74047	331.26	[M+Na]+	308.272	6	8Z,11Z-eicosadienoic acid	C20H36O2			NO	metabo_info.php?molid=74047

74395	331.26	[M+Na]+	308.272	6	20:2(7Z,14Z)	C20H36O2			NO	metabo_info.php?molid=74395
74396	331.26	[M+Na]+	308.272	6	20:2(5Z,13Z)	C20H36O2			NO	metabo_info.php?molid=74396
74401	331.26	[M+Na]+	308.272	6	20:2(5Z,11Z)	C20H36O2			NO	metabo_info.php?molid=74401
74403	331.26	[M+Na]+	308.272	6	20:2(5Z,15Z)	C20H36O2			NO	metabo_info.php?molid=74403
74433	331.26	[M+Na]+	308.272	6	20:2(6,11)	C20H36O2			NO	metabo_info.php?molid=74433
74435	331.26	[M+Na]+	308.272	6	20:2(5Z,9Z)	C20H36O2			NO	metabo_info.php?molid=74435
96465	331.26	[M+Na]+	308.272	6	5(Z),14(Z)-Eicosadienoic Acid	C20H36O2	122055-58-7		YES	metabo_info.php?molid=96465
97305	331.26	[M+Na]+	308.272	6	(E)-3,7-Dimethyl-2,6-octadienyl decanoate	C20H36O2			NO	metabo_info.php?molid=97305
97317	331.26	[M+Na]+	308.272	6	(Z)-3,7-Dimethyl-2,6-octadienyl decanoate	C20H36O2			NO	metabo_info.php?molid=97317
72401	327.22	[M+Na]+	304.238	6	Sodium oleate	C18H33NaO2	143-19-1	C18601	NO	metabo_info.php?molid=72401
72401	305.24	[M+H]+	304.238	6	Sodium oleate	C18H33NaO2	143-19-1	C18601	NO	metabo_info.php?molid=72401
34748	305.24	[M+Na]+	282.256	6	2Z-octadecenoic acid	C18H34O2			NO	metabo_info.php?molid=34748
34749	305.24	[M+Na]+	282.256	6	trans-2-oleic acid	C18H34O2			NO	metabo_info.php?molid=34749
34750	305.24	[M+Na]+	282.256	6	3-octadecylenic acid	C18H34O2			NO	metabo_info.php?molid=34750
34751	305.24	[M+Na]+	282.256	6	4-octadecylenic acid	C18H34O2			NO	metabo_info.php?molid=34751
34752	305.24	[M+Na]+	282.256	6	5-octadecylenic acid	C18H34O2			NO	metabo_info.php?molid=34752
34753	305.24	[M+Na]+	282.256	6	Petroselaidic acid	C18H34O2			NO	metabo_info.php?molid=34753
34754	305.24	[M+Na]+	282.256	6	7Z-octadecenoic acid	C18H34O2			NO	metabo_info.php?molid=34754
34755	305.24	[M+Na]+	282.256	6	7E-octadecenoic acid	C18H34O2			NO	metabo_info.php?molid=34755
34756	305.24	[M+Na]+	282.256	6	cis-8-oleic acid	C18H34O2			NO	metabo_info.php?molid=34756
34757	305.24	[M+Na]+	282.256	6	trans-8-elaidic acid	C18H34O2			NO	metabo_info.php?molid=34757
34758	305.24	[M+Na]+	282.256	6	cis-10-oleic acid	C18H34O2			NO	metabo_info.php?molid=34758
34759	305.24	[M+Na]+	282.256	6	10E-octadecenoic acid	C18H34O2			NO	metabo_info.php?molid=34759
34760	305.24	[M+Na]+	282.256	6	cis-12-oleic acid	C18H34O2			NO	metabo_info.php?molid=34760
34761	305.24	[M+Na]+	282.256	6	trans-12-elaidic acid	C18H34O2			NO	metabo_info.php?molid=34761
34762	305.24	[M+Na]+	282.256	6	15E-octadecenoic acid	C18H34O2			NO	metabo_info.php?molid=34762
34763	305.24	[M+Na]+	282.256	6	16E-octadecenoic acid	C18H34O2			NO	metabo_info.php?molid=34763
34954	305.24	[M+Na]+	282.256	6	13Z-octadecenoic acid	C18H34O2			NO	metabo_info.php?molid=34954
34955	305.24	[M+Na]+	282.256	6	15Z-octadecenoic acid	C18H34O2			NO	metabo_info.php?molid=34955

34956	305.24	[M+Na]+	282.256	6	16Z-octadecenoic acid	C18H34O2			NO	metabo_info.php?molid=34956
34957	305.24	[M+Na]+	282.256	6	17-octadecenoic acid	C18H34O2			NO	metabo_info.php?molid=34957
34958	305.24	[M+Na]+	282.256	6	3Z-octadecenoic acid	C18H34O2			NO	metabo_info.php?molid=34958
34959	305.24	[M+Na]+	282.256	6	4Z-octadecenoic acid	C18H34O2			NO	metabo_info.php?molid=34959
34960	305.24	[M+Na]+	282.256	6	5Z-octadecenoic acid	C18H34O2			NO	metabo_info.php?molid=34960
45010	305.24	[M+Na]+	282.256	6	Palmitoleic Acid ethyl ester	C18H34O2	56219-10-4		YES	metabo_info.php?molid=45010
190	305.24	[M+Na]+	282.256	6	Oleic Acid	C18H34O2	112-80-1	C00712	YES	metabo_info.php?molid=190
3406	305.24	[M+Na]+	282.256	6	Elaidic Acid	C18H34O2	112-79-8	C01712	YES	metabo_info.php?molid=3406
3407	305.24	[M+Na]+	282.256	6	Vaccenic acid	C18H34O2	693-72-1	C08367	YES	metabo_info.php?molid=3407
3552	305.24	[M+Na]+	282.256	6	cis-vaccenic acid	C18H34O2	506-17-2	C08367	YES	metabo_info.php?molid=3552
3553	305.24	[M+Na]+	282.256	6	Petroselinic acid	C18H34O2	593-39-5	C08363	YES	metabo_info.php?molid=3553
45890	305.24	[M+Na]+	282.256	6	11-Cycloheptylundecanoic acid	C18H34O2		C12103	NO	metabo_info.php?molid=45890
46342	305.24	[M+Na]+	282.256	6	11E-Hexadecenyl acetate	C18H34O2			NO	metabo_info.php?molid=46342
46343	305.24	[M+Na]+	282.256	6	5E-Hexadecenyl acetate	C18H34O2			NO	metabo_info.php?molid=46343
46344	305.24	[M+Na]+	282.256	6	6E-Hexadecenyl acetate	C18H34O2			NO	metabo_info.php?molid=46344
46345	305.24	[M+Na]+	282.256	6	8E-Hexadecenyl acetate	C18H34O2			NO	metabo_info.php?molid=46345
46346	305.24	[M+Na]+	282.256	6	9E-Hexadecenyl acetate	C18H34O2			NO	metabo_info.php?molid=46346
46360	305.24	[M+Na]+	282.256	6	10Z-Hexadecenyl acetate	C18H34O2			NO	metabo_info.php?molid=46360
46361	305.24	[M+Na]+	282.256	6	11Z-Hexadecenyl acetate	C18H34O2			NO	metabo_info.php?molid=46361
46362	305.24	[M+Na]+	282.256	6	12Z-Hexadecenyl acetate	C18H34O2			NO	metabo_info.php?molid=46362
46364	305.24	[M+Na]+	282.256	6	3Z-Hexadecenyl acetate	C18H34O2			NO	metabo_info.php?molid=46364
46365	305.24	[M+Na]+	282.256	6	5Z-Hexadecenyl acetate	C18H34O2			NO	metabo_info.php?molid=46365
46366	305.24	[M+Na]+	282.256	6	7Z-Hexadecenyl acetate	C18H34O2			NO	metabo_info.php?molid=46366
46367	305.24	[M+Na]+	282.256	6	9Z-Hexadecenyl acetate	C18H34O2			NO	metabo_info.php?molid=46367
46377	305.24	[M+Na]+	282.256	6	7-Hexadecenyl acetate	C18H34O2			NO	metabo_info.php?molid=46377
73652	305.24	[M+Na]+	282.256	6	2-methyl-16-heptadecenoic acid	C18H34O2			NO	metabo_info.php?molid=73652

73782	305.24	[M+Na]+	282.256	6	17:1(4)(16Me)	C18H34O2	NO	metabo_info.php?molid=73782
73783	305.24	[M+Na]+	282.256	6	17:1(6)(9Me)	C18H34O2	NO	metabo_info.php?molid=73783
73784	305.24	[M+Na]+	282.256	6	17:1(12)(7Me)	C18H34O2	NO	metabo_info.php?molid=73784
73804	305.24	[M+Na]+	282.256	6	cis-2-octadecenoic acid; C18:1n-16	C18H34O2	NO	metabo_info.php?molid=73804
73805	305.24	[M+Na]+	282.256	6	cis-7-octadecenoic acid; C18:1n-11	C18H34O2	NO	metabo_info.php?molid=73805
73806	305.24	[M+Na]+	282.256	6	trans-7-octadecenoic acid; C18:1n-11	C18H34O2	NO	metabo_info.php?molid=73806
73807	305.24	[M+Na]+	282.256	6	trans-10-octadecenoic acid; C18:1n-8	C18H34O2	NO	metabo_info.php?molid=73807
73808	305.24	[M+Na]+	282.256	6	trans-15-octadecenoic acid; C18:1n-3	C18H34O2	NO	metabo_info.php?molid=73808
73809	305.24	[M+Na]+	282.256	6	trans-16-octadecenoic acid; C18:1n-2	C18H34O2	NO	metabo_info.php?molid=73809
73963	305.24	[M+Na]+	282.256	6	C18:1n-5	C18H34O2	NO	metabo_info.php?molid=73963
73964	305.24	[M+Na]+	282.256	6	C18:1n-3	C18H34O2	NO	metabo_info.php?molid=73964
73965	305.24	[M+Na]+	282.256	6	C18:1n-2	C18H34O2	NO	metabo_info.php?molid=73965
73966	305.24	[M+Na]+	282.256	6	C18:1n-1	C18H34O2	NO	metabo_info.php?molid=73966
73967	305.24	[M+Na]+	282.256	6	C18:1n-15	C18H34O2	NO	metabo_info.php?molid=73967
73968	305.24	[M+Na]+	282.256	6	C18:1n-14	C18H34O2	NO	metabo_info.php?molid=73968
73969	305.24	[M+Na]+	282.256	6	C18:1n-13	C18H34O2	NO	metabo_info.php?molid=73969
74416	305.24	[M+Na]+	282.256	6	18:1(13E)	C18H34O2	NO	metabo_info.php?molid=74416
74417	305.24	[M+Na]+	282.256	6	18:1(14E)	C18H34O2	NO	metabo_info.php?molid=74417
74418	305.24	[M+Na]+	282.256	6	18:1(14Z)	C18H34O2	NO	metabo_info.php?molid=74418
74431	305.24	[M+Na]+	282.256	6	17:1(4)(15Me)	C18H34O2	NO	metabo_info.php?molid=74431
97217	305.24	[M+Na]+	282.256	6	ethyl 7Z-hexadecenoate	C18H34O2	NO	metabo_info.php?molid=97217
97218	305.24	[M+Na]+	282.256	6	formyl 14-methyl-8E-hexadecenoate	C18H34O2	NO	metabo_info.php?molid=97218
97220	305.24	[M+Na]+	282.256	6	formyl 14-methyl-8Z-hexadecenoate	C18H34O2	NO	metabo_info.php?molid=97220
97323	305.24	[M+Na]+	282.256	6	Ethyl 9-hexadecenoate	C18H34O2	NO	metabo_info.php?molid=97323
97409	305.24	[M+Na]+	282.256	6	18-Octadecanolide	C18H34O2	NO	metabo_info.php?molid=97409
1E+05	782.56	[M+H]+	781.547	6	PI-Cer(d18:0/16:0)	C40H80NO11P	NO	metabo_info.php?molid=103135

1E+05	782.56	[M+H]+	781.547	5	PI-Cer(d18:0/16:0)	C40H80NO11P			NO	metabo_info.php?molid=103135
1E+05	782.56	[M+H]+	781.547	5	PI-Cer(d18:0/16:0)	C40H80NO11P			NO	metabo_info.php?molid=103135
44825	301.14	[M+Na]+	278.152	3	α-CEHC	C16H22O4	NA		YES	metabo_info.php?molid=44825
53414	301.14	[M+Na]+	278.152	3	Emmotin A	C16H22O4			NO	metabo_info.php?molid=53414
53844	301.14	[M+Na]+	278.152	3	alhpa-tocopheronolactone	C16H22O4			NO	metabo_info.php?molid=53844
44802	301.14	[M+Na]+	278.152	3	Phthalic acid Mono-2-ethylhexyl Ester	C16H22O4	4376-20-9	C03343	NO	metabo_info.php?molid=44802
58055	301.14	[M+Na]+	278.152	3	Alpha-CEHC	C16H22O4	4072-32-6		NO	metabo_info.php?molid=58055
69881	301.14	[M+Na]+	278.152	3	Dibutyl phthalate	C16H22O4	84-74-2	C14214	NO	metabo_info.php?molid=69881
70692	301.14	[M+Na]+	278.152	3	Diisobutyl phthalate	C16H22O4	84-69-5	C15205	NO	metabo_info.php?molid=70692
2764	424.16	[M+Na]+	401.169	7	Terbutaline-1-glucuronide	C18H27NO9			NO	metabo_info.php?molid=2764
43893	424.16	[M+Na]+	401.175	7	MOXIFLOXACIN	C21H24FN3O4	354812-41-2		YES	metabo_info.php?molid=43893
66708	424.16	[M+Na]+	401.175	7	Moxifloxacin	C21H24FN3O4	151096-09-2	C07663	NO	metabo_info.php?molid=66708
53843	319.15	[M+Na]+	296.162	8	alhpa-tocopheronic acid	C16H24O5			NO	metabo_info.php?molid=53843
67878	319.15	[M+Na]+	296.162	8	Graphinone	C16H24O5	19683-98-8	C09674	NO	metabo_info.php?molid=67878
93953	319.15	[M+Na]+	296.162	8	Methyl dihydrophaseate	C16H24O5			NO	metabo_info.php?molid=93953
95934	319.15	[M+Na]+	296.162	8	3,8-Dihydroxy-6-methoxy-7(11)-eremophilen-12,8-olide	C16H24O5			NO	metabo_info.php?molid=95934
2295	319.15	[M+Na]+	296.162	8	Lactone of PGF-MUM	C16H24O5			NO	metabo_info.php?molid=2295
39317	760.58	[M+H]+	759.578	9	PC(16:0/18:1(11E))	C42H82NO8P			NO	metabo_info.php?molid=39317
39319	760.58	[M+H]+	759.578	9	PC(16:0/18:1(11Z))[U]	C42H82NO8P			NO	metabo_info.php?molid=39319
39320	760.58	[M+H]+	759.578	9	PC(16:0/18:1(6E))	C42H82NO8P			NO	metabo_info.php?molid=39320
39321	760.58	[M+H]+	759.578	9	PC(16:0/18:1(6Z))	C42H82NO8P			NO	metabo_info.php?molid=39321
39322	760.58	[M+H]+	759.578	9	PC(16:0/18:1(6Z))[U]	C42H82NO8P			NO	metabo_info.php?molid=39322
39323	760.58	[M+H]+	759.578	9	PC(16:0/18:1(9E))	C42H82NO8P			NO	metabo_info.php?molid=39323
39324	760.58	[M+H]+	759.578	9	PC(16:0/18:1(9E))[U]	C42H82NO8P			NO	metabo_info.php?molid=39324
39325	760.58	[M+H]+	759.578	9	PC(16:0/18:1(9Z))[S]	C42H82NO8P		C13875	NO	metabo_info.php?molid=39325
39326	760.58	[M+H]+	759.578	9	PC(16:0/18:1(9Z))[U]	C42H82NO8P			NO	metabo_info.php?molid=39326
39420	760.58	[M+H]+	759.578	9	PC(16:1(7Z)/18:0)[U]	C42H82NO8P			NO	metabo_info.php?molid=39420

39427	760.58	[M+H]+	759.578	9	PC(16:1(9Z)/18:0)[U]	C42H82NO8P		NO	metabo_info.php?molid=39427
39615	760.58	[M+H]+	759.578	9	PC(18:1(9E)/16:0)[U]	C42H82NO8P		NO	metabo_info.php?molid=39615
39626	760.58	[M+H]+	759.578	9	PC(18:1(9Z)/16:0)[U]	C42H82NO8P		NO	metabo_info.php?molid=39626
59329	760.58	[M+H]+	759.578	9	PC(14:0/20:1(11Z))	C42H82NO8P	[illegible]	NO	metabo_info.php?molid=59329
59361	760.58	[M+H]+	759.578	9	PC(14:1(9Z)/20:0)	C42H82NO8P	[illegible]	NO	metabo_info.php?molid=59361
59420	760.58	[M+H]+	759.578	9	PC(16:0/18:1(11Z))	C42H82NO8P	[illegible]	NO	metabo_info.php?molid=59420
59421	760.58	[M+H]+	759.578	9	PC(16:0/18:1(9Z))	C42H82NO8P	[illegible]	NO	metabo_info.php?molid=59421
59451	760.58	[M+H]+	759.578	9	PC(16:1(9Z)/18:0)	C42H82NO8P	[illegible]	NO	metabo_info.php?molid=59451
59483	760.58	[M+H]+	759.578	9	PC(18:0/16:1(9Z))	C42H82NO8P	[illegible]	NO	metabo_info.php?molid=59483
59514	760.58	[M+H]+	759.578	9	PC(18:1(11Z)/16:0)	C42H82NO8P	[illegible]	NO	metabo_info.php?molid=59514
59547	760.58	[M+H]+	759.578	9	PC(18:1(9Z)/16:0)	C42H82NO8P	[illegible]	NO	metabo_info.php?molid=59547
59709	760.58	[M+H]+	759.578	9	PC(20:0/14:1(9Z))	C42H82NO8P	[illegible]	NO	metabo_info.php?molid=59709
59741	760.58	[M+H]+	759.578	9	PC(20:1(11Z)/14:0)	C42H82NO8P	[illegible]	NO	metabo_info.php?molid=59741
60351	760.58	[M+H]+	759.578	9	PE(15:0/22:1(13Z))	C42H82NO8P	[illegible]	NO	metabo_info.php?molid=60351
60905	760.58	[M+H]+	759.578	9	PE(22:1(13Z)/15:0)	C42H82NO8P	[illegible]	NO	metabo_info.php?molid=60905
75615	760.58	[M+H]+	759.578	9	PC(12:0/22:1(11Z))	C42H82NO8P		NO	metabo_info.php?molid=75615
75699	760.58	[M+H]+	759.578	9	PC(15:0/19:1(9Z))	C42H82NO8P		NO	metabo_info.php?molid=75699
75728	760.58	[M+H]+	759.578	9	PC(15:1(9Z)/19:0)	C42H82NO8P		NO	metabo_info.php?molid=75728
75783	760.58	[M+H]+	759.578	9	PC(17:0/17:1(9Z))	C42H82NO8P		NO	metabo_info.php?molid=75783
75809	760.58	[M+H]+	759.578	9	PC(17:1(9Z)/17:0)	C42H82NO8P		NO	metabo_info.php?molid=75809
76012	760.58	[M+H]+	759.578	9	PC(19:0/15:1(9Z))	C42H82NO8P		NO	metabo_info.php?molid=76012
76040	760.58	[M+H]+	759.578	9	PC(19:1(9Z)/15:0)	C42H82NO8P		NO	metabo_info.php?molid=76040
76284	760.58	[M+H]+	759.578	9	PC(22:1(11Z)/12:0)	C42H82NO8P		NO	metabo_info.php?molid=76284
76705	760.58	[M+H]+	759.578	9	PE(15:0/22:1(11Z))	C42H82NO8P		NO	metabo_info.php?molid=76705
76735	760.58	[M+H]+	759.578	9	PE(15:1(9Z)/22:0)	C42H82NO8P		NO	metabo_info.php?molid=76735
76769	760.58	[M+H]+	759.578	9	PE(16:1(9Z)/21:0)	C42H82NO8P		NO	metabo_info.php?molid=76769
76787	760.58	[M+H]+	759.578	9	PE(17:0/20:1(11Z))	C42H82NO8P		NO	metabo_info.php?molid=76787
76814	760.58	[M+H]+	759.578	9	PE(17:1(9Z)/20:0)	C42H82NO8P		NO	metabo_info.php?molid=76814
76866	760.58	[M+H]+	759.578	9	PE(18:0/19:1(9Z))	C42H82NO8P		NO	metabo_info.php?molid=76866
76880	760.58	[M+H]+	759.578	9	PE(18:1(9Z)/19:0)	C42H82NO8P		NO	metabo_info.php?molid=76880
77010	760.58	[M+H]+	759.578	9	PE(19:0/18:1(9Z))	C42H82NO8P		NO	metabo_info.php?molid=77010
77039	760.58	[M+H]+	759.578	9	PE(19:1(9Z)/18:0)	C42H82NO8P		NO	metabo_info.php?molid=77039
77064	760.58	[M+H]+	759.578	9	PE(20:0/17:1(9Z))	C42H82NO8P		NO	metabo_info.php?molid=77064
77084	760.58	[M+H]+	759.578	9	PE(20:1(11Z)/17:0)	C42H82NO8P		NO	metabo_info.php?molid=77084

77230	760.58	[M+H]+	759.578	9	PE(21:0/16:1(9Z))	C42H82NO8P			NO	metabo_info.php?molid=77230
77253	760.58	[M+H]+	759.578	9	PE(22:0/15:1(9Z))	C42H82NO8P			NO	metabo_info.php?molid=77253
77279	760.58	[M+H]+	759.578	9	PE(22:1(11Z)/15:0)	C42H82NO8P			NO	metabo_info.php?molid=77279
71650	512.21	[M+Na]+	489.214	6	OA-6129 E	C21H35N3O8S	85414-26-2	C17376	NO	metabo_info.php?molid=71650
39317	760.58	[M+H]+	759.578	9	PC(16:0/18:1(11E))	C42H82NO8P			NO	metabo_info.php?molid=39317
39319	760.58	[M+H]+	759.578	9	PC(16:0/18:1(11Z))[U]	C42H82NO8P			NO	metabo_info.php?molid=39319
39320	760.58	[M+H]+	759.578	9	PC(16:0/18:1(6E))	C42H82NO8P			NO	metabo_info.php?molid=39320
39321	760.58	[M+H]+	759.578	9	PC(16:0/18:1(6Z))	C42H82NO8P			NO	metabo_info.php?molid=39321
39322	760.58	[M+H]+	759.578	9	PC(16:0/18:1(6Z))[U]	C42H82NO8P			NO	metabo_info.php?molid=39322
39323	760.58	[M+H]+	759.578	9	PC(16:0/18:1(9E))	C42H82NO8P			NO	metabo_info.php?molid=39323
39324	760.58	[M+H]+	759.578	9	PC(16:0/18:1(9E))[U]	C42H82NO8P			NO	metabo_info.php?molid=39324
39325	760.58	[M+H]+	759.578	9	PC(16:0/18:1(9Z))[S]	C42H82NO8P		C13875	NO	metabo_info.php?molid=39325
39326	760.58	[M+H]+	759.578	9	PC(16:0/18:1(9Z))[U]	C42H82NO8P			NO	metabo_info.php?molid=39326
39420	760.58	[M+H]+	759.578	9	PC(16:1(7Z)/18:0)[U]	C42H82NO8P			NO	metabo_info.php?molid=39420
39427	760.58	[M+H]+	759.578	9	PC(16:1(9Z)/18:0)[U]	C42H82NO8P			NO	metabo_info.php?molid=39427
39615	760.58	[M+H]+	759.578	9	PC(18:1(9E)/16:0)[U]	C42H82NO8P			NO	metabo_info.php?molid=39615
39626	760.58	[M+H]+	759.578	9	PC(18:1(9Z)/16:0)[U]	C42H82NO8P			NO	metabo_info.php?molid=39626
59329	760.58	[M+H]+	759.578	9	PC(14:0/20:1(11Z))	C42H82NO8P		C00157	NO	metabo_info.php?molid=59329
59361	760.58	[M+H]+	759.578	9	PC(14:1(9Z)/20:0)	C42H82NO8P		C00157	NO	metabo_info.php?molid=59361
59420	760.58	[M+H]+	759.578	9	PC(16:0/18:1(11Z))	C42H82NO8P		C00157	NO	metabo_info.php?molid=59420
59421	760.58	[M+H]+	759.578	9	PC(16:0/18:1(9Z))	C42H82NO8P		C00157	NO	metabo_info.php?molid=59421
59451	760.58	[M+H]+	759.578	9	PC(16:1(9Z)/18:0)	C42H82NO8P		C00157	NO	metabo_info.php?molid=59451
59483	760.58	[M+H]+	759.578	9	PC(18:0/16:1(9Z))	C42H82NO8P		C00157	NO	metabo_info.php?molid=59483
59514	760.58	[M+H]+	759.578	9	PC(18:1(11Z)/16:0)	C42H82NO8P		C00157	NO	metabo_info.php?molid=59514
59547	760.58	[M+H]+	759.578	9	PC(18:1(9Z)/16:0)	C42H82NO8P		C00157	NO	metabo_info.php?molid=59547
59709	760.58	[M+H]+	759.578	9	PC(20:0/14:1(9Z))	C42H82NO8P		C00157	NO	metabo_info.php?molid=59709
59741	760.58	[M+H]+	759.578	9	PC(20:1(11Z)/14:0)	C42H82NO8P		C00157	NO	metabo_info.php?molid=59741
60351	760.58	[M+H]+	759.578	9	PE(15:0/22:1(13Z))	C42H82NO8P		C00350	NO	metabo_info.php?molid=60351
60905	760.58	[M+H]+	759.578	9	PE(22:1(13Z)/15:0)	C42H82NO8P		C00350	NO	metabo_info.php?molid=60905
75615	760.58	[M+H]+	759.578	9	PC(12:0/22:1(11Z))	C42H82NO8P			NO	metabo_info.php?molid=75615
75699	760.58	[M+H]+	759.578	9	PC(15:0/19:1(9Z))	C42H82NO8P			NO	metabo_info.php?molid=75699
75728	760.58	[M+H]+	759.578	9	PC(15:1(9Z)/19:0)	C42H82NO8P			NO	metabo_info.php?molid=75728
75783	760.58	[M+H]+	759.578	9	PC(17:0/17:1(9Z))	C42H82NO8P			NO	metabo_info.php?molid=75783

75809	760.58	[M+H]+	759.578	9	PC(17:1(9Z)/17:0)	C42H82NO8P			NO	metabo_info.php?molid=75809
76012	760.58	[M+H]+	759.578	9	PC(19:0/15:1(9Z))	C42H82NO8P			NO	metabo_info.php?molid=76012
76040	760.58	[M+H]+	759.578	9	PC(19:1(9Z)/15:0)	C42H82NO8P			NO	metabo_info.php?molid=76040
76284	760.58	[M+H]+	759.578	9	PC(22:1(11Z)/12:0)	C42H82NO8P			NO	metabo_info.php?molid=76284
76705	760.58	[M+H]+	759.578	9	PE(15:0/22:1(11Z))	C42H82NO8P			NO	metabo_info.php?molid=76705
76735	760.58	[M+H]+	759.578	9	PE(15:1(9Z)/22:0)	C42H82NO8P			NO	metabo_info.php?molid=76735
76769	760.58	[M+H]+	759.578	9	PE(16:1(9Z)/21:0)	C42H82NO8P			NO	metabo_info.php?molid=76769
76787	760.58	[M+H]+	759.578	9	PE(17:0/20:1(11Z))	C42H82NO8P			NO	metabo_info.php?molid=76787
76814	760.58	[M+H]+	759.578	9	PE(17:1(9Z)/20:0)	C42H82NO8P			NO	metabo_info.php?molid=76814
76866	760.58	[M+H]+	759.578	9	PE(18:0/19:1(9Z))	C42H82NO8P			NO	metabo_info.php?molid=76866
76880	760.58	[M+H]+	759.578	9	PE(18:1(9Z)/19:0)	C42H82NO8P			NO	metabo_info.php?molid=76880
77010	760.58	[M+H]+	759.578	9	PE(19:0/18:1(9Z))	C42H82NO8P			NO	metabo_info.php?molid=77010
77039	760.58	[M+H]+	759.578	9	PE(19:1(9Z)/18:0)	C42H82NO8P			NO	metabo_info.php?molid=77039
77064	760.58	[M+H]+	759.578	9	PE(20:0/17:1(9Z))	C42H82NO8P			NO	metabo_info.php?molid=77064
77084	760.58	[M+H]+	759.578	9	PE(20:1(11Z)/17:0)	C42H82NO8P			NO	metabo_info.php?molid=77084
77230	760.58	[M+H]+	759.578	9	PE(21:0/16:1(9Z))	C42H82NO8P			NO	metabo_info.php?molid=77230
77253	760.58	[M+H]+	759.578	9	PE(22:0/15:1(9Z))	C42H82NO8P			NO	metabo_info.php?molid=77253
77279	760.58	[M+H]+	759.578	9	PE(22:1(11Z)/15:0)	C42H82NO8P			NO	metabo_info.php?molid=77279
44825	301.14	[M+Na]+	278.152	2	α-CEHC	C16H22O4	NA		YES	metabo_info.php?molid=44825
53414	301.14	[M+Na]+	278.152	2	Emmotin A	C16H22O4			NO	metabo_info.php?molid=53414
53844	301.14	[M+Na]+	278.152	2	alpha- tocopheronolactone	C16H22O4			NO	metabo_info.php?molid=53844
44802	301.14	[M+Na]+	278.152	2	Phthalic acid Mono-2-ethylhexyl Ester	C16H22O4	4376-20-9	C03343	NO	metabo_info.php?molid=44802
58055	301.14	[M+Na]+	278.152	2	Alpha-CEHC	C16H22O4	4072-32-6		NO	metabo_info.php?molid=58055
69881	301.14	[M+Na]+	278.152	2	Dibutyl phthalate	C16H22O4	84-74-2	C14214	NO	metabo_info.php?molid=69881
70692	301.14	[M+Na]+	278.152	2	Diisobutyl phthalate	C16H22O4	84-69-5	C1[illegible]205	NO	metabo_info.php?molid=70692
65100	517.16	[M+Na]+	494.169	8	TyrMe-TyrMe-OH	C26H26N2O8			YES	metabo_info.php?molid=65100
67647	342.14	[M+H]+	341.126	4	Cassythine	C19H19NO5	4030-51-7	C09380	NO	metabo_info.php?molid=67647
86190	342.14	[M+H]+	341.126	4	Gravacridonediol	C19H19NO5	37551-75-0		NO	metabo_info.php?molid=86190
89210	342.14	[M+H]+	341.126	4	4-Hydroxynornantenine	C19H19NO5	69128-21-8		NO	metabo_info.php?molid=89210

72282	342.14	[M+Na]+	319.145	2	Metconazole	C17H22ClN3O	125116-23-6	C18476	YES	metabo_info.php?molid=72282
44812	342.14	[M+Na]+	319.145	1	4-hydroxy Nonenal Mercapturic Acid	C14H25NO5S	146764-24-1		YES	metabo_info.php?molid=44812
65132	341.13	[M+H]+	340.127	9	Thr-Val-OH	C15H20N2O7			YES	metabo_info.php?molid=65132
65106	341.13	[M+H]+	340.127	9	Leu-Thr-OH	C15H20N2O7			YES	metabo_info.php?molid=65106
65114	341.13	[M+H]+	340.127	9	Ile-Thr-OH	C15H20N2O7			YES	metabo_info.php?molid=65114
65223	341.13	[M+H]+	340.127	9	Ser-Leu-OH	C15H20N2O7			YES	metabo_info.php?molid=65223
65243	341.13	[M+H]+	340.127	9	Ser-Ile-OH	C15H20N2O7			YES	metabo_info.php?molid=65243
72282	320.15	[M+H]+	319.145	3	Metconazole	C17H22ClN3O	125116-23-6	C18476	YES	metabo_info.php?molid=72282
44812	320.15	[M+H]+	319.145	3	4-hydroxy Nonenal Mercapturic Acid	C14H25NO5S	146764-24-1		YES	metabo_info.php?molid=44812
53843	319.15	[M+Na]+	296.162	4	alhpa-tocopheronic acid	C16H24O5			NO	metabo_info.php?molid=53843
67878	319.15	[M+Na]+	296.162	4	Graphinone	C16H24O5	19683-98-8	C09674	NO	metabo_info.php?molid=67878
93953	319.15	[M+Na]+	296.162	4	Methyl dihydrophaseate	C16H24O5			NO	metabo_info.php?molid=93953
95934	319.15	[M+Na]+	296.162	4	3,8-Dihydroxy-6-methoxy-7(11)-eremophilen-12,8-olide	C16H24O5			NO	metabo_info.php?molid=95934
2295	319.15	[M+Na]+	296.162	4	Lactone of PGF-MUM	C16H24O5			NO	metabo_info.php?molid=2295
67637	659.28	[M+Na]+	636.284	5	Calafatimine	C38H40N2O7	77793-42-1	C09369	NO	metabo_info.php?molid=67637
