# Supplementary material for: Phosphatidylcholine could protect the defect of zearalenone exposure on follicular development and oocyte maturation
Source: Aging (Albany NY). 2018 Nov 25;10(11):3486–506. doi: 10.18632/aging.101660 (PMC6286824; doi:10.18632/aging.101660)
Supplement: Supplementary Table 6 [file aging-10-101660-s005.pdf]

Table S6. Top ten differential content metabolites in small and large follicle

Name	Fold change	Log2 |fold change|	P-value	Variation trend	Mass charge ratio (m/z)	retention time (min)	Mean value in small follicle	Standard error in small follicle	Mean value in large follicle	Standard error in large follicle
M522T25_1	3.600669546	1.848265201	9.66E-06	UP	522.350481	25.11589167	32088.50063	9688.98225	115540.087	32180.69213
M524T28[illegible]	5.793439334	2.534420073	2.77E-05	UP	524.366042	28.093375	33910.99475	18526.1128	196461.2909	69676.63654
M520T23[illegible]	5.160193029	2.367425034	3.65E-05	UP	520.3347	22.97724167	18636.15416	6262.90868	96166.15281	33549.90025
M497T24[illegible]	3.51539851	1.813688244	6.12E-05	UP	497.33868	24.11841667	30827.34188	6759.33263	108370.3917	35772.02891
M518T24[illegible]	3.788392626	1.921585858	7.81E-05	UP	518.316795	24.11483333	60435.66775	18031.9794	228954.0381	80768.47132
M496T24[illegible]	3.682526516	1.880695913	0.000112	UP	496.335073	24.11473333	113866.9282	26199.4753	419317.9824	151498.9375
M227T1_1	1.158317089	0.212030245	0.000337	UP	226.949528	1.047791667	377824.0983	29068.6748	437640.1097	31441.56706
M339T40_3	1.487503772	0.572893327	0.001941	UP	339.342307	40.385825	1379431.358	387136.954	2051909.348	438944.8466
M393T44_2	1.635222266	0.709486746	0.004431	UP	393.294307	44.15798333	127814.6378	59286.6843	209005.3416	51858.20172
M340T40[illegible]	1.427038356	0.513024112	0.004466	UP	340.34526	40.38631667	148650.2877	39179.6689	212129.6621	47404.31806
M301T36[illegible]	1.640354698	-0.714007806	0.00016	DOWN	301.208494	35.77916667	204161.8368	27086.5753	124462.0063	42824.96705
M772T0_4	179.5430016	-7.488185609	0.000214	DOWN	771.85274	0.310283333	61579.90948	32644.3019	98.13455975	150.7219977
M257T36[illegible]	1.454362588	-0.540386993	0.000226	DOWN	257.245006	35.77916667	262485.6022	24887.0742	180481.5418	46209.27115
M431T0_1	1.059355783	-0.083187198	0.002717	DOWN	430.910954	0.310258333	1161421.493	49125.9476	1096346.96	29277.52181
M273T48_1	2.89974533	-1.535926201	0.003506	DOWN	273.136469	48.3269	87125.25206	46096.6134	30045.82891	4502.11648
M685T32[illegible]	1.491972075	-0.577220533	0.005749	DOWN	685.430386	51.76326667	196747.4362	57089.6285	131870.723	11585.79844
M327T33[illegible]	1.393825167	-0.47904961	0.006383	DOWN	327.226572	33.40588333	195226.0875	26964.8841	140064.9752	47507.28417
M227T0_1	1.074218539	-0.103287525	0.006528	DOWN	226.949762	0.310266667	2570916.072	119819.316	2393289.613	137318.5994
M227T3[illegible]	4.635915084	-2.212854141	0.006774	DOWN	226.950522	39.15915	49024.91782	33941.0007	10461.4041	17460.6381
M273T47_1	2.360631848	-1.239173064	0.012902	DOWN	273.136098	47.19361667	111826.3169	65058.5419	47371.34976	23233.81525
