# Supplementary material for: Phosphatidylcholine could protect the defect of zearalenone exposure on follicular development and oocyte maturation
Source: Aging (Albany NY). 2018 Nov 25;10(11):3486–506. doi: 10.18632/aging.101660 (PMC6286824; doi:10.18632/aging.101660)
Supplement: Supplementary Table 7 [file aging-10-101660-s006.pdf]

Table S7. Prediction chemicals of the top ten differential content metabolites in small and large follicle

Mol_ID	Input mass	Adduct	Mass	D-ppm	Name	Formula	CAS	KEGG	MS/MS	Link
2081	284.137711	[M+Na]+	261.148	2	5-Hydroxy-6-desmethylprimaquine	C14H19N3O2	87321-06-0		NO	metabo_info.php?molid=2081
40008	522.350481	[M+H]+	521.348	9	PC(O-16:1(11Z)/2:0)	C26H52NO7P			NO	metabo_info.php?molid=40008
40009	522.350481	[M+H]+	521.348	9	PC(O-16:1(9E)/2:0)[U]	C26H52NO7P			NO	metabo_info.php?molid=40009
40010	522.350481	[M+H]+	521.348	9	PC(O-16:1(9Z)/2:0)[U]	C26H52NO7P			NO	metabo_info.php?molid=40010
40174	522.350481	[M+H]+	521.348	9	PC(P-16:0/2:0)	C26H52NO7P			NO	metabo_info.php?molid=40174
40295	522.350481	[M+H]+	521.348	9	PC(18:1(6Z)/0:0)	C26H52NO7P			NO	metabo_info.php?molid=40295
40296	522.350481	[M+H]+	521.348	9	PC(18:1(9E)/0:0)	C26H52NO7P			NO	metabo_info.php?molid=40296
40297	522.350481	[M+H]+	521.348	9	PC(18:1(9)/0:0)[U]	C26H52NO7P			NO	metabo_info.php?molid=40297
40298	522.350481	[M+H]+	521.348	9	PC(18:1(9Z)/0:0)	C26H52NO7P			NO	metabo_info.php?molid=40298
40299	522.350481	[M+H]+	521.348	9	PC(18:1(9Z)/0:0)[U]	C26H52NO7P		C03916	NO	metabo_info.php?molid=40299
40345	522.350481	[M+H]+	521.348	9	PC(0:0/18:1(6Z))	C26H52NO7P			NO	metabo_info.php?molid=40345
40346	522.350481	[M+H]+	521.348	9	PC(0:0/18:1(9E))	C26H52NO7P			NO	metabo_info.php?molid=40346
40347	522.350481	[M+H]+	521.348	9	PC(0:0/18:1(9E))[U]	C26H52NO7P			NO	metabo_info.php?molid=40347
40348	522.350481	[M+H]+	521.348	9	PC(0:0/18:1(9Z))	C26H52NO7P			NO	metabo_info.php?molid=40348
40349	522.350481	[M+H]+	521.348	9	PC(0:0/18:1(9Z))[U]	C26H52NO7P			NO	metabo_info.php?molid=40349
46689	522.350481	[M+H]+	521.348	9	PC(18:1(9E)/0:0)[U]	C26H52NO7P			NO	metabo_info.php?molid=46689
61695	522.350481	[M+H]+	521.348	9	LysoPC(18:1(11Z))	C26H52NO7P		C04230	NO	metabo_info.php?molid=61695
1E+05	522.350481	[M+H]+	521.348	9	PC(18:1(9Z)/0:0)[rac]	C26H52NO7P			NO	metabo_info.php?molid=10276[illegible]
184	522.350481	[M+H]+	521.348	9	PC(18:1(9Z)/0:0)[U] / PC(18:1(9Z)/0:0)[rac]	C26H52NO7P	3542-29-8		NO	metabo_info.php?molid=184
43413	524.366042	[M+H]+	523.364	9	enantio-PAF C-16	C26H54NO7P	117985-57-6		YES	metabo_info.php?molid=43413
34488	524.366042	[M+H]+	523.364	9	PAF C-16	C26H54NO7P	74389-68-7		YES	metabo_info.php?molid=34488
40161	524.366042	[M+H]+	523.364	9	PC(2:0/O-16:0)[U]	C26H54NO7P			NO	metabo_info.php?molid=40161
40075	524.366042	[M+H]+	523.364	9	PC(O-16:0/2:0)	C26H54NO7P			NO	metabo_info.php?molid=40075
40076	524.366042	[M+H]+	523.364	9	PC(O-16:0/2:0)[S]	C26H54NO7P			NO	metabo_info.php?molid=40076

40077	524.366042	[M+H]+	523.364	9	PC(O-16:0/2:0)[U]	C26H54NO7P			NO	metabo_info.php?molid=40077
40292	524.366042	[M+H]+	523.364	9	PC(18:0/0:0)	C26H54NO7P			NO	metabo_info.php?molid=40292
40293	524.366042	[M+H]+	523.364	9	PC(18:0/0:0)[S]	C26H54NO7P			NO	metabo_info.php?molid=40293
40294	524.366042	[M+H]+	523.364	9	PC(18:0/0:0)[U]	C26H54NO7P			NO	metabo_info.php?molid=40294
40342	524.366042	[M+H]+	523.364	9	PC(0:0/18:0)	C26H54NO7P			NO	metabo_info.php?molid=40342
40343	524.366042	[M+H]+	523.364	9	PC(0:0/18:0)[S]	C26H54NO7P			NO	metabo_info.php?molid=40343
40344	524.366042	[M+H]+	523.364	9	PC(0:0/18:0)[U]	C26H54NO7P			NO	metabo_info.php?molid=40344
61694	524.366042	[M+H]+	523.364	9	LysoPC(18:0)	C26H54NO7P		C042 30	NO	metabo_info.php?molid=61694
61991	524.366042	[M+H]+	523.364	9	LysoPC(0:0/18:0)	C26H54NO7P			NO	metabo_info.php?molid=61991
77692	524.366042	[M+H]+	523.364	9	PE(21:0/0:0)	C26H54NO7P			NO	metabo_info.php?molid=77692
1	520.3347	[M+H]+	519.332	9	1-Linoleoylglycerophosphocholine	C26H50NO7P	15895-41-7	C041 00	NO	metabo_info.php?molid=1
40300	520.3347	[M+H]+	519.332	9	PC(18:2(2E,4E)/0:0)	C26H50NO7P			NO	metabo_info.php?molid=40300
40301	520.3347	[M+H]+	519.332	9	PC(18:2(9Z,12Z)/0:0)	C26H50NO7P			NO	metabo_info.php?molid=40301
40302	520.3347	[M+H]+	519.332	9	PC(18:2(9Z,12Z)/0:0)[U]	C26H50NO7P			NO	metabo_info.php?molid=40302
61696	520.3347	[M+H]+	519.332	9	LysoPC(18:2(9Z,12Z))	C26H50NO7P		C042 30	NO	metabo_info.php?molid=61696
86851	497.33868	[M+Na]+	474.346	7	Dehydrocarpaine II	C28H46N2O4	72362-03-9		NO	metabo_info.php?molid=86851
182	518.316795	[M+Na]+	495.332	9	PC(16:0/0:0)[U] / PC(16:0/0:0)[rac]	C24H50NO7P	17364-16-8	C041 02	YES	metabo_info.php?molid=182
40048	518.316795	[M+Na]+	495.332	9	PC(O-14:0/2:0)	C24H50NO7P			NO	metabo_info.php?molid=40048
40049	518.316795	[M+Na]+	495.332	9	PC(O-14:0/2:0)[U]	C24H50NO7P			NO	metabo_info.php?molid=40049
40284	518.316795	[M+Na]+	495.332	9	PC(16:0/0:0)	C24H50NO7P			NO	metabo_info.php?molid=40284
40285	518.316795	[M+Na]+	495.332	9	PC(16:0/0:0)[S]	C24H50NO7P			NO	metabo_info.php?molid=40285
40286	518.316795	[M+Na]+	495.332	9	PC(16:0/0:0)[U]	C24H50NO7P			NO	metabo_info.php?molid=40286
40340	518.316795	[M+Na]+	495.332	9	PC(0:0/16:0)	C24H50NO7P			NO	metabo_info.php?molid=40340
40341	518.316795	[M+Na]+	495.332	9	PC(0:0/16:0)[U]	C24H50NO7P			NO	metabo_info.php?molid=40341
61692	518.316795	[M+Na]+	495.332	9	LysoPC(16:0)	C24H50NO7P		C042 30	NO	metabo_info.php?molid=61692
77694	518.316795	[M+Na]+	495.332	9	PE(19:0/0:0)	C24H50NO7P			NO	metabo_info.php?molid=77694
1E+05	518.316795	[M+Na]+	495.332	9	PC(16:0/0:0)[rac]	C24H50NO7P			NO	metabo_info.php?molid=102768

182	496.335073	[M+H]+	495.332	9	PC(16:0/0:0)[U] / PC(16:0/0:0)[rac]	C24H50NO7P	17364-16-8	C04102	YES	metabo_info.php?molid=182
40048	496.335073	[M+H]+	495.332	9	PC(O-14:0/2:0)	C24H50NO7P			NO	metabo_info.php?molid=40048
40049	496.335073	[M+H]+	495.332	9	PC(O-14:0/2:0)[U]	C24H50NO7P			NO	metabo_info.php?molid=40049
40284	496.335073	[M+H]+	495.332	9	PC(16:0/0:0)	C24H50NO7P			NO	metabo_info.php?molid=40284
40285	496.335073	[M+H]+	495.332	9	PC(16:0/0:0)[S]	C24H50NO7P			NO	metabo_info.php?molid=40285
40286	496.335073	[M+H]+	495.332	9	PC(16:0/0:0)[U]	C24H50NO7P			NO	metabo_info.php?molid=40286
40340	496.335073	[M+H]+	495.332	9	PA	C24H50NO7P			NO	metabo_info.php?molid=40340
40341	496.335073	[M+H]+	495.332	9	PC(0:0/16:0)[U]	C24H50NO7P			NO	metabo_info.php?molid=40341
61692	496.335073	[M+H]+	495.332	9	LysoPC(16:0)	C24H50NO7P		C04230	NO	metabo_info.php?molid=61692
77694	496.335073	[M+H]+	495.332	9	PE(19:0/0:0)	C24H50NO7P			NO	metabo_info.php?molid=77694
1E+05	496.335073	[M+H]+	495.332	9	PC(16:0/0:0)[rac]	C24H50NO7P			NO	metabo_info.php?molid=1027[illegible]8
58392	496.335073	[M+Na]+	473.351	9	Docosa-4,7,10,13,16-pentaenoyl carnitine	C29H47NO4			NO	metabo_info.php?molid=58392
58428	496.335073	[M+Na]+	473.351	9	Clupanodonyl carnitine	C29H47NO4			NO	metabo_info.php?molid=58428
87655	496.335073	[M+Na]+	473.351	9	23-Acetoxysoladulcidine	C29H47NO4	152128-85-3		NO	metabo_info.php?molid=87655
24084	257.245006	[M+H]+	256.24	9	2-Hexyldecanoic acid	C16H32O2	25354-97-6		YES	metabo_info.php?molid=24084
34669	257.245006	[M+H]+	256.24	9	4,8,12-trimethyl-tridecanoic acid	C16H32O2			NO	metabo_info.php?molid=34669
4289	257.245006	[M+H]+	256.24	9	Isopalmitic acid	C16H32O2	######		YES	metabo_info.php?molid=4289
4320	257.245006	[M+H]+	256.24	9	2,6-dimethyl-tetradecanoic acid	C16H32O2			NO	metabo_info.php?molid=4320
4321	257.245006	[M+H]+	256.24	9	2,8-dimethyl-tetradecanoic acid	C16H32O2			NO	metabo_info.php?molid=4321
45004	257.245006	[M+H]+	256.24	9	Myristic Acid ethyl ester	C16H32O2	124-06-1		YES	metabo_info.php?molid=45004
187	257.245006	[M+H]+	256.24	9	Palmitic acid	C16H32O2	######	C00249	YES	metabo_info.php?molid=187
45768	257.245006	[M+H]+	256.24	9	13,13-dimethyl-tetradecanoic acid	C16H32O2			NO	metabo_info.php?molid=45768
46330	257.245006	[M+H]+	256.24	9	Tetradecyl acetate	C16H32O2			NO	metabo_info.php?molid=46330
73615	257.245006	[M+H]+	256.24	9	3-methyl-pentadecanoic acid	C16H32O2			NO	metabo_info.php?molid=73615
73616	257.245006	[M+H]+	256.24	9	2-propyl-tridecanoic acid	C16H32O2			NO	metabo_info.php?molid=73616

73617	257.245006	[M+H]+	256.24	9	2-hexyl-decanoic acid	C16H32O2			NO	metabo_info.php?molid=73617
73618	257.245006	[M+H]+	256.24	9	3-ethyl-3-methyl-tridecanoic acid	C16H32O2			NO	metabo_info.php?molid=73618
73619	257.245006	[M+H]+	256.24	9	2-heptyl-nonanoic acid	C16H32O2			NO	metabo_info.php?molid=73619
73620	257.245006	[M+H]+	256.24	9	6-ethyl-tetradecanoic acid	C16H32O2			NO	metabo_info.php?molid=73620
73621	257.245006	[M+H]+	256.24	9	2,4-dimethyl-tetradecanoic acid	C16H32O2			NO	metabo_info.php?molid=73621
73622	257.245006	[M+H]+	256.24	9	[illegible]-dimethyl-tetradecanoic acid	C16H32O2			NO	metabo_info.php?molid=73622
73623	257.245006	[M+H]+	256.24	9	4-hexyl-decanoic acid	C16H32O2			NO	metabo_info.php?molid=73623
73624	257.245006	[M+H]+	256.24	9	2-ethyl-2-butyl-decanoic acid	C16H32O2			NO	metabo_info.php?molid=73624
73638	257.245006	[M+H]+	256.24	9	1[illegible]-methyl-pentadecanoic acid	C16H32O2			NO	metabo_info.php?molid=73638
73688	257.245006	[M+H]+	256.24	9	Tridecanoic acid, 4,8,12-trimethyl-; 4,8,12-Trimethyltridecanoic acid	C16H32O2			NO	metabo_info.php?molid=73688
73765	257.245006	[M+H]+	256.24	9	14:0(10Me,13Me)	C16H32O2			NO	metabo_info.php?molid=73765
75391	257.245006	[M+H]+	256.24	9	2-hydroxyhexadecanal	C16H32O2			NO	metabo_info.php?molid=75391
88155	257.245006	[M+H]+	256.24	9	Butyl dodecanoate	C16H32O2	106-18-3		NO	metabo_info.php?molid=88155
88297	257.245006	[M+H]+	256.24	9	Dodecyl butyrate	C16H32O2	3724-61-6		NO	metabo_info.php?molid=88297
88363	257.245006	[M+H]+	256.24	9	Hexyl decanoate	C16H32O2	10448-26-7		NO	metabo_info.php?molid=88363
89043	257.245006	[M+H]+	256.24	9	Octyl octanoate	C16H32O2	2306-88-9		NO	metabo_info.php?molid=89043
91358	257.245006	[M+H]+	256.24	9	Dodecyl 2-methylpropanoate	C16H32O2	6624-71-1		NO	metabo_info.php?molid=91358
97177	257.245006	[M+H]+	256.24	9	decyl hexanoate	C16H32O2			NO	metabo_info.php?molid=97177
97207	257.245006	[M+H]+	256.24	9	formyl 2,6,10-trimethyl-dodecanoate	C16H32O2			NO	metabo_info.php?molid=97207
97294	257.245006	[M+H]+	256.24	9	7-Methyloctyl 5-methylhexanoate	C16H32O2			NO	metabo_info.php?molid=97294
69492	226.949528	[M+H]+	225.944	5	2,5-Dichloro-4-oxohex-2-enedioate	C6H4Cl2O5		C12835	NO	metabo_info.php?molid=69492
66759	273.136469	[M+H]+	272.129	0	Procaine hydrochloride	C13H21ClN2O2	######	C07894	NO	metabo_info.php?molid=66759

67546	273.136469	[M+H]+	272.131	7	Sempervirine	C19H16N2	6882-99-1	C09240	NO	metabo_info.php?molid=67546
1271	273.136469	[M+Na]+	250.147	0	Desmethylmianserin	C17H18N2	71936-92-0		NO	metabo_info.php?molid=1271
35989	393.294307	[M+Na]+	370.308	8	Docosanedioic acid	C22H42O4		C19625	NO	metabo_info.php?molid=35989
69902	393.294307	[M+Na]+	370.308	8	Di(2-ethylhexyl) adipate	C22H42O4	103-23-1	C14240	NO	metabo_info.php?molid=69902
95990	393.294307	[M+Na]+	370.308	8	Dioctyl hexanedioate	C22H42O4	123-79-5		NO	metabo_info.php?molid=95990
93069	685.430386	[M+Na]+	662.439	2	Goyaglycoside c	C38H62O9	333332-49-3		NO	metabo_info.php?molid=93069
64806	685.430386	[M+Na]+	662.441	0	3-hexanoyl-NBD Cholesterol	C39H58N4O5	NA		YES	metabo_info.php?molid=64806
72401	327.226572	[M+Na]+	304.238	1	Sodium oleate	C18H33NaO2	143-19-1	C18601	NO	metabo_info.php?molid=72401
24087	327.226572	[M+Na]+	304.24	8	8,11-eicosadiynoic acid	C20H32O2	82073-91-4		YES	metabo_info.php?molid=24087
44955	327.226572	[M+Na]+	304.24	8	Stearidonic Acid ethyl ester	C20H32O2	119798-44-6		YES	metabo_info.php?molid=44955
34842	327.226572	[M+Na]+	304.24	8	4,8,12,16-eicosatetraenoic acid	C20H32O2			NO	metabo_info.php?molid=34842
34843	327.226572	[M+Na]+	304.24	8	6,10,14,18-eicosatetraenoic acid	C20H32O2			NO	metabo_info.php?molid=34843
34844	327.226572	[M+Na]+	304.24	8	8,11,14,17-eicosatetraenoic acid	C20H32O2			NO	metabo_info.php?molid=34844
35052	327.226572	[M+Na]+	304.24	8	4,7,10,13-Eicosatetraenoic acid	C20H32O2			NO	metabo_info.php?molid=35052
35053	327.226572	[M+Na]+	304.24	8	4Z,7Z,10Z,13Z-eicosatetraenoic acid	C20H32O2			NO	metabo_info.php?molid=35053
35054	327.226572	[M+Na]+	304.24	8	4Z,8Z,11Z,14Z-eicosatetraenoic acid	C20H32O2			NO	metabo_info.php?molid=35054
35055	327.226572	[M+Na]+	304.24	8	5,11,14,17-Eicosatetraenoic acid	C20H32O2			NO	metabo_info.php?molid=35055
35056	327.226572	[M+Na]+	304.24	8	5,8,11,14-Eicosatetraenoic acid / 5,8,11,14-icosatetraenoic acid	C20H32O2			NO	metabo_info.php?molid=35056

35057	327.226572	[M+Na]+	304.24	8	5Z,11Z,14Z,17Z-eicosatetraenoic acid	C20H32O2			NO	metabo_info.php?molid=35057
35058	327.226572	[M+Na]+	304.24	8	8Z,11Z,14Z,18Z-eicosatetraenoic acid	C20H32O2			NO	metabo_info.php?molid=35058
41846	327.226572	[M+Na]+	304.24	8	Mestanolone	C20H32O2			NO	metabo_info.php?molid=41846
193	327.226572	[M+Na]+	304.24	8	Arachidonic Acid (peroxide free)	C20H32O2	506-32-1	C00219	YES	metabo_info.php?molid=193
41259	327.226572	[M+Na]+	304.24	8	Taxa-4(20),11(12)-dien-5α,13α-diol	C20H32O2		C11897	NO	metabo_info.php?molid=41259
41839	327.226572	[M+Na]+	304.24	8	Methandriol	C20H32O2		C14493	NO	metabo_info.php?molid=41839
53642	327.226572	[M+Na]+	304.24	8	(+)-Serradiol	C20H32O2			NO	metabo_info.php?molid=53642
53661	327.226572	[M+Na]+	304.24	8	(-)-Cladielline	C20H32O2			NO	metabo_info.php?molid=53661
53671	327.226572	[M+Na]+	304.24	8	(-)-Amijiol	C20H32O2			NO	metabo_info.php?molid=53671
53672	327.226572	[M+Na]+	304.24	8	(-)-Isoamijiol	C20H32O2			NO	metabo_info.php?molid=53672
53699	327.226572	[M+Na]+	304.24	8	Isotrinervidiol	C20H32O2			NO	metabo_info.php?molid=53699
53713	327.226572	[M+Na]+	304.24	8	Acutilol A	C20H32O2		C09057	NO	metabo_info.php?molid=53713
53723	327.226572	[M+Na]+	304.24	8	(-)-Reiswigin A	C20H32O2			NO	metabo_info.php?molid=53723
57837	327.226572	[M+Na]+	304.24	8	Mesterolone	C20H32O2	NA		NO	metabo_info.php?molid=57837
62952	327.226572	[M+Na]+	304.24	8	omega-3 Arachidonic Acid	C20H32O2	24880-40-8		YES	metabo_info.php?molid=62952
67447	327.226572	[M+Na]+	304.24	8	2-Ketoepimanool	C20H32O2	86561-13-9	C09124	NO	metabo_info.php?molid=67447
70197	327.226572	[M+Na]+	304.24	8	Metholone	C20H32O2		C14605	NO	metabo_info.php?molid=70197
70455	327.226572	[M+Na]+	304.24	8	3alpha-Hydroxy-2alpha-methyl-5alpha-androstan-17-one	C20H32O2		C14956	NO	metabo_info.php?molid=70455
70666	327.226572	[M+Na]+	304.24	8	17-Methyl-5alpha-androst-2-ene-1alpha,17beta-diol	C20H32O2		C15176	NO	metabo_info.php?molid=70666
70729	327.226572	[M+Na]+	304.24	8	17beta-Hydroxy-4alpha-methyl-5alpha-androstan-3-one	C20H32O2		C15252	NO	metabo_info.php?molid=70729
70839	327.226572	[M+Na]+	304.24	8	3beta-Methoxyandrost-5-en-16beta-ol	C20H32O2		C15367	NO	metabo_info.php?molid=70839

71945	327.226572	[M+Na]+	304.24	8	Dihydroabietic acid	C20H32O2	19407-37-5	C17920	NO	metabo_info.php?molid=71945
73668	327.226572	[M+Na]+	304.24	8	18-methyl-5Z,8Z,11Z,14Z-nonadecatetraenoic acid	C20H32O2			NO	metabo_info.php?molid=73668
73777	327.226572	[M+Na]+	304.24	8	16:3(2E,10E,12E)(3Me,5Me[R],7My,15Me)	C20H32O2			NO	metabo_info.php?molid=73777
73790	327.226572	[M+Na]+	304.24	8	17:4(2E,4E,9E,11E)(8Me[R],10Me,15Me[R])	C20H32O2			NO	metabo_info.php?molid=73790
73864	327.226572	[M+Na]+	304.24	8	4, 8, 12, 16-icosatetraenoic acid; C20:4n-4,8,12,16	C20H32O2			NO	metabo_info.php?molid=73864
73865	327.226572	[M+Na]+	304.24	8	6, 10, 14, 18-icosatetraenoic acid; C20:4n-2,6,10,14	C20H32O2			NO	metabo_info.php?molid=73865
73866	327.226572	[M+Na]+	304.24	8	8, 11, 14, 17-icosatetraenoic acid; C20:4n-3,6,9,12	C20H32O2			NO	metabo_info.php?molid=73866
74056	327.226572	[M+Na]+	304.24	8	4,7,10,13-Eicosatetraenoic acid	C20H32O2			NO	metabo_info.php?molid=74056
74057	327.226572	[M+Na]+	304.24	8	4Z,7Z,10Z,13Z-eicosatetraenoic acid	C20H32O2			NO	metabo_info.php?molid=74057
74058	327.226572	[M+Na]+	304.24	8	C20:4n-6,9,12,16	C20H32O2			NO	metabo_info.php?molid=74058
74059	327.226572	[M+Na]+	304.24	8	C20:4n-3,6,9,15	C20H32O2			NO	metabo_info.php?molid=74059
74060	327.226572	[M+Na]+	304.24	8	C20:4n-6,9,12,15	C20H32O2			NO	metabo_info.php?molid=74060
74061	327.226572	[M+Na]+	304.24	8	Juniperonic acid	C20H32O2			NO	metabo_info.php?molid=74061
74062	327.226572	[M+Na]+	304.24	8	C20:4n-2,6,9,12	C20H32O2			NO	metabo_info.php?molid=74062
74288	327.226572	[M+Na]+	304.24	8	7,13-Eicosadiynoic acid	C20H32O2			NO	metabo_info.php?molid=74288
74290	327.226572	[M+Na]+	304.24	8	10,13-Eicosadiynoic acid	C20H32O2			NO	metabo_info.php?molid=74290
74352	327.226572	[M+Na]+	304.24	8	C20:4n-6,9,12,18	C20H32O2			NO	metabo_info.php?molid=74352
74353	327.226572	[M+Na]+	304.24	8	5(E)-Arachidonic acid	C20H32O2			NO	metabo_info.php?molid=74353
74354	327.226572	[M+Na]+	304.24	8	omega-3-Arachidonic acid	C20H32O2			NO	metabo_info.php?molid=74354
74407	327.226572	[M+Na]+	304.24	8	20:4(5Z,13Z,16Z,19Z)	C20H32O2			NO	metabo_info.php?molid=74407
74440	327.226572	[M+Na]+	304.24	8	17:4(2E,4E,9E,11E)(7Me[R],10Me,13Me[S])	C20H32O2			NO	metabo_info.php?molid=74440
74441	327.226572	[M+Na]+	304.24	8	16:3(2E,9E,11E)(3Me,5Me[S],7My,15Me)	C20H32O2			NO	metabo_info.php?molid=74441
74893	327.226572	[M+Na]+	304.24	8	8-[3]-ladderane-octanoic acid	C20H32O2			NO	metabo_info.php?molid=74893

91734	327.226572	[M+Na]+	304.24	8	Sideridiol	C20H32O2	19891-25-9		NO	metabo_info.php?molid=91734
91751	327.226572	[M+Na]+	304.24	8	ent-17-Hydroxy-16beta-kauran-19-al	C20H32O2	41756-43-8		NO	metabo_info.php?molid=91751
91781	327.226572	[M+Na]+	304.24	8	Yucalexin P21	C20H32O2	119626-54-9		NO	metabo_info.php?molid=91781
91842	327.226572	[M+Na]+	304.24	8	Copalic acid	C20H32O2	20257-75-4		NO	metabo_info.php?molid=91842
91843	327.226572	[M+Na]+	304.24	8	Junicedral	C20H32O2	70901-87-0		NO	metabo_info.php?molid=91843
92634	327.226572	[M+Na]+	304.24	8	7,13-Eperudien-15-oic acid	C20H32O2	14022-43-6		NO	metabo_info.php?molid=92634
94242	327.226572	[M+Na]+	304.24	8	Oryzalexin S	C20H32O2	143437-61-0		NO	metabo_info.php?molid=94242
94257	327.226572	[M+Na]+	304.24	8	Oryzalexin E	C20H32O2	150943-96-7		NO	metabo_info.php?molid=94257
69492	226.949762	[M+H]+	225.944	4	2,5-Dichloro-4-oxohex-2-enedioate	C6H4Cl2O5		C12835	NO	metabo_info.php?molid=69492
69492	226.950522	[M+H]+	225.944	1	2,5-Dichloro-4-oxohex-2-enedioate	C6H4Cl2O5		C12835	NO	metabo_info.php?molid=69492
45078	338.33917	[M+H]+	337.334	7	N-Cyclohexanecarbonylpentadecylamine	C22H43NO	702638-84-4		YES	metabo_info.php?molid=45078
64926	338.33917	[M+H]+	337.334	7	13Z-Docosenamide	C22H43NO	112-84-5		YES	metabo_info.php?molid=64926
85041	338.33917	[M+H]+	337.334	7	13E-Docosenamide	C22H43NO			YES	metabo_info.php?molid=85041
66759	273.136098	[M+H]+	272.129	1	Procaine hydrochloride	C13H21ClN2O2	######	C07894	NO	metabo_info.php?molid=66759
67546	273.136098	[M+H]+	272.131	9	Sempervirine	C19H16N2	6882-99-1	C09240	NO	metabo_info.php?molid=67546
1271	273.136098	[M+Na]+	250.147	0	Desmethylmianserin	C17H18N2	71936-92-0		NO	metabo_info.php?molid=1271
1E+05	764.604923	[M+Na]+	741.612	5	GlcCer(d15:1/22:0)	C43H83NO8			NO	metabo_info.php?molid=10328[illegible]

45078	338.339116	[M+H]+	337.334	7	N-Cyclohexanecarbonylpentadecylamine	C22H43NO	702638-84-4		YES	metabo_info.php?molid=45078
64926	338.339116	[M+H]+	337.334	7	13Z-Docosenamide	C22H43NO	112-84-5		YES	metabo_info.php?molid=64926
85041	338.339116	[M+H]+	337.334	7	13E-Docosenamide	C22H43NO			YES	metabo_info.php?molid=85041
66759	273.135559	[M+H]+	272.129	3	Procaine hydrochloride	C13H21ClN2O2	######	C07894	NO	metabo_info.php?molid=66759
1271	273.135559	[M+Na]+	250.147	2	Desmethylmianserin	C17H18N2	71936-92-0		NO	metabo_info.php?molid=1271
66759	273.13603	[M+H]+	272.129	1	Procaine hydrochloride	C13H21ClN2O2	######	C07894	NO	metabo_info.php?molid=66759
67546	273.13603	[M+H]+	272.131	9	Sempervirine	C19H16N2	6882-99-1	C09240	NO	metabo_info.php?molid=67546
1271	273.13603	[M+Na]+	250.147	0	Desmethylmianserin	C17H18N2	71936-92-0		NO	metabo_info.php?molid=1271
133	203.050742	[M+Na]+	180.063	9	α-D-Glucose	C6H12O6	492-62-6	C00267	YES	metabo_info.php?molid=133
134	203.050742	[M+Na]+	180.063	9	D-Galactose	C6H12O6	59-23-4	C00124	YES	metabo_info.php?molid=134
135	203.050742	[M+Na]+	180.063	9	D-Fructose	C6H12O6	57-48-7	C00095	YES	metabo_info.php?molid=135
136	203.050742	[M+Na]+	180.063	9	D-(+)-Mannose	C6H12O6	3458-28-4	C00159	YES	metabo_info.php?molid=136
144	203.050742	[M+Na]+	180.063	9	myo-Inositol	C6H12O6	######	C00137	YES	metabo_info.php?molid=144
348	203.050742	[M+Na]+	180.063	9	Allose	C6H12O6	6038-51-3	C01582	YES	metabo_info.php?molid=348
3754	203.050742	[M+Na]+	180.063	9	b-D-Galactopyranose	C6H12O6	7296-64-2	C17207	YES	metabo_info.php?molid=3754
3755	203.050742	[M+Na]+	180.063	9	b-Glucose	C6H12O6	492-61-5	C00221	NO	metabo_info.php?molid=3755
4184	203.050742	[M+Na]+	180.063	9	L-Galactose	C6H12O6	15572-79-9	C01825	NO	metabo_info.php?molid=4184

44877	203.050742	[M+Na]+	180.063	9	allo-Inositol	C6H12O6	643-10-7	C00137	NO	metabo_info.php?molid=44877
58151	203.050742	[M+Na]+	180.063	9	D-Tagatose	C6H12O6	87-81-0	C00795	YES	metabo_info.php?molid=58151
63118	203.050742	[M+Na]+	180.063	9	α-D-Glucose	C6H12O6	492-62-6	C00267	NO	metabo_info.php?molid=63118
63163	203.050742	[M+Na]+	180.063	9	L-Rhamnonate	C6H12O6	6422-34-0	C01934	NO	metabo_info.php?molid=63163
63168	203.050742	[M+Na]+	180.063	9	D-Fuconate	C6H12O6		C01680	NO	metabo_info.php?molid=63168
63197	203.050742	[M+Na]+	180.063	9	β-D-Fructose	C6H12O6	53188-23-1	C02336	NO	metabo_info.php?molid=63197
64618	203.050742	[M+Na]+	180.063	9	L-(+)-Gulose	C6H12O6	6027-89-0	C15923	YES	metabo_info.php?molid=64618
65598	203.050742	[M+Na]+	180.063	9	Sorbose	C6H12O6	3615-39-2	C01452	NO	metabo_info.php?molid=65598
65675	203.050742	[M+Na]+	180.063	9	D-Hamamelose	C6H12O6	4573-78-8	C01906	NO	metabo_info.php?molid=65675
65829	203.050742	[M+Na]+	180.063	9	2-Deoxy-D-gluconate	C6H12O6		C02782	NO	metabo_info.php?molid=65829
66926	203.050742	[M+Na]+	180.063	9	beta-D-Hamamelopyranose	C6H12O6		C08351	NO	metabo_info.php?molid=66926
68675	203.050742	[M+Na]+	180.063	9	D-Fructose	C6H12O6	57-48-7	C10906	YES	metabo_info.php?molid=68675
4131	203.050742	[M+Na]+	180.063	9	1,3-Dihydroxyacetone	C6H12O6	62147-49-3		NO	metabo_info.php?molid=4131
5335	203.050742	[M+Na]+	180.063	9	3-Deoxyarabinohexonic acid	C6H12O6			NO	metabo_info.php?molid=5335
66759	273.136079	[M+H]+	272.129	1	Procaine hydrochloride	C13H21ClN2O2	######	C07894	NO	metabo_info.php?molid=66759
67546	273.136079	[M+H]+	272.131	9	Sempervirine	C19H16N2	6882-99-1	C09240	NO	metabo_info.php?molid=67546
1271	273.136079	[M+Na]+	250.147	0	Desmethylmianserin	C17H18N2	71936-92-0		NO	metabo_info.php?molid=1271

1802	426.13567	[M+Na]+	403.149	4	Perphenazine	C21H26ClN3OS	58-39-9	C07427	YES	metabo_info.php?molid=1802
36396	425.131742	[M+H]+	424.125	0	bromovulone I	C21H29BrO4			NO	metabo_info.php?molid=36396
65111	425.131742	[M+H]+	424.127	6	Nap-Thr-OH	C22H20N2O7			YES	metabo_info.php?molid=65111
65323	425.131742	[M+H]+	424.127	6	Ser-Nap-OH	C22H20N2O7			YES	metabo_info.php?molid=65323
67357	425.131742	[M+H]+	424.127	6	Adifoline	C22H20N2O7	20072-28-0	C09020	NO	metabo_info.php?molid=67357
65082	425.131742	[M+Na]+	402.143	0	Val-TyrMe-OH	C20H22N2O7			YES	metabo_info.php?molid=65082
65124	425.131742	[M+Na]+	402.143	0	Tyr-Val-OH	C20H22N2O7			YES	metabo_info.php?molid=65124
65171	425.131742	[M+Na]+	402.143	0	Leu-Tyr-OH	C20H22N2O7			YES	metabo_info.php?molid=65171
65179	425.131742	[M+Na]+	402.143	0	Ile-Tyr-OH	C20H22N2O7			YES	metabo_info.php?molid=65179
65300	425.131742	[M+Na]+	402.143	0	TyrMe-Abu-OH	C20H22N2O7			YES	metabo_info.php?molid=65300
65348	425.131742	[M+Na]+	402.143	0	Thr-HoPhe-OH	C20H22N2O7			YES	metabo_info.php?molid=65348
1544	425.131742	[M+Na]+	402.143	0	desmethyldehydronimodipine	C20H22N2O7	82219-48-5		NO	metabo_info.php?molid=1544
2837	425.131742	[M+Na]+	402.144	2	Thioridazine 5-sulfone	C21H26N2O2S2	7651-42-5		NO	metabo_info.php?molid=2837
2838	425.131742	[M+Na]+	402.144	2	Thioridazine-2-sulfone	C21H26N2O2S2	14759-06-9		NO	metabo_info.php?molid=2838
2841	425.131742	[M+Na]+	402.144	2	Thioridazine 2,5-disulfoxide	C21H26N2O2S2	53926-89-9		NO	metabo_info.php?molid=2841
47723	425.131742	[M+Na]+	402.147	9	Ulexone B	C25H22O5			NO	metabo_info.php?molid=47723
48384	425.131742	[M+Na]+	402.147	9	cis-and-trans-Inophyllolide	C25H22O5			NO	metabo_info.php?molid=48384
48387	425.131742	[M+Na]+	402.147	9	Tomentolide A	C25H22O5			NO	metabo_info.php?molid=48387
48869	777.227879	[M+Na]+	754.232	8	2\'\'\'-O-Rhamnosyl-2\'\'-O-glucosylcytisoside	C34H42O19			NO	metabo_info.php?molid=48869
86898	777.227879	[M+Na]+	754.232	8	1,2-Disinapoylgentiobiose	C34H42O19			NO	metabo_info.php?molid=86898
48891	777.227879	[M+Na]+	754.232	8	Acacetin 7-O-[2\'\'\'-O-rhamnosyl-2\'\'-O-glucosylglucoside	C34H42O19			NO	metabo_info.php?molid=48891
48989	777.227879	[M+Na]+	754.232	8	Kaempferol 4\'-methyl ether 3-(4Rha-rhamnosylrutinoside)	C34H42O19			NO	metabo_info.php?molid=48989
49247	777.227879	[M+Na]+	754.232	8	Luteolin 4\'-methyl ether 7-(4G-rhamnosylneohesperidoside	C34H42O19			NO	metabo_info.php?molid=49247

49250	777.227879	[M+Na]+	754.232	8	Diosmetin 7-(2\'\',6\'\'-dirhamnosyl)-glucoside	C34H42O19			NO	metabo_info.php?molid=49250
49437	777.227879	[M+Na]+	754.232	8	Swertisin 4\'-O-glucoside-2\'\'-O-rhamnoside	C34H42O19			NO	metabo_info.php?molid=49437
50992	777.227879	[M+Na]+	754.232	8	Rhamnocitrin 3-rhamninoside	C34H42O19			NO	metabo_info.php?molid=50992
50993	777.227879	[M+Na]+	754.232	8	Rhamnocitrin 3-isorhamninoside	C34H42O19			NO	metabo_info.php?molid=50993
93769	777.227879	[M+Na]+	754.232	8	Raphanusol A	C34H42O19	74565-72-3		NO	metabo_info.php?molid=93769
95268	777.227879	[M+Na]+	754.232	8	Kaempferide 3-[rhamnopyranosyl-(1->6)-glucoside] 7-rhamnoside	C34H42O19	150164-07-1		NO	metabo_info.php?molid=95268
95297	777.227879	[M+Na]+	754.232	8	3',6-Disinapoylsucrose	C34H42O19	139891-98-8		NO	metabo_info.php?molid=95297
1270	287.151814	[M+Na]+	264.163	0	Mianserin	C18H20N2	24219-97-4		YES	metabo_info.php?molid=1270
67371	287.151814	[M+Na]+	264.163	0	(-)-Apparicine	C18H20N2	2122-36-3	C09036	NO	metabo_info.php?molid=67371
66759	273.136431	[M+H]+	272.129	0	Procaine hydrochloride	C13H21ClN2O2	######	C07894	NO	metabo_info.php?molid=66759
67546	273.136431	[M+H]+	272.131	8	Sempervirine	C19H16N2	6882-99-1	C09240	NO	metabo_info.php?molid=67546
1271	273.136431	[M+Na]+	250.147	0	Desmethylmianserin	C17H18N2	71936-92-0		NO	metabo_info.php?molid=1271
73548	410.161775	[M+H]+	409.153	4	Acetylcorynoline	C23H23NO6	18797-80-3		YES	metabo_info.php?molid=73548
66912	410.161775	[M+H]+	409.158	9	Linustatin	C16H27NO11	72229-40-4	C08333	YES	metabo_info.php?molid=66912
69508	410.161775	[M+Na]+	387.171	2	Nemonapride	C21H26ClN3O2	75272-39-8	C12915	NO	metabo_info.php?molid=69508
85713	284.137711	[M+H]+	283.128	8	Glutaminyl-Histidine	C11H17N5O4			NO	metabo_info.php?molid=85713
85789	284.137711	[M+H]+	283.128	8	Histidinyl-Glutamine	C11H17N5O4			NO	metabo_info.php?molid=85789
85805	284.137711	[M+H]+	283.128	8	Histidinyl-Gamma-glutamate	C11H17N5O4			NO	metabo_info.php?molid=85805
86043	284.137711	[M+H]+	283.128	8	Gamma-glutamyl-Histidine	C11H17N5O4			NO	metabo_info.php?molid=86043
