# Supplementary material for: Phosphatidylcholine could protect the defect of zearalenone exposure on follicular development and oocyte maturation
Source: Aging (Albany NY). 2018 Nov 25;10(11):3486–506. doi: 10.18632/aging.101660 (PMC6286824; doi:10.18632/aging.101660)
Supplement: Supplementary Table 8 [file aging-10-101660-s007.pdf]

Table S8. Co-existing metabolites in each groups

Name	Fold change	Log2 |fold change|	P-value	Variation trend	Mass charge ratio (m/z)	Retention time (min)	Mean value in small follicle	Standard error in small follicle	Mean value in large follicle	Standard error in large follicle
M461T25	4.614702585	2.20623767	5.70E-12	UP	461.27842	24.7760833	907.08284	438.032783	4185.917538	483.360032
M438T25	3.582850782	1.84110796	1.27E-10	UP	438.29173	24.7765333	12588.262	5387.36038	45101.8659	5705.65835
M267T2	2.13509937	1.09430322	2.31E-09	UP	267.05539	1.67516667	12070.468	2270.14667	25771.64857	3008.89973
M460T25	4.149574906	2.05296355	2.82E-09	UP	460.27309	24.7815083	4050.1114	1644.78976	16806.2406	2749.2672
M439T25	4.482397223	2.1642705	4.96E-09	UP	439.29686	24.7701667	2455.5646	1861.09291	11006.81577	1819.21957
M280T1_4	2.134210578	1.09370253	3.73E-08	UP	280.08822	1.09149167	6206.1051	1870.94658	13245.13523	1398.23538
M266T1_3	3.543303957	1.82509523	7.94E-08	UP	266.07116	1.20978333	1228.089	612.961874	4351.492754	877.81857
M311T6_2	1.843541691	-0.88248	2.30E-07	DOWN	311.00437	5.74095833	6845.3246	941.987477	3713.137937	743.43013
M482T25_1	2.628789322	1.39439853	3.97E-07	UP	482.257	24.7797	1440.8759	419.10852	3787.759214	735.376334
M643T25_1	5.158933932	2.36707297	1.60E-06	UP	643.27769	24.61505	210.58372	430.476896	1763.154556	537.962541
M306T2_3	3.239980036	1.69598492	4.40E-06	UP	306.07744	1.98559167	1910.9138	520.645562	6191.32252	1540.71247
M467T29	2.997345021	1.58368516	1.03E-05	UP	467.3274	28.6889	2828.4806	780.960279	8477.93213	2233.66434
M137T2_2	1.225020269	0.29280562	1.44E-05	UP	137.04439	1.6481	25877.075	2145.94871	31699.94183	2274.42975
M311T2_2	1.445667906	0.53173618	4.77E-05	UP	311.03658	1.65601667	2938.0541	227.769562	4247.450526	614.124974
M327T1_5	1.296203443	0.37429217	4.87E-05	UP	327.04704	1.04054167	27758.191	3298.69894	35980.26217	3615.0503
M175T6_3	1.663770162	-0.7344561	5.01E-05	DOWN	175.03182	5.74925	28571.725	3737.00837	17172.87952	5408.32348
M468T21	2.242286916	-1.1649709	5.15E-05	DOWN	468.30217	21.0178167	52330.372	13521.329	23337.94641	4213.48493

M191T1_5	1.143155048	0.19302109	5.56E-05	UP	191.07392	1.0576	59491.106	3291.45314	68007.55832	3896.75362
M359T37_1	1.964340644	0.97404513	6.94E-05	UP	359.29097	37.3853167	1454.6172	472.911335	2857.363592	686.650936
M469T2 1	2.382307114	-1.2523594	8.16E-05	DOWN	469.3055	21.0129	13522.252	3739.58541	5676.116227	652.463936
M244T1_3	3.082282919	1.62399929	0.00018437	UP	244.08913	1.27443333	1297.2522	388.376195	3998.498414	1453.43002
M941T53_1	1.307741132	0.38707699	0.00019859	UP	940.80379	52.8026417	2889.7667	489.943211	3779.066714	302.175658
M295T2_1	1.297036465	0.37521904	0.00024368	UP	295.06236	1.6469	15187.487	2096.7038	19698.72401	2314.0616
M858T0_2	1.626671984	-0.7019234	0.00025593	DOWN	858.14753	0.31773333	2161.4176	375.024964	1328.735983	438.837999
M175T6_1	1.494231193	-0.5794034	0.00025809	DOWN	175.03195	6.43181667	72667.05	7769.35486	48631.73169	13710.3859
M496T24_2	1.476050348	-0.5617419	0.00047266	DOWN	496.33324	23.558625	287033.18	56735.607	194460.2938	19907.7984
M498T24_2	1.437492444	-0.5235544	0.00061344	DOWN	498.33916	24.16435	81315.456	15724.0092	56567.57074	6399.57782
M586T24_1	1.386148394	-0.4710817	0.00065362	DOWN	586.30134	24.1644	82952.287	14803.0502	59843.72744	6372.67054
M525T2 8	1.373617502	-0.4579803	0.00073501	DOWN	525.36746	28.13325	330489.26	57504.6993	240597.7334	34189.4899
M245T2_1	1.837479988	0.87772854	0.00074015	UP	245.07405	1.68074167	1420.9462	561.010067	2610.960182	724.410247
M580T2 4	1.376574141	-0.4610823	0.00082416	DOWN	580.2848	24.172725	3994.905	674.027957	2902.063067	520.327538
M284T2_2	3.14986468	1.65528985	0.00085278	UP	284.09575	1.97921667	1334.2914	459.628008	4202.837309	1883.32324
M268T2_1	1.856276697	0.89241178	0.00097821	UP	268.05844	1.67915	1498.1362	852.699733	2780.955366	488.710566
M497T24_2	1.440051216	-0.5261201	0.00109747	DOWN	497.33643	23.5586083	77201.937	16161.554	53610.54918	6011.81021
M531T1_5	1.282927855	0.35944004	0.00114148	UP	531.00627	1.0408	4571.3994	815.73183	5864.775688	662.45628
M490T2 1	1.938338909	-0.9548208	0.00118975	DOWN	490.28387	21.0030917	18246.161	6059.79426	9413.297472	1226.61494

M725T0_2	1.246646897	0.31805289	0.00119271	UP	724.86699	0.31768333	6809.2557	1156.34527	8488.737505	580.194617
M207T1_4	1.468719756	0.55455914	0.00129211	UP	207.01172	1.13938333	2312.6096	516.065953	3396.575401	717.969005
M518T24_2	1.448854329	-0.5349126	0.00132714	DOWN	518.31504	23.55685	113745.41	24772.8453	78507.14256	9067.6868
M263T53_1	1.5431048	0.62583605	0.00141476	UP	262.82854	52.61835	1248.077	412.315443	1925.913584	392.011449
M604T24_1	1.500087183	-0.5850464	0.00164111	DOWN	604.29417	24.1643	11935.955	2879.54828	7956.84061	906.890656
M147T1_3	1.084084906	0.11647775	0.0019732	UP	147.11099	1.02396667	33878.066	2043.06932	36726.69954	1286.47201
M612T2_5	1.356667619	-0.4400673	0.00234369	DOWN	612.31654	25.1564917	70851.293	14095.4789	52224.50334	6601.35078
M255T5_3	1.433860734	0.51990491	0.00264608	UP	255.22859	32.9247917	14754.956	4683.60015	21156.55216	3245.24359
M862T5_2	1.608258723	-0.6854995	0.00287398	DOWN	861.53696	31.5302667	2811.195	833.783583	1747.974372	331.092707
M497T24_1	1.427807644	-0.5138016	0.0030588	DOWN	497.33672	24.1643833	481908.3	114621.21	337516.2599	31736.2787
M496T24_1	1.476428458	-0.5621115	0.00312472	DOWN	496.33363	24.16435	2024389.1	519632.088	1371139.3	189287.939
M524T28_2	1.317201595	-0.3974762	0.00341202	DOWN	524.36423	28.124725	1185410.3	228857.489	899945.9882	100015.446
M854T0_1	1.359186104	0.44274301	0.00345006	UP	853.50155	0.30629167	1019.8711	271.430013	1386.194623	205.677082
M159T2	1.134429653	0.18196715	0.00365079	UP	159.02641	1.64256667	26197.701	2685.78333	29719.44922	1879.39973
M780T1_4	1.167193827	-0.2230442	0.00365687	DOWN	780.43456	1.12396667	8747.4261	989.757701	7494.407461	574.654591
M805T53_2	1.317784941	0.39811495	0.00433475	UP	804.83188	52.8025833	9579.5923	2543.01989	12623.84252	984.895291
M546T28_1	1.329005599	-0.4103472	0.00490725	DOWN	546.34574	28.133	468358.37	99012.1127	352412.6376	35049.6074
M671T2_4	1.404453223	-0.4900086	0.00506608	DOWN	671.27837	24.1643667	7382.2716	1756.95495	5256.331444	1037.50102
M307T5_2	1.103454817	0.14202756	0.00541885	UP	306.89068	52.886975	79168.64	7027.08227	87359.01693	3229.31203
M1255T0	1.603752439	-0.6814515	0.00628691	DOWN	1255.2398	0.314625	766.34671	200.160473	445.0501369	257.326385
M587T2_4	1.489933537	-0.575248	0.00671466	DOWN	587.30492	24.1807667	23671.867	6889.35571	15887.86792	3295.45074

M709T1_4	1.120033511	0.1635419	0.00694411	UP	708.81033	1.04078333	7424.0282	665.293505	8315.160361	642.415848
M163T5_[illegible]	1.189312293	0.25012759	0.00704516	UP	162.90503	52.6655417	6370.8543	946.829023	7576.935358	818.043995
M615T53_2	1.503197855	-0.5880349	0.00779086	DOWN	614.87866	52.7992333	2071.9779	587.351796	1378.379993	422.474565
M482T2_3	1.581705207	-0.6614807	0.0079568	DOWN	482.31803	22.5300333	42086.93	14424.0444	26608.58057	3046.21394
M739T2_4	1.334880168	-0.4167102	0.00846064	DOWN	739.26607	24.1643833	3309.6158	659.794908	2479.335509	592.941081
M603T24_1	1.288528022	-0.3657239	0.00846356	DOWN	603.29113	24.1643083	41328.142	8022.88644	32073.91756	5543.44118
M611T1_1	1.108831923	-0.1490407	0.00847053	DOWN	610.55551	1.12396667	3739.8175	212.659866	3372.754221	321.558417
M599T1_6	1.2513531	0.32348894	0.00887449	UP	598.99447	1.03411667	2586.6121	465.960079	3236.765122	522.026106
M518T24_1	1.43250923	-0.5185444	0.00898181	DOWN	518.31502	24.1643667	840566.42	240435.285	586779.0571	79307.1105
M504T2_3	1.552107276	-0.6342283	0.01013362	DOWN	504.29953	22.5308667	17961.459	6208.23665	11572.30512	1722.74282
M681T1_4	1.160519169	0.21477035	0.01028001	UP	680.86113	1.0395	2468.5501	306.298097	2864.799692	312.043043
M759T0_2	1.187273238	0.24765199	0.01043305	UP	758.85989	0.31153333	7393.0342	1328.80683	8777.551594	584.378752
M522T25_3	1.362853532	-0.4466305	0.01114223	DOWN	522.34874	25.1564833	1743586.2	459794.256	1279364.336	114171.246
M654T2_4	1.390524145	-0.4756288	0.01177484	DOWN	654.28828	24.1643833	14149.599	3796.52161	10175.73078	2115.92803
M523T25_2	1.302177359	-0.380926	0.01209212	DOWN	523.35178	25.1565	455449.28	105100.914	349759.7869	39956.5793
M243T1_1	1.100228817	0.1378036	0.01221825	UP	242.92233	1.0544	11810.659	683.607206	12994.42753	1121.02671
M739T53_3	1.325448513	0.40648063	0.01247334	UP	738.83948	52.7857333	4929.6803	1609.65576	6534.037446	570.814509
M443T53_2	1.181040727	0.24005872	0.01253275	UP	442.86373	52.8867167	56263.214	10151.9533	66449.14702	4167.39076
M519T24_2	1.369881838	-0.4540515	0.01263361	DOWN	519.31822	24.164325	201344.25	54630.3076	146979.2862	19751.2398
M291T2	1.326652446	0.40779047	0.01366073	UP	291.06607	2.01795	6886.2736	1122.12286	9135.691651	2237.8491

M544T25_1	1.286342869	-0.3632752	0.01390482	DOWN	544.33018	25.1564833	683557.59	156569.361	531396.1062	48371.8604
M241T53_1	1.14962812	0.20116726	0.01411474	UP	240.89953	52.8894333	9617.9278	1441.52316	11057.04025	717.514375
M667T1_1	1.133571881	-0.1808759	0.01449192	DOWN	666.5158	1.122675	4153.4006	415.669095	3663.994026	392.816054
M873T53_3	1.231408132	0.300309	0.01605651	UP	872.8181	52.8026417	5575.3337	1337.52924	6865.511318	601.787398
M722T24	1.447647505	-0.5337104	0.01738146	DOWN	722.274	24.164375	6657.5574	2154.10603	4598.880139	1029.60165
M311T6_1	1.406879863	-0.4924991	0.01864958	DOWN	311.0049	6.42211667	17769.217	3153.78007	12630.23036	5275.71268
M444T53	1.191788129	0.25312778	0.01985745	UP	443.86603	52.8869667	4827.3591	998.588872	5753.189258	457.995437
M429T1_6	1.39111193	-0.4762385	0.02063604	DOWN	428.89508	0.98995833	1262.9897	392.160431	907.8994377	146.097297
M688T24	1.301379088	-0.3800413	0.02109376	DOWN	688.26951	24.1559917	5694.6163	1457.75125	4375.832052	575.571417
M701T53_1	1.64740367	0.72019411	0.02160028	UP	700.75927	52.5401167	801.21647	562.321808	1319.926955	285.815475
M522T25_2	1.263882275	-0.3378621	0.02185397	DOWN	522.34791	24.5676167	261880.83	56032.671	207203.4974	38658.4411
M261T2_1	1.220519664	0.28749554	0.02195715	UP	261.03984	2.05058333	10031.926	2036.72296	12244.16313	1904.75054
M827T0_3	1.155513274	0.20853383	0.02205226	UP	826.84632	0.31459167	5686.4569	924.308165	6570.776481	592.48573
M661T53_2	1.348514564	0.4313711	0.02206854	UP	660.79245	52.7688	4221.0777	1153.31857	5692.184767	1444.50037
M929T0_1	1.138191548	0.18674337	0.02451341	UP	928.82534	0.31459167	3444.4068	477.845749	3920.394736	379.810859
M1111T53	1.42252979	0.50845887	0.02548177	UP	1110.773	52.8007417	1167.5073	459.712956	1660.813851	445.833465
M545T25_2	1.2796423	-0.3557406	0.02678993	DOWN	545.33334	25.1565583	190980.46	49186.9546	149245.1902	14972.9873
M568T39_2	1.715047396	0.77824845	0.02760282	UP	568.35156	39.307825	1020.4018	789.667091	1750.037439	528.91321

M568T39_1	1.502642374	0.58750169	0.02915529	UP	568.01686	39.3161667	1436.4791	833.993532	2158.514351	420.842667
M285T53_1	1.269833988	0.3446399	0.02996503	UP	284.81041	52.6331583	5167.1357	1684.3301	6561.404587	518.243259
M287T1_6	1.180526277	0.23943015	0.03190768	UP	287.06579	1.05443333	2288.9956	275.651614	2702.2195	476.154536
M784T1_4	1.108568022	-0.1486973	0.03198323	DOWN	784.42922	1.1384	8037.6134	707.79643	7250.446781	801.635846
M160T2_1	1.172362412	0.22941862	0.03207291	UP	160.02883	1.65583333	1603.6926	208.438902	1880.108868	307.986108
M426T1_3	1.318569505	-0.3989736	0.03307575	DOWN	425.80414	1.16233333	2889.3418	812.080751	2191.269983	466.020278
M377T1_2	1.217418656	-0.2838254	0.033148	DOWN	376.7272	1.13949167	11198.897	2484.30621	9198.887034	670.777843
M237T3[illegible]	1.625111945	0.7005391	0.03320631	UP	237.21807	32.9207667	10312.462	4290.90467	16758.90543	7512.23533
M242T4	1.080236991	0.11134786	0.03376318	UP	242.09663	3.98318333	72247.361	5677.29986	78044.27164	5603.56813
M861T0_2	1.156488706	0.20975118	0.03828093	UP	860.83968	0.31763333	5037.692	941.421119	5826.033892	559.319018
M305T0_2	1.040551243	0.05734801	0.04159559	UP	305.1531	0.31765833	392315.72	9447.06985	408224.6108	20107.1202
M936T5[illegible]	1.211430635	0.2767118	0.04287144	UP	935.79329	52.777325	1680.3338	439.516889	2035.607792	249.442675
M510T2[illegible]6	1.337046948	-0.4190501	0.04585567	DOWN	510.34854	26.0813833	75704.943	25563.1079	56621.00577	7687.33132
M373T0[illegible]	1.461848093	-0.5477934	0.04716328	DOWN	373.21498	0.31156667	2832.648	512.969005	1937.717066	1176.34877
M551T1_1	1.127478544	-0.1731	0.04807435	DOWN	550.60024	1.13118333	7878.5	403.477847	6987.716089	1201.03033
M534T2[illegible]4	1.320445077	-0.4010243	0.04813737	DOWN	534.28743	24.1643167	13370.822	4341.5156	10125.99607	1645.59178
M239T5[illegible]	1.07402569	0.1030285	0.04834812	UP	238.90436	52.886975	25448.276	2387.19358	27332.10234	1391.36445

Name	Fold change	Log2 |fold change|	P-value	Variation trend	Mass charge ratio (m/z)	retention time (min)	Mean value in small follicle	Standard error in small follicle	Mean value in large follicle	Standard error in large follicle
M461T25	3.23130523	-1.692117034	9.09E-06	DOWN	461.281565	24.6939333	958.48161	305.0900111	215.0080564	182.7404054
M438T25	3.23742839	-1.694848283	1.61E-09	DOWN	438.293742	24.6938167	10159.942	1502.303132	3138.275527	1193.597655
M267T2	1.31650137	-0.396709025	0.001269	DOWN	267.056411	1.66485833	21962.761	2659.345149	16682.67263	3416.833016
M460T25	3.36155478	-1.749128658	4.44E-06	DOWN	460.277661	24.710175	4513.7024	1199.413265	1342.742484	570.5900511
M439T25	3.22318759	-1.688488155	1.42E-06	DOWN	439.300311	24.6932667	2222.0997	507.5625161	689.4105988	459.673094
M280T1	1.95982394	-0.970724056	0.000246	DOWN	280.089569	1.08373333	8786.2897	2032.375724	4483.20357	2185.628412
M266T1_2	1.38503516	-0.469922596	0.001439	DOWN	266.07223	1.187025	10201.209	1587.308439	7365.306558	1774.591373
M311T6_2	2.72213405	1.444738114	2.39E-06	UP	311.004613	5.74460833	459.26784	100.6343218	1250.188626	271.3746702
M482T25	2.67313371	-1.418532003	4.10E-06	DOWN	482.260473	24.6933833	1061.2867	303.2651222	233.7884562	258.8649059
M643T25	2.17532536	-1.121231199	3.13E-07	DOWN	643.280445	24.64265	5564.0548	894.2404035	2557.803485	803.8385609
M306T2	1.61435329	-0.69095634	4.77E-06	DOWN	306.078134	1.95729167	10075.345	1457.69173	6241.102778	684.1752955
M467T29	2.36273845	-1.240459934	0.000712	DOWN	467.331054	28.5948833	2699.5085	1007.861666	1142.533793	393.4307007
M137T2	1.42674542	-0.512727935	2.72E-05	DOWN	137.045185	1.60654167	28037.315	3582.838474	19651.23858	3076.342531
M311T2_2	1.44488864	-0.530958304	0.003358	DOWN	311.038269	1.61005833	1658.0845	350.0514479	1147.551731	325.0272897
M327T1_3	1.13461126	-0.182198091	0.035251	DOWN	327.048772	1.030675	13505.45	1227.850879	11903.15163	1825.482606
M175T6_2	2.08941779	1.063100995	1.18E-05	UP	175.032578	5.74408333	1551.9227	490.3585671	3242.614946	704.2972631
M468T21	1.64865255	0.721287386	0.000206	UP	468.303469	20.9443167	3851.8532	911.657815	6350.367669	1368.542249

M191T1_4	1.16722141	-0.223078247	0.001007	DOWN	191.074914	1.02766667	11194.004	889.9528406	9590.300042	938.6125314
M359T37	2.56426949	-1.358547886	0.012215	DOWN	359.292536	37.4777667	1108.3607	677.3033957	432.2325239	225.1945257
M469T21	1.7525421	0.809449105	0.003727	UP	469.305624	20.9299167	695.46654	228.1132909	1218.834385	417.7842358
M244T1_2	1.49879222	-0.583800396	0.008415	DOWN	244.088986	1.201575	4089.0959	1267.448916	2728.260703	491.3612681
M941T53_1	1.1265954	-0.171969483	4.03E-05	DOWN	940.811676	52.78105	14462.016	572.4787141	12836.92063	740.1790188
M295T2	1.43099021	-0.517013801	3.86E-05	DOWN	295.063627	1.60578333	35261.989	4928.751435	24641.67012	3353.472184
M858T0_2	4.92536558	2.300230812	0.00027	UP	858.146473	0.31025	377.57788	218.644556	1859.709119	836.1047393
M175T6_1	2.28107904	1.189716437	2.07E-05	UP	175.032713	6.43848333	2690.0537	753.9562263	6136.225206	1516.605228
M496T23	4.42110189	2.144405984	4.09E-05	UP	496.334547	23.4835167	13781.516	4162.763533	60929.48478	20763.04757
M498T24	4.62328324	2.20891775	0.000102	UP	498.340881	24.1051833	4085.2371	2033.903014	18887.20809	7415.220683
M586T24	3.43935182	1.782136699	1.19E-06	UP	586.303372	24.1184667	8380.246	3246.790961	28822.61444	6844.809602
M525T28	4.04292452	2.015399268	0.000308	UP	525.368045	28.09115	10325.523	4129.635865	41745.30821	17940.96463
M245T2	1.48653584	-0.571954243	0.000856	DOWN	245.075394	1.655575	1302.1583	211.4754941	875.968302	259.8102549
M580T24	2.89599312	1.534058174	5.69E-05	UP	580.284352	24.1307833	682.65283	294.2437435	1976.957909	628.8292803
M284T2	1.65971727	-0.730937501	1.74E-05	DOWN	284.096265	1.957375	6926.1871	1171.518969	4173.112625	482.9137846
M268T2	1.38648327	-0.471430207	0.002236	DOWN	268.059644	1.66585833	2435.428	504.375514	1756.550589	275.9349928
M497T23	3.75315671	1.908104531	4.58E-05	UP	497.33659	23.4835833	4309.4936	1003.930642	16174.20494	5282.954862
M531T1_3	1.1364074	-0.184480131	0.049246	DOWN	531.009751	1.03328333	6710.1622	519.8875064	5904.715349	1054.478382
M490T21	1.76389613	0.818765606	0.006103	UP	490.286388	20.9443833	2158.8441	855.0336402	3807.976733	1392.911864

M725T0_3	1.11072469	-0.15150127	0.047523	DOWN	724.87192	0.31025	3811.2795	423.3701265	3431.344852	373.0265474
M207T1_2	1.34124455	-0.42357231	0.003645	DOWN	207.012503	1.09511667	2801.1886	567.8373473	2088.499483	306.4599853
M518T23	3.86372703	1.949993172	0.000218	UP	518.316319	23.48355	8402.8418	3027.494574	32466.28713	13137.89228
M263T53_1	2.00624636	-1.004498772	0.000649	DOWN	262.83114	52.6473667	778.12683	243.7418541	387.8520839	155.9544091
M604T24	4.38631777	2.133010332	0.000347	UP	604.295239	24.0968833	1233.9651	588.0796637	5412.562966	2428.303524
M147T1_2	1.38138898	-0.46611962	0.002151	DOWN	147.111743	1.00316667	4241.4315	818.7696716	3070.410711	610.6501217
M612T25	3.6112297	1.852490188	8.68E-05	UP	612.318647	25.1133333	2476.5315	888.2397216	8943.32412	3181.015425
M255T[illegible]_2	1.26711544	-0.34154797	0.01929	DOWN	255.230351	32.9996	4007.7145	677.4708828	3162.864532	785.7678804
M862T31	1.45627929	0.542287069	0.01079	UP	861.543906	31.4634583	3387.9609	1306.844451	4933.817349	1109.387533
M497T24	3.51539851	1.813688244	6.12E-05	UP	497.33868	24.1184167	30827.342	6759.33263	108370.3917	35772.02891
M496T24	3.68252652	1.880695913	0.000112	UP	496.335073	24.1147333	113866.93	26199.47532	419317.9824	151498.9375
M524T28	5.79343933	2.534420073	2.77E-05	UP	524.366042	28.093375	33910.995	18526.11276	196461.2909	69676.63654
M854T0_1	1.2823324	-0.358770283	0.023655	DOWN	853.504447	0.31018333	1138.9861	249.7785707	888.2144206	199.0018091
M159T2	1.3139771	-0.39394013	5.95E-05	DOWN	159.026456	1.6065	39540.255	4629.274854	30092.04262	2484.88415
M780T1_3	1.67726967	0.746114661	0.001571	UP	780.441396	1.09085	949.76896	276.3106086	1593.018667	449.712086
M805T53_3	1.05608289	-0.078723071	0.010466	DOWN	804.838631	52.78105	38434.726	1559.40145	36393.66414	1633.521163
M546T28	11.0026274	3.459776167	1.14E-06	UP	546.347535	28.0907833	9727.6606	8379.46432	107478.8178	29734.75991
M671T24	3.82967292	1.937221182	0.000787	UP	671.277944	24.0975833	694.2693	439.9629954	2815.104876	1385.914535
M307T53	1.06546872	-0.091488245	0.014676	DOWN	306.892084	52.8634167	45280.759	2586.701115	42498.44013	1938.872753
M1255T0	1.70865927	0.772864735	0.006398	UP	1255.24246	0.310225	363.52545	277.8140062	690.8677735	172.8458129
M587T24	3.49786838	1.806476003	0.000119	UP	587.305573	24.0972667	2401.1838	903.8869734	8399.024798	3073.077754

M709T1	1.25753393	-0.330597323	0.001139	DOWN	708.803857	1.06445	5993.3697	733.6891789	4765.970594	685.0520519
M163T53	1.18335487	-0.242882785	0.011136	DOWN	162.905781	52.6799167	6913.8787	1044.638883	5842.608058	431.3331546
M615T53_2	1.2970096	0.375189157	0.038215	UP	614.8747	52.8313333	841.62622	269.2113085	1091.597283	227.8328557
M482T22	3.93509131	1.976397113	0.000827	UP	482.318641	22.4557833	1010.3412	471.4775672	3975.785036	1938.5914
M739T24	4.9986033	2.321525036	0.000159	UP	739.265128	24.1143	209.31361	129.9481962	1250.257717	546.5918523
M603T24	4.26440129	2.092343204	4.06E-05	UP	603.291822	24.1052	4752.4845	1813.613491	20266.50119	6942.950142
M611T1	1.49497186	0.58011833	0.002922	UP	610.55931	1.09733333	730.25097	200.0235114	1091.70465	261.0232532
M599T1_2	1.18623571	-0.246390713	0.021542	DOWN	598.996292	1.030975	4029.297	419.0748957	3396.708531	659.4327508
M518T24	3.78839263	1.921585858	7.81E-05	UP	518.316795	24.1148333	60435.668	18031.97943	228954.0381	80768.47132
M504T22	3.44589432	1.784878455	9.76E-05	UP	504.300209	22.4557833	712.68613	397.3640915	2596.460482	963.6360572
M681T1	1.19751908	-0.260048641	0.00019	DOWN	680.863465	1.03038333	2516.536	227.2438205	2101.457948	142.8110334
M759T0_2	1.18763838	-0.248095626	0.000421	DOWN	758.864846	0.31026667	4498.7244	338.5017702	3787.958031	393.3123485
M522T25_1	3.60066955	1.848265201	9.66E-06	UP	522.350481	25.1158917	32088.501	9688.982247	115540.087	32180.69213
M654T24	3.99554834	1.998393508	0.00076	UP	654.288327	24.1138667	1098.7946	498.8997559	4390.287039	2124.633649
M523T25	3.83525564	1.939322746	2.40E-05	UP	523.35295	25.113175	8841.5727	2460.263921	33909.69164	10468.7342
M243T1	1.11499824	-0.157041429	0.002291	DOWN	242.923459	1.05013333	160883.48	12067.27387	144290.3403	7848.48215
M739T53_3	1.19338756	-0.255062641	0.018408	DOWN	738.83766	52.78105	2327.152	271.8114733	1950.038748	366.4679041
M443T53	1.07002169	-0.097640045	0.044519	DOWN	442.865912	52.8476917	40538.922	3477.3327	37886.07451	1332.69052
M519T24	3.76115798	1.911176906	0.000127	UP	519.319936	24.1147833	16228.127	4940.129513	61036.54957	22789.6957
M291T2	1.77233696	-0.825652915	0.000201	DOWN	291.067387	1.9844	52839.059	12382.43556	29813.21312	2538.535328

M544T25_1	3.9445078	1.979845289	1.48E-05	UP	544.332078	25.11405	16874.71	5742.142004	66562.42337	19983.28917
M241T53_1	1.11811955	-0.16107445	0.010076	DOWN	240.900173	52.864525	6959.1066	422.4393945	6223.937899	669.164766
M667T1_1	1.46553507	0.551427493	0.019356	UP	666.520425	1.09598333	765.42015	220.8604328	1121.750076	368.1802112
M873T53_3	1.05581907	-0.078362624	0.037698	DOWN	872.82553	52.78105	23876.708	1304.523005	22614.39329	1208.994038
M722T24	3.74691892	1.90570476	0.000199	UP	722.27494	24.1144417	522.21766	298.988431	2109.610765	873.3265282
M311T6_1	2.48687664	1.314334947	0.000644	UP	311.004921	6.43845	968.47943	374.4748833	2408.488867	920.5136393
M444T53	1.12412141	-0.168797866	0.028782	DOWN	443.868254	52.847875	3664.6895	417.6496526	3260.047771	336.4890483
M429T1_3	1.20230008	0.265797022	0.046952	UP	428.891755	1.035125	508.4879	93.76709341	611.35504	119.4781011
M688T24	5.37668041	2.42671572	0.001283	UP	688.268968	24.0930667	476.84431	322.594336	2783.250001	1599.783987
M701T53_1	1.3624848	-0.44624013	0.000286	DOWN	700.762927	52.59805	2369.0837	184.5030213	1738.796423	365.3560248
M522T25_2	12.1561844	3.603618558	8.35E-07	UP	522.349105	24.5079167	1656.3537	3399.147482	21127.07528	6428.225714
M261T2_1	1.3997229	-0.48514125	0.0188	DOWN	261.041761	2.046725	960.89837	224.7641498	686.4918529	249.395504
M827T0_3	1.16892004	-0.225176243	0.007996	DOWN	826.85034	0.31025	3742.638	355.6568115	3201.791308	445.5976975
M661T53_2	1.1023284	-0.140554087	0.037034	DOWN	660.795452	52.7807833	8937.0084	786.0232463	8107.392005	858.7292571
M929T0_1	1.20665263	-0.271010418	0.007363	DOWN	928.82837	0.31023333	2717.1785	286.8033222	2251.831547	387.5305038
M1111T53_1	1.12838117	-0.174254492	0.001158	DOWN	1110.77742	52.7810333	6158.3906	436.5748651	5457.721918	369.1689839
M545T25	3.71149032	1.891998608	5.66E-05	UP	545.333276	25.1132833	4768.2765	1370.223024	17697.41201	5967.34641
M568T39_2	1.8507178	-0.888084927	0.028317	DOWN	568.356297	39.20925	1287.6294	677.9198351	654.9381177	480.8375783

M568T39_1	1.5319928	-0.61540952	0.040726	DOWN	568.021159	39.22485	1531.0118	565.0379986	999.359668	511.2293815
M285T53_1	1.56400366	-0.64524389	0.003873	DOWN	284.811225	52.6480833	4056.5299	1196.858846	2593.683138	508.4667329
M287T1	1.26372986	-0.337688096	0.024611	DOWN	287.067139	1.047275	1948.8239	433.2956259	1542.120606	281.1126254
M784T1_3	1.55849712	0.640155484	0.009345	UP	784.435552	1.09388333	933.31218	389.3115641	1454.564337	411.2301641
M160T2	1.26715353	-0.341591337	0.030778	DOWN	160.029208	1.60506667	2406.9056	547.515901	1899.458508	402.5155526
M426T1_1	1.34143205	0.423773973	0.004245	UP	425.804824	1.10116667	529.88091	117.4824248	710.7992353	129.274465
M377T1_1	1.6101264	0.687173948	0.002545	UP	376.728869	1.10105	1083.6053	283.0843326	1744.741501	497.736875
M237T33_2	1.21949202	-0.286280322	0.037766	DOWN	237.219761	32.98315	2448.4508	262.1575141	2007.762834	542.5127123
M242T4	1.39976776	-0.485187482	0.000766	DOWN	242.097842	3.9925	2343.6564	396.5138395	1674.318043	338.3000408
M861T0_3	1.16657616	-0.22228049	0.024688	DOWN	860.84683	0.31025	3446.8886	517.4036796	2954.705137	355.1551396
M305T0_2	1.05296035	-0.074451105	0.004658	DOWN	305.154618	0.31025	479052.95	13916.74257	454958.2069	18721.04381
M936T33_1	1.10102407	-0.138846012	0.000519	DOWN	935.798972	52.7643167	6795.239	333.3956286	6171.744291	327.897711
M510T26	33.4709342	5.064836914	4.00E-05	UP	510.349044	26.0069417	25.853375	46.23380882	10426.46437	4431.204242
M373T0	1.19521859	0.257274487	0.024225	UP	373.217792	0.31015	1047.7713	139.496488	1252.315737	217.891313
M551T1	1.47344152	0.559189798	0.009567	UP	550.602003	1.10053333	1412.1345	453.9663847	2080.697565	565.640977
M534T24	3.98135683	1.993260181	0.000305	UP	534.289451	24.1136333	987.41029	425.1133294	3931.232718	1684.862363
M239T33_2	1.14749869	-0.198492512	0.000129	DOWN	238.905028	52.8632417	16856.52	796.6001746	14689.7945	1119.69553
