# Supplementary material for: Phosphatidylcholine could protect the defect of zearalenone exposure on follicular development and oocyte maturation
Source: Aging (Albany NY). 2018 Nov 25;10(11):3486–506. doi: 10.18632/aging.101660 (PMC6286824; doi:10.18632/aging.101660)
Supplement: Supplementary Table 9 [file aging-10-101660-s008.pdf]

Table S9 Prediction structures of the co-exist metabolites

Mol_ID	Input mass	Adduct	Mass	D-ppm	Name	Formula	CAS	KEGG	MS/MS	Link
72929	238.90503	[M+Na]+	215.915	4	3-Chloro-4-(dichloromethyl)-5-hydroxy-2(5H)-furanone	C5H3Cl3O3	77439-76-0	C19205	NO	metabo_info.php?molid=72929
46719	438.29374	[M+H]+	437.291	9	PE(P-16:0/0:0)	C21H44NO6P			NO	metabo_info.php?molid=46719
69329	438.29374	[M+Na]+	415.309	9	Myxalamid A	C26H41NO3	86934-09-0	C12158	NO	metabo_info.php?molid=69329
2441	267.05641	[M+H]+	266.047	6	5-Hydroxysulfadiazine	C10H10N4O3S	70800-61-2		NO	metabo_info.php?molid=2441
90	267.05641	[M+Na]+	244.07	8	Uridine	C9H12N2O6	58-96-8	C00299	YES	metabo_info.php?molid=90
5734	267.05641	[M+Na]+	244.07	8	Pseudouridine	C9H12N2O6		C02067	NO	metabo_info.php?molid=5734
45418	460.27766	[M+H]+	459.273	6	BW A868C	C25H37N3O5	118675-50-6		YES	metabo_info.php?molid=45418
46719	460.27766	[M+Na]+	437.291	4	PE(P-16:0/0:0)	C21H44NO6P			NO	metabo_info.php?molid=46719
73320	439.30031	[M+Na]+	416.308	7	all-trans-8\'-Apo-beta-carotenal	C30H40O	1107-26-2	C19728	NO	metabo_info.php?molid=73320
73390	439.30031	[M+Na]+	416.308	7	4,4\'-Diaponeurosporen-4-al	C30H40O		C19839	NO	metabo_info.php?molid=73390
93622	439.30031	[M+Na]+	416.308	7	Apo-8'-lycopenal	C30H40O	2213-22-1		NO	metabo_info.php?molid=93622
93820	280.08957	[M+Na]+	257.101	2	Pyro-L-glutaminyl-L-glutamine	C10H15N3O5	109481-23-4		NO	metabo_info.php?molid=93820
1290	280.08957	[M+Na]+	257.101	2	Benserazide	C10H15N3O5	322-35-0		YES	metabo_info.php?molid=1290
5919	280.08957	[M+Na]+	257.101	2	5-Methylcytidine	C10H15N3O5	2140-61-6		YES	metabo_info.php?molid=5919

63346	280.08957	[M+Na]+	257.103	8	sn-glycero-3-Phosphocholine	C8H20NO6P		C00670	NO	metabo_info.php?molid=63346
370	280.08957	[M+Na]+	257.103	8	Glycerophosphocholine	C8H20NO6P	28319-77-9		YES	metabo_info.php?molid=370
70539	266.07223	[M+Na]+	243.081	5	4-Chloro-[2-(4-Pyridinyl)-1-butenyl]phenol	C15H14ClN		C15045	NO	metabo_info.php?molid=70539
567	266.07223	[M+Na]+	243.086	9	Cytarabine	C9H13N3O5	147-94-4	C02961	YES	metabo_info.php?molid=567
3376	266.07223	[M+Na]+	243.086	9	Cytidine	C9H13N3O5	65-46-3	C00475	YES	metabo_info.php?molid=3376
63617	266.07223	[M+Na]+	243.086	9	γ-Glutamyl-β-cyanoalanine	C9H13N3O5		C05711	NO	metabo_info.php?molid=63617
36239	482.26047	[M+H]+	481.25	7	N-acetyl-LTE4	C25H39NO6S		C11361	YES	metabo_info.php?molid=36239
45418	482.26047	[M+Na]+	459.273	4	BW A868C	C25H37N3O5	118675-50-6		YES	metabo_info.php?molid=45418
46748	643.28044	[M+Na]+	620.296	7	PI(20:4(5Z,8Z,11Z,14Z)/0:0)	C29H49O12P			NO	metabo_info.php?molid=46748
67620	306.07813	[M+H]+	305.069	6	Atherospermidine	C18H11NO4	3912-57-0	C09347	NO	metabo_info.php?molid=67620
89877	306.07813	[M+H]+	305.069	6	Cepharadione A	C18H11NO4	55610-01-0		NO	metabo_info.php?molid=89877
2417	306.07813	[M+H]+	305.074	9	Sertraline	C17H17Cl2N	79617-96-2	C07246	YES	metabo_info.php?molid=2417
87	306.07813	[M+Na]+	283.092	8	Guanosine	C10H13N5O5	118-00-3	C00387	YES	metabo_info.php?molid=87
66971	306.07813	[M+Na]+	283.092	8	Isoguanosine	C10H13N5O5	1818-71-9	C08432	NO	metabo_info.php?molid=66971
44821	306.07813	[M+Na]+	283.092	9	8-hydroxy-2\'-deoxy Guanosine	C10H13N5O5	NA		YES	metabo_info.php?molid=44821

42251	467.33105	[M+H]+	466.326	4	24,24-difluoro-1α,25-dihydroxy-24a-homovitamin D3 / 24,24-difluoro-1α,25-dihydroxy-24a-	C28H44F2O3			NO	metabo_info.php?molid=42251
35473	137.04518	[M+H]+	136.037	5	D-threonic acid	C4H8O5			NO	metabo_info.php?molid=35473
35474	137.04518	[M+H]+	136.037	5	DL-erythronic acid	C4H8O5			NO	metabo_info.php?molid=35474
4244	137.04518	[M+H]+	136.037	5	Threonate	C4H8O5	70753-61-6	C01620	YES	metabo_info.php?molid=4244
45855	137.04518	[M+H]+	136.037	5	Erythronic acid	C4H8O5			NO	metabo_info.php?molid=45855
45859	137.04518	[M+H]+	136.037	5	Threonic acid	C4H8O5			NO	metabo_info.php?molid=45859
87453	137.04518	[M+H]+	136.038	0	1-Pentanesulfenothioic acid	C5H12S2	86849-52-7		NO	metabo_info.php?molid=87453
88945	137.04518	[M+H]+	136.038	0	Ethyl propyl disulfide	C5H12S2	30453-31-7		NO	metabo_info.php?molid=88945
88946	137.04518	[M+H]+	136.038	0	Ethyl isopropyl disulfide	C5H12S2	53966-36-2		NO	metabo_info.php?molid=88946
83	137.04518	[M+H]+	136.039	4	Hypoxanthine	C5H4N4O	68-94-0	C00262	YES	metabo_info.php?molid=83
865	137.04518	[M+H]+	136.039	4	allopurinol	C5H4N4O	315-30-0	C06816	YES	metabo_info.php?molid=865
70053	311.03827	[M+H]+	310.032	3	Diflubenzuron	C14H9ClF2N2O2	35367-38-5	C14427	YES	metabo_info.php?molid=70053
2913	311.03827	[M+Na]+	288.047	7	N-1-Desalkylflurazepam	C15H10ClFN2O	2886-65-9		YES	metabo_info.php?molid=2913
69954	311.03827	[M+Na]+	288.049	1	Furamizole	C12H8N4O5	17505-25-8	C14304	NO	metabo_info.php?molid=69954
68415	327.04877	[M+H]+	326.039	6	Gliotoxin	C13H14N2O4S2	67-99-2	C10595	NO	metabo_info.php?molid=68415

48347	327.04877	[M+H]+	326.043	3	2-Hydroxyflemichappari n C	C17H10O7			NO	metabo_info.php?molid=48347
48348	327.04877	[M+H]+	326.043	3	Tephrosol	C17H10O7			NO	metabo_info.php?molid=48348
48351	327.04877	[M+H]+	326.043	3	Sophoracoumestan B	C17H10O7			NO	metabo_info.php?molid=48351
1384	327.04877	[M+Na]+	304.058	6	Brompheniramine (monodemethylated)	C15H17BrN2	18453-10-6		NO	metabo_info.php?molid=1384
44402	327.04877	[M+Na]+	304.058	3	DIHYDROROBINETIN	C15H12O7	4382-33-6		YES	metabo_info.php?molid=44402
3434	327.04877	[M+Na]+	304.058	3	(±)-Taxifolin	C15H12O7	480-18-2	C01617	YES	metabo_info.php?molid=3434
3435	327.04877	[M+Na]+	304.058	3	Pentahydroxyflavanon e	C15H12O7	81398-31-4	C05911	NO	metabo_info.php?molid=3435
52482	327.04877	[M+Na]+	304.058	3	Nigrescin	C15H12O7			NO	metabo_info.php?molid=52482
52488	327.04877	[M+Na]+	304.058	3	Alphitonin	C15H12O7			NO	metabo_info.php?molid=52488
52937	327.04877	[M+Na]+	304.058	3	5,7,3\,4\,5\-Pentahydroxyflavanon e	C15H12O7			NO	metabo_info.php?molid=52937
95109	327.04877	[M+Na]+	304.058	3	Pratenol B	C15H12O7	147710-51-8		NO	metabo_info.php?molid=95109
94193	175.03258	[M+H]+	174.025	0	Brassilexin	C9H6N2S	119752-76-0		NO	metabo_info.php?molid=94193
55	175.03258	[M+Na]+	152.044	4	Cystamine	C4H12N2S2	56-17-7		YES	metabo_info.php?molid=55
64499	191.07491	[M+H]+	190.066	6	2-Oxo-7-methylthioheptanoic acid	C8H14O3S		C17220	NO	metabo_info.php?molid=64499
88307	191.07491	[M+H]+	190.066	6	Ethyl 4-(acetylthio)butyrate	C8H14O3S	104228-51-5		NO	metabo_info.php?molid=88307
42731	359.29254	[M+H]+	358.287	5	5β-Chol-9(11)-en-24-oic Acid	C24H38O2			NO	metabo_info.php?molid=42731
42732	359.29254	[M+H]+	358.287	5	5β-Chol-11-en-24-oic Acid	C24H38O2			NO	metabo_info.php?molid=42732

42733	359.29254	[M+H]+	358.287	5	5β-Chol-14-en-24-oic Acid	C24H38O2		NO	metabo_info.php?molid=42733
42757	359.29254	[M+H]+	358.287	5	5β-Chol-2-en-24-oic Acid	C24H38O2		NO	metabo_info.php?molid=42757
42846	359.29254	[M+H]+	358.287	5	5β-Chol-3-en-24-oic Acid	C24H38O2		NO	metabo_info.php?molid=42846
42849	359.29254	[M+H]+	358.287	5	5β-Chol-6-en-24-oic Acid	C24H38O2		NO	metabo_info.php?molid=42849
42850	359.29254	[M+H]+	358.287	5	5β-Chol-7-en-24-oic Acid	C24H38O2		NO	metabo_info.php?molid=42850
42851	359.29254	[M+H]+	358.287	5	5β-Chol-8-en-24-oic Acid	C24H38O2		NO	metabo_info.php?molid=42851
42852	359.29254	[M+H]+	358.287	5	5β-Chol-8(14)-en-24-oic Acid	C24H38O2		NO	metabo_info.php?molid=42852
45897	359.29254	[M+H]+	358.287	5	12-[5]-ladderane-dodecanoic acid	C24H38O2		NO	metabo_info.php?molid=45897
53747	359.29254	[M+H]+	358.287	5	Hyrtial	C24H38O2		NO	metabo_info.php?molid=53747
74356	359.29254	[M+H]+	358.287	5	C24:5n-6,9,12,15,18	C24H38O2		NO	metabo_info.php?molid=74356
74357	359.29254	[M+H]+	358.287	5	24:5 (n-3); C24:5n-3,6,9,12,15	C24H38O2		NO	metabo_info.php?molid=74357
84456	359.29254	[M+H]+	358.287	5	5beta-Chol-9(11)-en-24-oic Acid	C24H38O2		NO	metabo_info.php?molid=84456
84458	359.29254	[M+H]+	358.287	5	5beta-Chol-14-en-24-oic Acid	C24H38O2		NO	metabo_info.php?molid=84458
84577	359.29254	[M+H]+	358.287	5	Chol-4-en-24-oic Acid	C24H38O2		NO	metabo_info.php?molid=84577
84578	359.29254	[M+H]+	358.287	5	Chol-5-en-24-oic Acid	C24H38O2		NO	metabo_info.php?molid=84578
84580	359.29254	[M+H]+	358.287	5	5beta-Chol-7-en-24-oic Acid	C24H38O2		NO	metabo_info.php?molid=84580
57793	359.29254	[M+H]+	358.287	5	Bufanolide skeleton	C24H38O2	C19660	NO	metabo_info.php?molid=57793

84581	359.29254	[M+H]+	358.287	5	5beta-Chol-8-en-24-oic Acid	C24H38O2			NO	metabo_info.php?molid=84581
84457	359.29254	[M+H]+	358.287	5	5beta-Chol-11-en-24-oic Acid	C24H38O2			NO	metabo_info.php?molid=84457
84582	359.29254	[M+H]+	358.287	5	5beta-Chol-8(14)-en-24-oic Acid	C24H38O2			NO	metabo_info.php?molid=84582
84484	359.29254	[M+H]+	358.287	5	5beta-Chol-2-en-24-oic Acid	C24H38O2			NO	metabo_info.php?molid=84484
90863	359.29254	[M+H]+	358.287	5	1-Phenyl-1,3-octadecanedione	C24H38O2			NO	metabo_info.php?molid=90863
84576	359.29254	[M+H]+	358.287	5	5beta-Chol-3-en-24-oic Acid	C24H38O2			NO	metabo_info.php?molid=84576
84579	359.29254	[M+H]+	358.287	5	5beta-Chol-6-en-24-oic Acid	C24H38O2			NO	metabo_info.php?molid=84579
34670	359.29254	[M+Na]+	336.303	1	7,7-dimethyl-5,8-Eicosadienoic Acid	C22H40O2	89560-01-0		YES	metabo_info.php?molid=34670
34806	359.29254	[M+Na]+	336.303	1	5,13-docosadienoic acid	C22H40O2			NO	metabo_info.php?molid=34806
34807	359.29254	[M+Na]+	336.303	1	13,16-docosadienoic acid	C22H40O2			NO	metabo_info.php?molid=34807
35069	359.29254	[M+Na]+	336.303	1	5Z,13Z-docosadienoic acid	C22H40O2			NO	metabo_info.php?molid=35069
35068	359.29254	[M+Na]+	336.303	1	13Z,16Z-docosadienoic acid	C22H40O2		C16533	NO	metabo_info.php?molid=35068
46398	359.29254	[M+Na]+	336.303	1	11Z,19-Eicosadienyl acetate	C22H40O2			NO	metabo_info.php?molid=46398
46400	359.29254	[M+Na]+	336.303	1	11Z,14Z-Eicosadienyl acetate	C22H40O2			NO	metabo_info.php?molid=46400
73689	359.29254	[M+Na]+	336.303	1	5,8-Eicosadienoic acid, 7,7-dimethyl-, [illegible]	C22H40O2			NO	metabo_info.php?molid=73689
73841	359.29254	[M+Na]+	336.303	1	5,13-docosadienoic acid	C22H40O2			NO	metabo_info.php?molid=73841
73842	359.29254	[M+Na]+	336.303	1	C22:2n-6,9	C22H40O2			NO	metabo_info.php?molid=73842

74072	359.29254	[M+Na]+	336.303	1	5Z,13Z-docosadienoic acid	C22H40O2	676-39-1		NO	metabo_info.php?molid=74072
74287	359.29254	[M+Na]+	336.303	1	13-Docosynoic acid	C22H40O2			NO	metabo_info.php?molid=74287
74397	359.29254	[M+Na]+	336.303	1	22:2(7Z,15Z)	C22H40O2			NO	metabo_info.php?molid=74397
75220	359.29254	[M+Na]+	336.303	1	22:2(7Z,13Z)	C22H40O2			NO	metabo_info.php?molid=75220
75224	359.29254	[M+Na]+	336.303	1	22:2(3Z,16Z)	C22H40O2			NO	metabo_info.php?molid=75224
97306	359.29254	[M+Na]+	336.303	1	(E)-3,7-Dimethyl-2,6-octadienyl dodecanoate	C22H40O2			NO	metabo_info.php?molid=97306
265060	359.29254	[M+Na]+	336.303	1	Butyl 9,12-octadecadienoate	C22H40O2			YES	metabo_info.php?molid=265060
90909	469.30562	[M+Na]+	446.318	4	3-Hydroxysintaxanthin	C31H42O2			NO	metabo_info.php?molid=90909
41897	469.30562	[M+Na]+	446.32	6	(24R)-24-fluoro-1α,25-dihydroxyvitamin D2 / (24R)-24-fluoro-1α,25-dihydroxyergocalciferol	C28H43FO3			NO	metabo_info.php?molid=41897
41898	469.30562	[M+Na]+	446.32	6	(24S)-24-fluoro-1α,25-dihydroxyvitamin D2 / (24S)-24-fluoro-1α,25-dihydroxyergocalciferol	C28H43FO3			NO	metabo_info.php?molid=41898
70539	244.08899	[M+H]+	243.081	0	4-Chloro-[2-(4-Pyridinyl)-1-butenyl]phenol	C15H14ClN		C15045	NO	metabo_info.php?molid=70539
64555	244.08899	[M+Na]+	221.101	5	1-Guanidino-1-deoxy-scyllo-inositol	C7H15N3O5		C04280	NO	metabo_info.php?molid=64555

1378	295.06363	[M+H]+	294.056	0	2-Butanone, 4-[6-(sulfooxy)-2-naphthalenyl]-	C14H14O5S	91488-18-5		NO	metabo_info.php?molid=1378
94193	175.03271	[M+H]+	174.025	1	Brassilexin	C9H6N2S	119752-76-0		NO	metabo_info.php?molid=94193
55	175.03271	[M+Na]+	152.044	3	Cystamine	C4H12N2S2	56-17-7		YES	metabo_info.php?molid=55
65437	498.34088	[M+H]+	497.335	3	Tumonoic Acid I	C27H47NO7			YES	metabo_info.php?molid=65437
2263	586.30337	[M+H]+	585.295	1	8\',10\'-Dihydroxydihydroergotamine	C33H39N5O5			NO	metabo_info.php?molid=2263
4040	586.30337	[M+Na]+	563.311	5	dihydroergocornine	C31H41N5O5			NO	metabo_info.php?molid=4040
90	245.07539	[M+H]+	244.07	5	Uridine	C9H12N2O6	58-96-8	C00299	YES	metabo_info.php?molid=90
5734	245.07539	[M+H]+	244.07	5	Pseudouridine	C9H12N2O6		C02067	NO	metabo_info.php?molid=5734
87	284.09627	[M+H]+	283.092	9	Guanosine	C10H13N5O5	118-00-3	C00387	YES	metabo_info.php?molid=87
66971	284.09627	[M+H]+	283.092	9	Isoguanosine	C10H13N5O5	1818-71-9	C08432	NO	metabo_info.php?molid=66971
44821	284.09627	[M+H]+	283.092	9	8-hydroxy-2\'-deoxy Guanosine	C10H13N5O5	NA		YES	metabo_info.php?molid=44821
72983	268.05964	[M+H]+	267.053	2	2-Methyl-1-nitroanthraquinone	C15H9NO4	129-15-7	C19264	NO	metabo_info.php?molid=72983
72847	268.05964	[M+Na]+	245.068	9	Proglinazine	C8H12ClN5O2	68228-20-6	C19105	NO	metabo_info.php?molid=72847
68502	268.05964	[M+Na]+	245.069	6	Haplopine	C13H11NO4	5876-17-5	C10694	NO	metabo_info.php?molid=68502
86851	497.33659	[M+Na]+	474.346	3	Dehydrocarpaine II	C28H46N2O4	72362-03-9		NO	metabo_info.php?molid=86851
40038	490.28639	[M+Na]+	467.301	8	PC(O-12:0/2:0)	C22H46NO7P			NO	metabo_info.php?molid=40038

40039	490.28639	[M+Na]+	467.301	8	PC(O-12:0/2:0)[U]	C22H46NO7P			NO	metabo_info.php?molid=40039
40278	490.28639	[M+Na]+	467.301	8	PC(14:0/0:0)	C22H46NO7P			YES	metabo_info.php?molid=40278
40279	490.28639	[M+Na]+	467.301	8	PC(14:0/0:0)[U]	C22H46NO7P			NO	metabo_info.php?molid=40279
40339	490.28639	[M+Na]+	467.301	8	PC(0:0/14:0)	C22H46NO7P			NO	metabo_info.php?molid=40339
61689	490.28639	[M+Na]+	467.301	8	LysoPC(14:0)	C22H46NO7P		C04230	NO	metabo_info.php?molid=61689
77696	490.28639	[M+Na]+	467.301	8	PE(17:0/0:0)	C22H46NO7P			YES	metabo_info.php?molid=77696
45554	207.0125	[M+H]+	206.006	5	Oxalomalic Acid	C6H6O8	89304-26-7	C01990	NO	metabo_info.php?molid=45554
63393	207.0125	[M+Na]+	184.023	0	5-Hydroxyisourate	C5H4N4O4		C11821	NO	metabo_info.php?molid=63393
43585	147.11174	[M+H]+	146.106	7	CARBACHOL	C6H14N2O2	51-83-2		YES	metabo_info.php?molid=43585
25	147.11174	[M+H]+	146.106	7	L-Lysine	C6H14N2O2	56-87-1	C00047	YES	metabo_info.php?molid=25
6919	147.11174	[M+H]+	146.106	7	D-Lysine	C6H14N2O2		C00739	YES	metabo_info.php?molid=6919
62789	147.11174	[M+H]+	146.106	7	(3S)-3,6-Diaminohexanoate	C6H14N2O2	4299-56-3	C01142	NO	metabo_info.php?molid=62789
62790	147.11174	[M+H]+	146.106	7	(3S,5S)-3,5-Diaminohexanoate	C6H14N2O2	17027-83-7	C01186	NO	metabo_info.php?molid=62790
63459	147.11174	[M+H]+	146.106	7	2,5-Diaminohexanoate	C6H14N2O2		C05161	NO	metabo_info.php?molid=63459
71200	147.11174	[M+H]+	146.106	7	Lysine	C6H14N2O2		C16440	NO	metabo_info.php?molid=71200
4041	612.31865	[M+H]+	611.311	0	dihydroergocristine	C35H41N5O5			NO	metabo_info.php?molid=4041
34742	255.23035	[M+H]+	254.225	5	Gaidic acid	C16H30O2			NO	metabo_info.php?molid=34742

34743	255.23035	[M+H]+	254.225	5	7-palmitoleic acid	C16H30O2			NO	metabo_info.php?molid=34743
34745	255.23035	[M+H]+	254.225	5	cis-10-palmitoleic acid	C16H30O2			NO	metabo_info.php?molid=34745
34924	255.23035	[M+H]+	254.225	5	10-hexadecenoic acid	C16H30O2			NO	metabo_info.php?molid=34924
34925	255.23035	[M+H]+	254.225	5	11-hexadecenoic acid	C16H30O2			NO	metabo_info.php?molid=34925
34926	255.23035	[M+H]+	254.225	5	11Z-hexadecenoic acid	C16H30O2			YES	metabo_info.php?molid=34926
34927	255.23035	[M+H]+	254.225	5	13-hexadecenoic acid	C16H30O2			NO	metabo_info.php?molid=34927
34928	255.23035	[M+H]+	254.225	5	13Z-hexadecenoic acid	C16H30O2			NO	metabo_info.php?molid=34928
34930	255.23035	[M+H]+	254.225	5	3E-hexadecenoic acid	C16H30O2			NO	metabo_info.php?molid=34930
34931	255.23035	[M+H]+	254.225	5	6Z-hexadecenoic acid	C16H30O2			NO	metabo_info.php?molid=34931
45058	255.23035	[M+H]+	254.225	5	cis-7-Hexadecenoic Acid	C16H30O2	2416-19-5		YES	metabo_info.php?molid=45058
188	255.23035	[M+H]+	254.225	5	cis-9-palmitoleic acid	C16H30O2	373-49-9	C08362	YES	metabo_info.php?molid=188
34744	255.23035	[M+H]+	254.225	5	trans-9-palmitoleic acid	C16H30O2		C08362	NO	metabo_info.php?molid=34744
34929	255.23035	[M+H]+	254.225	5	C16:1n-14	C16H30O2			NO	metabo_info.php?molid=34929
45810	255.23035	[M+H]+	254.225	5	Hypogeic acid	C16H30O2			NO	metabo_info.php?molid=45810
46282	255.23035	[M+H]+	254.225	5	10E-Tetradecenyl acetate	C16H30O2			NO	metabo_info.php?molid=46282
46284	255.23035	[M+H]+	254.225	5	11E-Tetradecenyl acetate	C16H30O2	NA		NO	metabo_info.php?molid=46284
46285	255.23035	[M+H]+	254.225	5	12E-Tetradecenyl acetate	C16H30O2			NO	metabo_info.php?molid=46285

46286	255.23035	[M+H]+	254.225	5	3E-Tetradecenyl acetate	C16H30O2	NO	metabo_info.php?molid=46286
46287	255.23035	[M+H]+	254.225	5	5E-Tetradecenyl acetate	C16H30O2	NO	metabo_info.php?molid=46287
46288	255.23035	[M+H]+	254.225	5	6E-Tetradecenyl acetate	C16H30O2	NO	metabo_info.php?molid=46288
46289	255.23035	[M+H]+	254.225	5	7E-Tetradecenyl acetate	C16H30O2	NO	metabo_info.php?molid=46289
46290	255.23035	[M+H]+	254.225	5	8E-Tetradecenyl acetate	C16H30O2	NO	metabo_info.php?molid=46290
46292	255.23035	[M+H]+	254.225	5	9E-Tetradecenyl acetate	C16H30O2	NO	metabo_info.php?molid=46292
46306	255.23035	[M+H]+	254.225	5	10Z-Tetradecenyl acetate	C16H30O2	NO	metabo_info.php?molid=46306
46308	255.23035	[M+H]+	254.225	5	11Z-Tetradecenyl acetate	C16H30O2	NO	metabo_info.php?molid=46308
46309	255.23035	[M+H]+	254.225	5	12Z-Tetradecenyl acetate	C16H30O2	NO	metabo_info.php?molid=46309
46310	255.23035	[M+H]+	254.225	5	3Z-Tetradecenyl acetate	C16H30O2	NO	metabo_info.php?molid=46310
46311	255.23035	[M+H]+	254.225	5	5Z-Tetradecenyl acetate	C16H30O2	NO	metabo_info.php?molid=46311
46312	255.23035	[M+H]+	254.225	5	6Z-Tetradecenyl acetate	C16H30O2	NO	metabo_info.php?molid=46312
46313	255.23035	[M+H]+	254.225	5	7Z-Tetradecenyl acetate	C16H30O2	NO	metabo_info.php?molid=46313
46314	255.23035	[M+H]+	254.225	5	8Z-Tetradecenyl acetate	C16H30O2	NO	metabo_info.php?molid=46314
46315	255.23035	[M+H]+	254.225	5	9Z-Tetradecenyl acetate	C16H30O2	NO	metabo_info.php?molid=46315
73589	255.23035	[M+H]+	254.225	5	2,4-dimethyl-2E-tetradecenoic acid	C16H30O2	NO	metabo_info.php?molid=73589
73595	255.23035	[M+H]+	254.225	5	14-methyl-4-pentadecenoic acid	C16H30O2	NO	metabo_info.php?molid=73595

73596	255.23035	[M+H]+	254.225	5	2-hexyl-2-decenoic acid	C16H30O2		NO	metabo_info.php?molid=73596
73597	255.23035	[M+H]+	254.225	5	6-isopentyl-9-methyl-5-decenoic acid	C16H30O2		NO	metabo_info.php?molid=73597
73935	255.23035	[M+H]+	254.225	5	C16:1n-6	C16H30O2		NO	metabo_info.php?molid=73935
73936	255.23035	[M+H]+	254.225	5	C16:1n-5	C16H30O2		NO	metabo_info.php?molid=73936
73937	255.23035	[M+H]+	254.225	5	cis-Palmitvaccenic acid	C16H30O2		NO	metabo_info.php?molid=73937
73938	255.23035	[M+H]+	254.225	5	13-hexadecenoic acid	C16H30O2		NO	metabo_info.php?molid=73938
73939	255.23035	[M+H]+	254.225	5	13Z-hexadecenoic acid	C16H30O2		NO	metabo_info.php?molid=73939
73941	255.23035	[M+H]+	254.225	5	3E-Hexadecenoic acid	C16H30O2	2457-70-7	NO	metabo_info.php?molid=73941
73942	255.23035	[M+H]+	254.225	5	Sapienic acid	C16H30O2		NO	metabo_info.php?molid=73942
74346	255.23035	[M+H]+	254.225	5	hexadec-7Z-enoic acid	C16H30O2		NO	metabo_info.php?molid=74346
74390	255.23035	[M+H]+	254.225	5	16:1(5Z)	C16H30O2		NO	metabo_info.php?molid=74390
74429	255.23035	[M+H]+	254.225	5	15:1(4)(13Me)	C16H30O2		NO	metabo_info.php?molid=74429
74430	255.23035	[M+H]+	254.225	5	16:1(4)	C16H30O2		NO	metabo_info.php?molid=74430
87443	255.23035	[M+H]+	254.225	5	5-Dodecyldihydro-2(3H)-furanone	C16H30O2	730-46-1	NO	metabo_info.php?molid=87443
87885	255.23035	[M+H]+	254.225	5	15-Hexadecanolide	C16H30O2	69297-56-9	NO	metabo_info.php?molid=87885
88581	255.23035	[M+H]+	254.225	5	(Z)-5-Hexadecenoic acid	C16H30O2	7056-90-8	NO	metabo_info.php?molid=88581
89535	255.23035	[M+H]+	254.225	5	(E)-3-Hexadecenoic acid	C16H30O2	1686-10-8	NO	metabo_info.php?molid=89535

93569	255.23035	[M+H]+	254.225	5	Citronellyl hexanoate	C16H30O2	10580-25-3		NO	metabo_info.php?molid=93569
95819	255.23035	[M+H]+	254.225	5	(Z)-14-Methyl-6-pentadecenoic acid	C16H30O2	123739-73-1		NO	metabo_info.php?molid=95819
96484	255.23035	[M+H]+	254.225	5	Δ2-trans-Hexadecenoic Acid	C16H30O2	929-79-3		YES	metabo_info.php?molid=96484
96485	255.23035	[M+H]+	254.225	5	Δ2-cis-Hexadecenoic Acid	C16H30O2	2825-68-5		YES	metabo_info.php?molid=96485
97213	255.23035	[M+H]+	254.225	5	ethyl 7E-tetradecenoate	C16H30O2			NO	metabo_info.php?molid=97213
97214	255.23035	[M+H]+	254.225	5	ethyl 9E-tetradecenoate	C16H30O2			NO	metabo_info.php?molid=97214
97215	255.23035	[M+H]+	254.225	5	ethyl 9Z-tetradecenoate	C16H30O2			NO	metabo_info.php?molid=97215
97225	255.23035	[M+H]+	254.225	5	dodecyl 2E-butenoate	C16H30O2			NO	metabo_info.php?molid=97225
97243	255.23035	[M+H]+	254.225	5	(Z)-7-Dodecenyl butyrate	C16H30O2			NO	metabo_info.php?molid=97243
97389	255.23035	[M+H]+	254.225	5	Vittatalactone	C16H30O2			NO	metabo_info.php?molid=97389
97405	255.23035	[M+H]+	254.225	5	15R-Hexadecanolide	C16H30O2			NO	metabo_info.php?molid=97405
97407	255.23035	[M+H]+	254.225	5	16-Hexadecanolide	C16H30O2			NO	metabo_info.php?molid=97407
97408	255.23035	[M+H]+	254.225	5	delta-hexadecalactone	C16H30O2			NO	metabo_info.php?molid=97408
61172	861.54391	[M+H]+	860.541	5	PI(16:0/20:3(5Z,8Z,11Z))	C45H81O13P		C00626	NO	metabo_info.php?molid=61172
61173	861.54391	[M+H]+	860.541	5	PI(16:0/20:3(8Z,11Z,14Z))	C45H81O13P		C00626	NO	metabo_info.php?molid=61173
61195	861.54391	[M+H]+	860.541	5	PI(18:0/18:3(6Z,9Z,12Z))	C45H81O13P		C00626	NO	metabo_info.php?molid=61195
61196	861.54391	[M+H]+	860.541	5	PI(18:0/18:3(9Z,12Z,15Z))	C45H81O13P		C00626	NO	metabo_info.php?molid=61196

61211	861.54391	[M+H]+	860.541	5	PI(18:1(11Z)/18:2(9Z,12Z))	C45H81O13P	C00626	NO	metabo_info.php?molid=61211
61223	861.54391	[M+H]+	860.541	5	PI(18:1(9Z)/18:2(9Z,12Z))	C45H81O13P	C00626	NO	metabo_info.php?molid=61223
61233	861.54391	[M+H]+	860.541	5	PI(18:2(9Z,12Z)/18:1(11Z))	C45H81O13P	C00626	NO	metabo_info.php?molid=61233
61234	861.54391	[M+H]+	860.541	5	PI(18:2(9Z,12Z)/18:1(9Z))	C45H81O13P	C00626	NO	metabo_info.php?molid=61234
61243	861.54391	[M+H]+	860.541	5	PI(18:3(6Z,9Z,12Z)/18:0)	C45H81O13P	C00626	NO	metabo_info.php?molid=61243
61247	861.54391	[M+H]+	860.541	5	PI(18:3(9Z,12Z,15Z)/18:0)	C45H81O13P	C00626	NO	metabo_info.php?molid=61247
61264	861.54391	[M+H]+	860.541	5	PI(20:3(5Z,8Z,11Z)/16:0)	C45H81O13P	C00626	NO	metabo_info.php?molid=61264
61271	861.54391	[M+H]+	860.541	5	PI(20:3(8Z,11Z,14Z)/16:0)	C45H81O13P	C00626	NO	metabo_info.php?molid=61271
80126	861.54391	[M+H]+	860.541	5	PI(14:1(9Z)/22:2(13Z,16Z))	C45H81O13P		NO	metabo_info.php?molid=80126
80208	861.54391	[M+H]+	860.541	5	PI(16:1(9Z)/20:2(11Z,14Z))	C45H81O13P		NO	metabo_info.php?molid=80208
80285	861.54391	[M+H]+	860.541	5	PI(17:2(9Z,12Z)/19:1(9Z))	C45H81O13P		NO	metabo_info.php?molid=80285
80480	861.54391	[M+H]+	860.541	5	PI(19:1(9Z)/17:2(9Z,12Z))	C45H81O13P		NO	metabo_info.php?molid=80480
80555	861.54391	[M+H]+	860.541	5	PI(20:2(11Z,14Z)/16:1(9Z))	C45H81O13P		NO	metabo_info.php?molid=80555
80750	861.54391	[M+H]+	860.541	5	PI(22:2(13Z,16Z)/14:1(9Z))	C45H81O13P		NO	metabo_info.php?molid=80750
61166	861.54391	[M+Na]+	838.557	2	PI(16:0/18:0)	C43H83O13P	C00626	NO	metabo_info.php?molid=61166
61190	861.54391	[M+Na]+	838.557	2	PI(18:0/16:0)	C43H83O13P	C00626	NO	metabo_info.php?molid=61190
80841	861.54391	[M+Na]+	838.557	2	PI(22:0/12:0)	C43H83O13P		NO	metabo_info.php?molid=80841

80845	861.54391	[M+Na]+	838.557	2	PI(21:0/13:0)	C43H83O13P			NO	metabo_info.php?molid=80845
80856	861.54391	[M+Na]+	838.557	2	PI(20:0/14:0)	C43H83O13P			NO	metabo_info.php?molid=80856
80861	861.54391	[M+Na]+	838.557	2	PI(19:0/15:0)	C43H83O13P			NO	metabo_info.php?molid=80861
80888	861.54391	[M+Na]+	838.557	2	PI(17:0/17:0)	C43H83O13P			NO	metabo_info.php?molid=80888
80906	861.54391	[M+Na]+	838.557	2	PI(15:0/19:0)	C43H83O13P			NO	metabo_info.php?molid=80906
80918	861.54391	[M+Na]+	838.557	2	PI(14:0/20:0)	C43H83O13P			NO	metabo_info.php?molid=80918
80926	861.54391	[M+Na]+	838.557	2	PI(13:0/21:0)	C43H83O13P			NO	metabo_info.php?molid=80926
80932	861.54391	[M+Na]+	838.557	2	PI(12:0/22:0)	C43H83O13P			NO	metabo_info.php?molid=80932
86851	497.33868	[M+Na]+	474.346	7	Dehydrocarpaine II	C28H46N2O4	72362-03-9		NO	metabo_info.php?molid=86851
182	496.33507	[M+H]+	495.332	9	PC(16:0/0:0)[U] / PC(16:0/0:0)[rac]	C24H50NO7P	17364-16-8	C04102	YES	metabo_info.php?molid=182
40048	496.33507	[M+H]+	495.332	9	PC(O-14:0/2:0)	C24H50NO7P			NO	metabo_info.php?molid=40048
40049	496.33507	[M+H]+	495.332	9	PC(O-14:0/2:0)[U]	C24H50NO7P			NO	metabo_info.php?molid=40049
40284	496.33507	[M+H]+	495.332	9	PC(16:0/0:0)	C24H50NO7P			NO	metabo_info.php?molid=40284
40285	496.33507	[M+H]+	495.332	9	PC(16:0/0:0)[S]	C24H50NO7P			NO	metabo_info.php?molid=40285
40286	496.33507	[M+H]+	495.332	9	PC(16:0/0:0)[U]	C24H50NO7P			NO	metabo_info.php?molid=40286
40340	496.33507	[M+H]+	495.332	9	PC(0:0/16:0)	C24H50NO7P			NO	metabo_info.php?molid=40340
40341	496.33507	[M+H]+	495.332	9	PC(0:0/16:0)[U]	C24H50NO7P			NO	metabo_info.php?molid=40341

61692	496.33507	[M+H]+	495.332	9	LysoPC(16:0)	C24H50NO7P		C04230	NO	metabo_info.php?molid=61692
77694	496.33507	[M+H]+	495.332	9	PE(19:0/0:0)	C24H50NO7P			NO	metabo_info.php?molid=77694
102768	496.33507	[M+H]+	495.332	9	PC(16:0/0:0)[rac]	C24H50NO7P			NO	metabo_info.php?molid=102768
58392	496.33507	[M+Na]+	473.351	9	Docosa-4,7,10,13,16-pentaenoyl carnitine	C29H47NO4			NO	metabo_info.php?molid=58392
58428	496.33507	[M+Na]+	473.351	9	Clupanodonyl carnitine	C29H47NO4			NO	metabo_info.php?molid=58428
87655	496.33507	[M+Na]+	473.351	9	23-Acetoxysoladulcidine	C29H47NO4	152128-85-3		NO	metabo_info.php?molid=87655
43413	524.36604	[M+H]+	523.364	9	enantio-PAF C-16	C26H54NO7P	117985-57-6		YES	metabo_info.php?molid=43413
34488	524.36604	[M+H]+	523.364	9	PAF C-16	C26H54NO7P	74389-68-7		YES	metabo_info.php?molid=34488
40161	524.36604	[M+H]+	523.364	9	PC(2:0/O-16:0)[U]	C26H54NO7P			NO	metabo_info.php?molid=40161
40075	524.36604	[M+H]+	523.364	9	PC(O-16:0/2:0)	C26H54NO7P			NO	metabo_info.php?molid=40075
40076	524.36604	[M+H]+	523.364	9	PC(O-16:0/2:0)[S]	C26H54NO7P			NO	metabo_info.php?molid=40076
40077	524.36604	[M+H]+	523.364	9	PC(O-16:0/2:0)[U]	C26H54NO7P			NO	metabo_info.php?molid=40077
40292	524.36604	[M+H]+	523.364	9	PC(18:0/0:0)	C26H54NO7P			NO	metabo_info.php?molid=40292
40293	524.36604	[M+H]+	523.364	9	PC(18:0/0:0)[S]	C26H54NO7P			NO	metabo_info.php?molid=40293
40294	524.36604	[M+H]+	523.364	9	PC(18:0/0:0)[U]	C26H54NO7P			NO	metabo_info.php?molid=40294
40342	524.36604	[M+H]+	523.364	9	PC(0:0/18:0)	C26H54NO7P			NO	metabo_info.php?molid=40342
40343	524.36604	[M+H]+	523.364	9	PC(0:0/18:0)[S]	C26H54NO7P			NO	metabo_info.php?molid=40343

40344	524.36604	[M+H]+	523.364	9	PC(0:0/18:0)[U]	C26H54NO7P			NO	metabo_info.php?molid=40344
61694	524.36604	[M+H]+	523.364	9	LysoPC(18:0)	C26H54NO7P		C04230	NO	metabo_info.php?molid=61694
61991	524.36604	[M+H]+	523.364	9	LysoPC(0:0/18:0)	C26H54NO7P			NO	metabo_info.php?molid=61991
77692	524.36604	[M+H]+	523.364	9	PE(21:0/0:0)	C26H54NO7P			NO	metabo_info.php?molid=77692
35473	159.02646	[M+Na]+	136.037	0	D-threonic acid	C4H8O5			NO	metabo_info.php?molid=35473
35474	159.02646	[M+Na]+	136.037	0	DL-erythronic acid	C4H8O5			NO	metabo_info.php?molid=35474
4244	159.02646	[M+Na]+	136.037	0	Threonate	C4H8O5	70753-61-6	C01620	YES	metabo_info.php?molid=4244
45855	159.02646	[M+Na]+	136.037	0	Erythronic acid	C4H8O5			NO	metabo_info.php?molid=45855
45859	159.02646	[M+Na]+	136.037	0	Threonic acid	C4H8O5			NO	metabo_info.php?molid=45859
87453	159.02646	[M+Na]+	136.038	5	1-Pentanesulfenothioic acid	C5H12S2	86849-52-7		NO	metabo_info.php?molid=87453
88945	159.02646	[M+Na]+	136.038	5	Ethyl propyl disulfide	C5H12S2	30453-31-7		NO	metabo_info.php?molid=88945
88946	159.02646	[M+Na]+	136.038	5	Ethyl isopropyl disulfide	C5H12S2	53966-36-2		NO	metabo_info.php?molid=88946
83	159.02646	[M+Na]+	136.039	8	Hypoxanthine	C5H4N4O	68-94-0	C00262	YES	metabo_info.php?molid=83
865	159.02646	[M+Na]+	136.039	8	allopurinol	C5H4N4O	315-30-0	C06816	YES	metabo_info.php?molid=865
43413	546.34753	[M+Na]+	523.364	9	enantio-PAF C-16	C26H54NO7P	117985-57-6		YES	metabo_info.php?molid=43413
34488	546.34753	[M+Na]+	523.364	9	PAF C-16	C26H54NO7P	74389-68-7		YES	metabo_info.php?molid=34488
40161	546.34753	[M+Na]+	523.364	9	PC(2:0/O-16:0)[U]	C26H54NO7P			NO	metabo_info.php?molid=40161

67424	671.27794	[M+Na]+	648.293	7	Gnididin	C37H44O10	55306-11-1	C09096	NO	metabo_info.php?molid=67424
94030	306.89208	[M+H]+	305.883	6	Di-2-propenyl heptasulfide	C6H10S7	139693-24-6		NO	metabo_info.php?molid=94030
96809	306.89208	[M+Na]+	283.905	6	3,3-Dibromo-2-n-butylacrylic acid	C7H10Br2O2			NO	metabo_info.php?molid=96809
58177	162.90578	[M+H]+	161.899	0	Phosphoroselenoic acid	H3O3PSe	25758-66-1	C05172	NO	metabo_info.php?molid=58177
92957	739.26513	[M+Na]+	716.269	8	Bn-NCC-1	C37H40N4O11	152571-56-7		NO	metabo_info.php?molid=92957
182	518.31679	[M+Na]+	495.332	9	PC(16:0/0:0)[U] / PC(16:0/0:0)[rac]	C24H50NO7P	17364-16-8	C04102	YES	metabo_info.php?molid=182
40048	518.31679	[M+Na]+	495.332	9	PC(O-14:0/2:0)	C24H50NO7P			NO	metabo_info.php?molid=40048
40049	518.31679	[M+Na]+	495.332	9	PC(O-14:0/2:0)[U]	C24H50NO7P			NO	metabo_info.php?molid=40049
40284	518.31679	[M+Na]+	495.332	9	PC(16:0/0:0)	C24H50NO7P			NO	metabo_info.php?molid=40284
40285	518.31679	[M+Na]+	495.332	9	PC(16:0/0:0)[S]	C24H50NO7P			NO	metabo_info.php?molid=40285
40286	518.31679	[M+Na]+	495.332	9	PC(16:0/0:0)[U]	C24H50NO7P			NO	metabo_info.php?molid=40286
40340	518.31679	[M+Na]+	495.332	9	PC(0:0/16:0)	C24H50NO7P			NO	metabo_info.php?molid=40340
40341	518.31679	[M+Na]+	495.332	9	PC(0:0/16:0)[U]	C24H50NO7P			NO	metabo_info.php?molid=40341
61692	518.31679	[M+Na]+	495.332	9	LysoPC(16:0)	C24H50NO7P		C04230	NO	metabo_info.php?molid=61692
77694	518.31679	[M+Na]+	495.332	9	PE(19:0/0:0)	C24H50NO7P			NO	metabo_info.php?molid=77694
102768	518.31679	[M+Na]+	495.332	9	PC(16:0/0:0)[rac]	C24H50NO7P			NO	metabo_info.php?molid=102768
40008	522.35048	[M+H]+	521.348	9	PC(O-16:1(11Z)/2:0)	C26H52NO7P			NO	metabo_info.php?molid=40008

40009	522.35048	[M+H]+	521.348	9	PC(O-16:1(9E)/2:0)[U]	C26H52NO7P			NO	metabo_info.php?molid=40009
40010	522.35048	[M+H]+	521.348	9	PC(O-16:1(9Z)/2:0)[U]	C26H52NO7P			NO	metabo_info.php?molid=40010
40174	522.35048	[M+H]+	521.348	9	PC(P-16:0/2:0)	C26H52NO7P			NO	metabo_info.php?molid=40174
40295	522.35048	[M+H]+	521.348	9	PC(18:1(6Z)/0:0)	C26H52NO7P			NO	metabo_info.php?molid=40295
40296	522.35048	[M+H]+	521.348	9	PC(18:1(9E)/0:0)	C26H52NO7P			NO	metabo_info.php?molid=40296
40297	522.35048	[M+H]+	521.348	9	PC(18:1(9)/0:0)[U]	C26H52NO7P			NO	metabo_info.php?molid=40297
40298	522.35048	[M+H]+	521.348	9	PC(18:1(9Z)/0:0)	C26H52NO7P			NO	metabo_info.php?molid=40298
40299	522.35048	[M+H]+	521.348	9	PC(18:1(9Z)/0:0)[U]	C26H52NO7P		C03916	NO	metabo_info.php?molid=40299
40345	522.35048	[M+H]+	521.348	9	PC(0:0/18:1(6Z))	C26H52NO7P			NO	metabo_info.php?molid=40345
40346	522.35048	[M+H]+	521.348	9	PC(0:0/18:1(9E))	C26H52NO7P			NO	metabo_info.php?molid=40346
40347	522.35048	[M+H]+	521.348	9	PC(0:0/18:1(9E))[U]	C26H52NO7P			NO	metabo_info.php?molid=40347
40348	522.35048	[M+H]+	521.348	9	PC(0:0/18:1(9Z))	C26H52NO7P			NO	metabo_info.php?molid=40348
40349	522.35048	[M+H]+	521.348	9	PC(0:0/18:1(9Z))[U]	C26H52NO7P			NO	metabo_info.php?molid=40349
46689	522.35048	[M+H]+	521.348	9	PC(18:1(9E)/0:0)[U]	C26H52NO7P			NO	metabo_info.php?molid=46689
61695	522.35048	[M+H]+	521.348	9	LysoPC(18:1(11Z))	C26H52NO7P		C04230	NO	metabo_info.php?molid=61695
102769	522.35048	[M+H]+	521.348	9	PC(18:1(9Z)/0:0)[rac]	C26H52NO7P			NO	metabo_info.php?molid=102769
184	522.35048	[M+H]+	521.348	9	PC(18:1(9Z)/0:0)[U] / PC(18:1(9Z)/0:0)[rac]	C26H52NO7P	3542-29-8		NO	metabo_info.php?molid=184

63723	654.28833	[M+H]+	653.284	3	Rifamycin W-hemiacetal	C35H43NO11		C14722	NO	metabo_info.php?molid=63723
89966	654.28833	[M+Na]+	631.297	3	Gliadin	C29H41N7O9			NO	metabo_info.php?molid=89966
67132	654.28833	[M+Na]+	631.299	0	Mesaconitine	C33H45NO11	2752-64-9	C08698	NO	metabo_info.php?molid=67132
71023	523.35295	[M+Na]+	500.361	4	Vicenistatin	C30H48N2O4	150999-05-6	C15688	NO	metabo_info.php?molid=71023
67520	519.31994	[M+H]+	518.316	6	Hodgkinsine	C33H38N6	18210-71-4	C09211	NO	metabo_info.php?molid=67520
95551	291.06739	[M+H]+	290.058	7	2-(4-Hydroxyphenyl)naphthalic anhydride	C18H10O4	158922-28-2		NO	metabo_info.php?molid=95551
63171	291.06739	[M+Na]+	268.079	4	2(α-D-Mannosyl)-D-glycerate	C9H16O9		C11544	NO	metabo_info.php?molid=63171
5414	291.06739	[M+Na]+	268.079	4	3-Deoxy-D-glycero-D-galacto-2-nonulosonic acid	C9H16O9			NO	metabo_info.php?molid=5414
84	291.06739	[M+Na]+	268.081	8	Inosine	C10H12N4O5	58-63-9	C00294	YES	metabo_info.php?molid=84
867	291.06739	[M+Na]+	268.081	8	Allopurinol-1-ribonucleoside	C10H12N4O5	16220-07-8		NO	metabo_info.php?molid=867
3022	291.06739	[M+Na]+	268.081	8	Arabinosylhypoxanthine	C10H12N4O5	7013-16-3		NO	metabo_info.php?molid=3022
40008	544.33208	[M+Na]+	521.348	9	PC(O-16:1(11Z)/2:0)	C26H52NO7P			NO	metabo_info.php?molid=40008
40009	544.33208	[M+Na]+	521.348	9	PC(O-16:1(9E)/2:0)[U]	C26H52NO7P			NO	metabo_info.php?molid=40009
40010	544.33208	[M+Na]+	521.348	9	PC(O-16:1(9Z)/2:0)[U]	C26H52NO7P			NO	metabo_info.php?molid=40010
40174	544.33208	[M+Na]+	521.348	9	PC(P-16:0/2:0)	C26H52NO7P			NO	metabo_info.php?molid=40174
40295	544.33208	[M+Na]+	521.348	9	PC(18:1(6Z)/0:0)	C26H52NO7P			NO	metabo_info.php?molid=40295

40296	544.33208	[M+Na]+	521.348	9	PC(18:1(9E)/0:0)	C26H52NO7P			NO	metabo_info.php?molid=40296
40297	544.33208	[M+Na]+	521.348	9	PC(18:1(9)/0:0)[U]	C26H52NO7P			NO	metabo_info.php?molid=40297
40298	544.33208	[M+Na]+	521.348	9	PC(18:1(9Z)/0:0)	C26H52NO7P			NO	metabo_info.php?molid=40298
40299	544.33208	[M+Na]+	521.348	9	PC(18:1(9Z)/0:0)[U]	C26H52NO7P		C03916	NO	metabo_info.php?molid=40299
40345	544.33208	[M+Na]+	521.348	9	PC(0:0/18:1(6Z))	C26H52NO7P			NO	metabo_info.php?molid=40345
40346	544.33208	[M+Na]+	521.348	9	PC(0:0/18:1(9E))	C26H52NO7P			NO	metabo_info.php?molid=40346
40347	544.33208	[M+Na]+	521.348	9	PC(0:0/18:1(9E))[U]	C26H52NO7P			NO	metabo_info.php?molid=40347
40348	544.33208	[M+Na]+	521.348	9	PC(0:0/18:1(9Z))	C26H52NO7P			NO	metabo_info.php?molid=40348
40349	544.33208	[M+Na]+	521.348	9	PC(0:0/18:1(9Z))[U]	C26H52NO7P			NO	metabo_info.php?molid=40349
46689	544.33208	[M+Na]+	521.348	9	PC(18:1(9E)/0:0)[U]	C26H52NO7P			NO	metabo_info.php?molid=46689
61695	544.33208	[M+Na]+	521.348	9	LysoPC(18:1(11Z))	C26H52NO7P		C04230	NO	metabo_info.php?molid=61695
102769	544.33208	[M+Na]+	521.348	9	PC(18:1(9Z)/0:0)[rac]	C26H52NO7P			NO	metabo_info.php?molid=102769
184	544.33208	[M+Na]+	521.348	9	PC(18:1(9Z)/0:0)[U] / PC(18:1(9Z)/0:0)[rac]	C26H52NO7P	3542-29-8		NO	metabo_info.php?molid=184
72426	722.27494	[M+Na]+	699.289	4	Aclacinomycin S	C36H45NO13		C18635	NO	metabo_info.php?molid=72426
68100	261.04176	[M+H]+	260.032	9	Demethylbellidifolin	C13H8O6	2980-32-7	C10056	NO	metabo_info.php?molid=68100
68120	261.04176	[M+H]+	260.032	9	Norathyriol	C13H8O6	3542-72-1	C10086	NO	metabo_info.php?molid=68120
68122	261.04176	[M+H]+	260.032	9	Norswertianin	C13H8O6	22172-15-2	C10088	NO	metabo_info.php?molid=68122

86098	261.04176	[M+H]+	260.032	9	Urolithin D	C13H8O6			NO	metabo_info.php?molid=86098
72338	261.04176	[M+Na]+	238.051	6	Ethychlozate	C11H11ClN2O2	27512-72-7	C18532	NO	metabo_info.php?molid=72338
73297	261.04176	[M+Na]+	238.051	6	7-Chloro-L-tryptophan	C11H11ClN2O2		C19687	NO	metabo_info.php?molid=73297
86946	261.04176	[M+Na]+	238.051	6	L-4-Chlorotryptophan	C11H11ClN2O2	52448-14-3		NO	metabo_info.php?molid=86946
89367	261.04176	[M+Na]+	238.051	6	(±)-6-Chlorotryptophan	C11H11ClN2O2	17808-21-8		NO	metabo_info.php?molid=89367
88965	261.04176	[M+Na]+	238.052	2	1-Propenyl 1-(propylsulfinyl)propyl disulfide	C9H18OS3			NO	metabo_info.php?molid=88965
88966	261.04176	[M+Na]+	238.052	2	1-(1-Propenylsulfinyl)propyl propyl disulfide	C9H18OS3			NO	metabo_info.php?molid=88966
91513	261.04176	[M+Na]+	238.052	2	1-(2-Propenylsulfinyl)propyl propyl disulfide	C9H18OS3			NO	metabo_info.php?molid=91513
91518	261.04176	[M+Na]+	238.052	2	2-Propenyl 1-(propylsulfinyl)propyl disulfide	C9H18OS3			NO	metabo_info.php?molid=91518
92150	545.33328	[M+Na]+	522.34	6	Polysorbate 20	C26H50O10	9005-64-5		NO	metabo_info.php?molid=92150
78853	568.3563	[M+H]+	567.354	8	PS(21:0/0:0)	C27H54NO9P			NO	metabo_info.php?molid=78853
70908	287.06714	[M+H]+	286.061	2	2-Methacryloyloxyethyl phenyl phosphate	C12H15O6P		C15439	NO	metabo_info.php?molid=70908
69592	287.06714	[M+H]+	286.062	7	Olprinone hydrochloride	C14H11ClN4O	119615-63-3	C13546	NO	metabo_info.php?molid=69592
2205	287.06714	[M+Na]+	264.078	0	Pyrimethamine-3-N-Oxide	C12H13ClN4O	91284-15-0		NO	metabo_info.php?molid=2205
2206	287.06714	[M+Na]+	264.078	0	Pyrimethamine-1-N-Oxide	C12H13ClN4O	150034-10-9		NO	metabo_info.php?molid=2206

85673	287.06714	[M+Na]+	264.078	0	Aspartyl-Methionine	C9H16N2O5S			NO	metabo_info.php?molid=85673
85871	287.06714	[M+Na]+	264.078	0	Methionyl-Aspartate	C9H16N2O5S			NO	metabo_info.php?molid=85871
88096	287.06714	[M+Na]+	264.078	0	gamma-Glutamyl-S-methylcysteine	C9H16N2O5S			NO	metabo_info.php?molid=88096
6656	287.06714	[M+Na]+	264.078	0	N-Acetylcystathionine	C9H16N2O5S	20619-80-1		NO	metabo_info.php?molid=6656
51808	287.06714	[M+Na]+	264.079	2	2\'-Hydroxyfurano[2\'\',3\'\':4\',3\']chalcone	C17H12O3			NO	metabo_info.php?molid=51808
46149	237.21976	[M+H]+	236.214	6	4E,6E,10Z-Hexadecatrien-1-ol	C16H28O			NO	metabo_info.php?molid=46149
46152	237.21976	[M+H]+	236.214	6	4E,6Z,10Z-Hexadecatrien-1-ol	C16H28O			NO	metabo_info.php?molid=46152
46153	237.21976	[M+H]+	236.214	6	13Z-Hexadecen-11-yn-1-ol	C16H28O			NO	metabo_info.php?molid=46153
46485	237.21976	[M+H]+	236.214	6	9E,11E-Hexadecadienal	C16H28O			NO	metabo_info.php?molid=46485
46490	237.21976	[M+H]+	236.214	6	10E,12Z-Hexadecadienal	C16H28O		C20128	NO	metabo_info.php?molid=46490
46491	237.21976	[M+H]+	236.214	6	11E,13Z-Hexadecadienal	C16H28O			NO	metabo_info.php?molid=46491
46492	237.21976	[M+H]+	236.214	6	4E,6Z-Hexadecadienal	C16H28O			NO	metabo_info.php?molid=46492
46493	237.21976	[M+H]+	236.214	6	6E,11Z-Hexadecadienal	C16H28O			NO	metabo_info.php?molid=46493
46494	237.21976	[M+H]+	236.214	6	8E,11Z-Hexadecadienal	C16H28O			NO	metabo_info.php?molid=46494
46495	237.21976	[M+H]+	236.214	6	9E,11Z-Hexadecadienal	C16H28O			NO	metabo_info.php?molid=46495
46507	237.21976	[M+H]+	236.214	6	7Z,11E-Hexadecadienal	C16H28O			NO	metabo_info.php?molid=46507

46508	237.21976	[M+H]+	236.214	6	9Z,11E-Hexadecadienal	C16H28O		NO	metabo_info.php?molid=46508
46509	237.21976	[M+H]+	236.214	6	10Z,12Z-Hexadecadienal	C16H28O		NO	metabo_info.php?molid=46509
46510	237.21976	[M+H]+	236.214	6	11Z,13Z-Hexadecadienal	C16H28O		NO	metabo_info.php?molid=46510
46511	237.21976	[M+H]+	236.214	6	7Z,11Z-Hexadecadienal	C16H28O		NO	metabo_info.php?molid=46511
46512	237.21976	[M+H]+	236.214	6	9Z,11Z-Hexadecadienal	C16H28O		NO	metabo_info.php?molid=46512
46514	237.21976	[M+H]+	236.214	6	11-Hexadecynal	C16H28O		NO	metabo_info.php?molid=46514
75368	237.21976	[M+H]+	236.214	6	6,11-hexadecadienal	C16H28O		NO	metabo_info.php?molid=75368
75369	237.21976	[M+H]+	236.214	6	10,12-hexadecadienal	C16H28O		NO	metabo_info.php?molid=75369
75370	237.21976	[M+H]+	236.214	6	11,13-hexadecadienal	C16H28O		NO	metabo_info.php?molid=75370
75389	237.21976	[M+H]+	236.214	6	10Z,12E-Hexadecadienal	C16H28O		NO	metabo_info.php?molid=75389
75390	237.21976	[M+H]+	236.214	6	11Z,13E-Hexadecadienal	C16H28O		NO	metabo_info.php?molid=75390
87971	237.21976	[M+H]+	236.214	6	3-(5,6,6-Trimethylbicyclo[2.2.1]hept-1-yl)cyclohexanol	C16H28O	3407-42-9	NO	metabo_info.php?molid=87971
91844	237.21976	[M+H]+	236.214	6	Ambronide	C16H28O	6790-58-5	NO	metabo_info.php?molid=91844
36498	237.21976	[M+Na]+	214.23	3	1-tetradecanol	C14H30O		YES	metabo_info.php?molid=36498
75264	237.21976	[M+Na]+	214.23	3	tetradecan1-ol	C14H30O		NO	metabo_info.php?molid=75264
96995	237.21976	[M+Na]+	214.23	3	3,9-Dimethyldodecan-6-ol	C14H30O		NO	metabo_info.php?molid=96995

97000	237.21976	[M+Na]+	214.23	3	4-methyltridecan-7-ol	C14H30O			NO	metabo_info.php?molid=97000
94573	242.09784	[M+Na]+	219.108	1	2-Hexylbenzothiazole	C13H17NS	65718-88-9		NO	metabo_info.php?molid=94573
241	242.09784	[M+Na]+	219.111	8	Pantothenic Acid	C9H17NO5	137-08-6	C00864	YES	metabo_info.php?molid=241
85228	305.15462	[M+H]+	304.145	6	Mepiprazole	C16H21ClN4			YES	metabo_info.php?molid=85228
37504	935.79897	[M+H]+	934.799	7	TG(18:2(9Z,12Z)/20:2(11Z,14Z)/20:2(11Z,14Z))[iso3]	C61H106O6			NO	metabo_info.php?molid=37504
37505	935.79897	[M+H]+	934.799	7	TG(18:3(9Z,12Z,15Z)/20:1(11Z)/20:2(11Z,14Z))[iso6]	C61H106O6			NO	metabo_info.php?molid=37505
37507	935.79897	[M+H]+	934.799	7	TG(18:0/20:3(8Z,11Z,14Z)/20:3(8Z,11Z,14Z))[iso3]	C61H106O6			NO	metabo_info.php?molid=37507
37508	935.79897	[M+H]+	934.799	7	TG(18:1(9Z)/20:2(11Z,14Z)/20:3(8Z,11Z,14Z))[iso6]	C61H106O6			NO	metabo_info.php?molid=37508
37509	935.79897	[M+H]+	934.799	7	TG(18:2(9Z,12Z)/20:1(11Z)/20:3(8Z,11Z,14Z))[iso6]	C61H106O6			NO	metabo_info.php?molid=37509
37510	935.79897	[M+H]+	934.799	7	TG(18:3(9Z,12Z,15Z)/20:0/20:3(8Z,11Z,14Z))[iso6]	C61H106O6			NO	metabo_info.php?molid=37510
37514	935.79897	[M+H]+	934.799	7	TG(18:0/20:2(11Z,14Z)/20:4(5Z,8Z,11Z,14Z))[iso6]	C61H106O6			NO	metabo_info.php?molid=37514
37515	935.79897	[M+H]+	934.799	7	TG(18:1(9Z)/20:1(11Z)/20:4(5Z,8Z,11Z,14Z))[iso6]	C61H106O6			NO	metabo_info.php?molid=37515
37516	935.79897	[M+H]+	934.799	7	TG(18:2(9Z,12Z)/20:0/20:4(5Z,8Z,11Z,14Z))[iso6]	C61H106O6			NO	metabo_info.php?molid=37516
37522	935.79897	[M+H]+	934.799	7	TG(18:0/20:1(11Z)/20:5(5Z,8Z,11Z,14Z,17Z))[iso6]	C61H106O6			NO	metabo_info.php?molid=37522

37523	935.79897	[M+H]+	934.799	7	TG(18:1(9Z)/20:0/20:5(5Z,8Z,11Z,14Z,17Z))[iso6]	C61H106O6	NO	metabo_info.php?molid=37523
37624	935.79897	[M+H]+	934.799	7	TG(16:0/20:3(8Z,11Z,14Z)/22:3(10Z,13Z,16Z))[iso6]	C61H106O6	NO	metabo_info.php?molid=37624
37625	935.79897	[M+H]+	934.799	7	TG(16:1(9Z)/20:2(11Z,14Z)/22:3(10Z,13Z,16Z))[iso6]	C61H106O6	NO	metabo_info.php?molid=37625
37629	935.79897	[M+H]+	934.799	7	TG(18:0/18:3(9Z,12Z,15Z)/22:3(10Z,13Z,16Z))[iso6]	C61H106O6	NO	metabo_info.php?molid=37629
37630	935.79897	[M+H]+	934.799	7	TG(18:1(9Z)/18:2(9Z,12Z)/22:3(10Z,13Z,16Z))[iso6]	C61H106O6	NO	metabo_info.php?molid=37630
37682	935.79897	[M+H]+	934.799	7	TG(16:1(9Z)/20:5(5Z,8Z,11Z,14Z,17Z)/22:0)[iso6]	C61H106O6	NO	metabo_info.php?molid=37682
37689	935.79897	[M+H]+	934.799	7	TG(18:3(9Z,12Z,15Z)/18:3(9Z,12Z,15Z)/22:0)[iso3]	C61H106O6	NO	metabo_info.php?molid=37689
37690	935.79897	[M+H]+	934.799	7	TG(16:0/20:5(5Z,8Z,11Z,14Z,17Z)/22:1(13Z))[iso6]	C61H106O6	NO	metabo_info.php?molid=37690
37691	935.79897	[M+H]+	934.799	7	TG(16:1(9Z)/20:4(5Z,8Z,11Z,14Z)/22:1(13Z))[iso6]	C61H106O6	NO	metabo_info.php?molid=37691
37697	935.79897	[M+H]+	934.799	7	TG(18:2(9Z,12Z)/18:3(9Z,12Z,15Z)/22:1(13Z))[iso6]	C61H106O6	NO	metabo_info.php?molid=37697
37713	935.79897	[M+H]+	934.799	7	TG(16:0/20:2(11Z,14Z)/22:4(7Z,10Z,13Z,16Z))[iso6]	C61H106O6	NO	metabo_info.php?molid=37713
37714	935.79897	[M+H]+	934.799	7	TG(16:1(9Z)/20:1(11Z)/22:4(7Z,10Z,13Z,16Z))[iso6]	C61H106O6	NO	metabo_info.php?molid=37714

37718	935.79897	[M+H]+	934.799	7	TG(18:0/18:2(9Z,12Z)/22:4(7Z,10Z,13Z,16Z))[iso6]	C61H106O6	NO	metabo_info.php?molid=37718
37719	935.79897	[M+H]+	934.799	7	TG(18:1(9Z)/18:1(9Z)/22:4(7Z,10Z,13Z,16Z))[iso3]	C61H106O6	NO	metabo_info.php?molid=37719
37720	935.79897	[M+H]+	934.799	7	TG(16:0/20:1(11Z)/22:5(7Z,10Z,13Z,16Z,19Z))[iso6]	C61H106O6	NO	metabo_info.php?molid=37720
37721	935.79897	[M+H]+	934.799	7	TG(16:1(9Z)/20:0/22:5(7Z,10Z,13Z,16Z,19Z))[iso6]	C61H106O6	NO	metabo_info.php?molid=37721
37725	935.79897	[M+H]+	934.799	7	TG(18:0/18:1(9Z)/22:5(7Z,10Z,13Z,16Z,19Z))[iso6]	C61H106O6	NO	metabo_info.php?molid=37725
37726	935.79897	[M+H]+	934.799	7	TG(16:0/20:0/22:6(4Z,7Z,10Z,13Z,16Z,19Z))[iso6]	C61H106O6	NO	metabo_info.php?molid=37726
37731	935.79897	[M+H]+	934.799	7	TG(18:0/18:0/22:6(4Z,7Z,10Z,13Z,16Z,19Z))[iso3]	C61H106O6	NO	metabo_info.php?molid=37731
37749	935.79897	[M+H]+	934.799	7	TG(17:1(9Z)/20:5(5Z,8Z,11Z,14Z,17Z)/21:0)[iso6]	C61H106O6	NO	metabo_info.php?molid=37749
37750	935.79897	[M+H]+	934.799	7	TG(17:2(9Z,12Z)/20:4(5Z,8Z,11Z,14Z)/21:0)[iso6]	C61H106O6	NO	metabo_info.php?molid=37750
37782	935.79897	[M+H]+	934.799	7	TG(16:0/20:4(5Z,8Z,11Z,14Z)/22:2(13Z,16Z))[iso6]	C61H106O6	NO	metabo_info.php?molid=37782
37783	935.79897	[M+H]+	934.799	7	TG(16:1(9Z)/20:3(8Z,11Z,14Z)/22:2(13Z,16Z))[iso6]	C61H106O6	NO	metabo_info.php?molid=37783
37788	935.79897	[M+H]+	934.799	7	TG(18:1(9Z)/18:3(9Z,12Z,15Z)/22:2(13Z,16Z))[iso6]	C61H106O6	NO	metabo_info.php?molid=37788
37789	935.79897	[M+H]+	934.799	7	TG(18:2(9Z,12Z)/18:2(9Z,12Z)/22:2(13Z,16Z))[iso3]	C61H106O6	NO	metabo_info.php?molid=37789

37794	935.79897	[M+H]+	934.799	7	TG(17:2(9Z,12Z)/19:0/22:4(7Z,10Z,13Z,16Z))[iso6]	C61H106O6	NO	metabo_info.php?molid=37794
37800	935.79897	[M+H]+	934.799	7	TG(17:1(9Z)/19:0/22:5(7Z,10Z,13Z,16Z,19Z))[iso6]	C61H106O6	NO	metabo_info.php?molid=37800
37806	935.79897	[M+H]+	934.799	7	TG(17:0/19:0/22:6(4Z,7Z,10Z,13Z,16Z,19Z))[iso6]	C61H106O6	NO	metabo_info.php?molid=37806
98668	935.79897	[M+H]+	934.799	7	TG(14:0/22:3(10Z,13Z,16Z)/22:3(10Z,13Z,16Z))[iso3]	C61H106O6	NO	metabo_info.php?molid=98668
98911	935.79897	[M+H]+	934.799	7	TG(18:3(6Z,9Z,12Z)/18:3(6Z,9Z,12Z)/22:0)[iso3]	C61H106O6	NO	metabo_info.php?molid=98911
98961	935.79897	[M+H]+	934.799	7	TG(18:4(6Z,9Z,12Z,15Z)/20:1(11Z)/20:1(11Z))[iso3]	C61H106O6	NO	metabo_info.php?molid=98961
98982	935.79897	[M+H]+	934.799	7	TG(19:1(9Z)/19:1(9Z)/20:4(5Z,8Z,11Z,14Z))[iso3]	C61H106O6	NO	metabo_info.php?molid=98982
100411	935.79897	[M+H]+	934.799	7	TG(14:0/22:0/22:6(4Z,7Z,10Z,13Z,16Z,19Z))[iso6]	C61H106O6	NO	metabo_info.php?molid=100411
100415	935.79897	[M+H]+	934.799	7	TG(14:0/22:1(11Z)/22:5(7Z,10Z,13Z,16Z,19Z))[iso6]	C61H106O6	NO	metabo_info.php?molid=100415
100418	935.79897	[M+H]+	934.799	7	TG(14:0/22:2(13Z,16Z)/22:4(7Z,10Z,13Z,16Z))[iso6]	C61H106O6	NO	metabo_info.php?molid=100418
100816	935.79897	[M+H]+	934.799	7	TG(14:1(9Z)/22:0/22:5(7Z,10Z,13Z,16Z,19Z))[iso6]	C61H106O6	NO	metabo_info.php?molid=100816
100820	935.79897	[M+H]+	934.799	7	TG(14:1(9Z)/22:1(11Z)/22:4(7Z,10Z,13Z,16Z))[iso6]	C61H106O6	NO	metabo_info.php?molid=100820
100823	935.79897	[M+H]+	934.799	7	TG(14:1(9Z)/22:2(13Z,16Z)/22:3(10Z,13Z,16Z))[iso6]	C61H106O6	NO	metabo_info.php?molid=100823

101182	935.79897	[M+H]+	934.799	7	TG(15:0/21:0/22:6(4Z,7Z,10Z,13Z,16Z,19Z))[iso6]	C61H106O6		NO	metabo_info.php?molid=101182
101532	935.79897	[M+H]+	934.799	7	TG(15:1(9Z)/21:0/22:5(7Z,10Z,13Z,16Z,19Z))[iso6]	C61H106O6		NO	metabo_info.php?molid=101532
101641	935.79897	[M+H]+	934.799	7	TG(16:0/20:5(5Z,8Z,11Z,14Z,17Z)/22:1(11Z))[iso6]	C61H106O6		NO	metabo_info.php?molid=101641
101731	935.79897	[M+H]+	934.799	7	TG(16:1(9Z)/20:4(5Z,8Z,11Z,14Z)/22:1(11Z))[iso6]	C61H106O6		NO	metabo_info.php?molid=101731
101812	935.79897	[M+H]+	934.799	7	TG(17:0/19:1(9Z)/22:5(7Z,10Z,13Z,16Z,19Z))[iso6]	C61H106O6		NO	metabo_info.php?molid=101812
101894	935.79897	[M+H]+	934.799	7	TG(17:1(9Z)/19:1(9Z)/22:4(7Z,10Z,13Z,16Z))[iso6]	C61H106O6		NO	metabo_info.php?molid=101894
101972	935.79897	[M+H]+	934.799	7	TG(17:2(9Z,12Z)/19:1(9Z)/22:3(10Z,13Z,16Z))[iso6]	C61H106O6		NO	metabo_info.php?molid=101972
102012	935.79897	[M+H]+	934.799	7	TG(18:0/18:3(6Z,9Z,12Z)/22:3(10Z,13Z,16Z))[iso6]	C61H106O6		NO	metabo_info.php?molid=102012
102030	935.79897	[M+H]+	934.799	7	TG(18:0/18:4(6Z,9Z,12Z,15Z)/22:2(13Z,16Z))[iso6]	C61H106O6		NO	metabo_info.php?molid=102030
102082	935.79897	[M+H]+	934.799	7	TG(18:1(9Z)/18:3(6Z,9Z,12Z)/22:2(13Z,16Z))[iso6]	C61H106O6		NO	metabo_info.php?molid=102082
102100	935.79897	[M+H]+	934.799	7	TG(18:1(9Z)/18:4(6Z,9Z,12Z,15Z)/22:1(11Z))[iso6]	C61H106O6		NO	metabo_info.php?molid=102100
102148	935.79897	[M+H]+	934.799	7	TG(18:2(9Z,12Z)/18:3(6Z,9Z,12Z)/22:1(11Z))[iso6]	C61H106O6		NO	metabo_info.php?molid=102148

102156	935.79897	[M+H]+	934.799	7	TG(18:2(9Z,12Z)/18:3(9Z,12Z,15Z)/22:1(11Z))[iso6]	C61H106O6	NO	metabo_info.php?molid=102156
102166	935.79897	[M+H]+	934.799	7	TG(18:2(9Z,12Z)/18:4(6Z,9Z,12Z,15Z)/22:0)[iso6]	C61H106O6	NO	metabo_info.php?molid=102166
102213	935.79897	[M+H]+	934.799	7	TG(18:3(6Z,9Z,12Z)/18:3(9Z,12Z,15Z)/22:0)[iso6]	C61H106O6	NO	metabo_info.php?molid=102213
102267	935.79897	[M+H]+	934.799	7	TG(18:3(6Z,9Z,12Z)/20:0/20:3(8Z,11Z,14Z))[iso6]	C61H106O6	NO	metabo_info.php?molid=102267
102278	935.79897	[M+H]+	934.799	7	TG(18:3(6Z,9Z,12Z)/20:1(11Z)/20:2(11Z,14Z))[iso6]	C61H106O6	NO	metabo_info.php?molid=102278
102432	935.79897	[M+H]+	934.799	7	TG(18:4(6Z,9Z,12Z,15Z)/20:0/20:2(11Z,14Z))[iso6]	C61H106O6	NO	metabo_info.php?molid=102432
102527	935.79897	[M+H]+	934.799	7	TG(19:0/19:1(9Z)/20:5(5Z,8Z,11Z,14Z,17Z))[iso6]	C61H106O6	NO	metabo_info.php?molid=102527
36976	935.79897	[M+Na]+	912.815	5	TG(18:0/18:3(9Z,12Z,15Z)/20:0)[iso6]	C59H108O6	NO	metabo_info.php?molid=36976
36977	935.79897	[M+Na]+	912.815	5	TG(18:1(9Z)/18:2(9Z,12Z)/20:0)[iso6]	C59H108O6	NO	metabo_info.php?molid=36977
36978	935.79897	[M+Na]+	912.815	5	TG(16:1(9Z)/20:1(11Z)/20:1(11Z))[iso3]	C59H108O6	NO	metabo_info.php?molid=36978
36982	935.79897	[M+Na]+	912.815	5	TG(18:0/18:2(9Z,12Z)/20:1(11Z))[iso6]	C59H108O6	NO	metabo_info.php?molid=36982
36983	935.79897	[M+Na]+	912.815	5	TG(18:1(9Z)/18:1(9Z)/20:1(11Z))[iso3]	C59H108O6	NO	metabo_info.php?molid=36983
36984	935.79897	[M+Na]+	912.815	5	TG(16:0/20:1(11Z)/20:2(11Z,14Z))[iso6]	C59H108O6	NO	metabo_info.php?molid=36984
36985	935.79897	[M+Na]+	912.815	5	TG(16:1(9Z)/20:0/20:2(11Z,14Z))[iso6]	C59H108O6	NO	metabo_info.php?molid=36985
36989	935.79897	[M+Na]+	912.815	5	TG(18:0/18:1(9Z)/20:2(11Z,14Z))[iso6]	C59H108O6	NO	metabo_info.php?molid=36989
36990	935.79897	[M+Na]+	912.815	5	TG(16:0/20:0/20:3(8Z,11Z,14Z))[iso6]	C59H108O6	NO	metabo_info.php?molid=36990

36995	935.79897	[M+Na]+	912.815	5	TG(18:0/18:0/20:3(8Z,11Z,14Z))[iso3]	C59H108O6	NO	metabo_info.php?molid=36995
37019	935.79897	[M+Na]+	912.815	5	TG(17:0/17:0/22:3(10Z,13Z,16Z))[iso3]	C59H108O6	NO	metabo_info.php?molid=37019
37035	935.79897	[M+Na]+	912.815	5	TG(17:2(9Z,12Z)/19:0/20:1(11Z))[iso6]	C59H108O6	NO	metabo_info.php?molid=37035
37041	935.79897	[M+Na]+	912.815	5	TG(17:1(9Z)/19:0/20:2(11Z,14Z))[iso6]	C59H108O6	NO	metabo_info.php?molid=37041
37047	935.79897	[M+Na]+	912.815	5	TG(17:0/19:0/20:3(8Z,11Z,14Z))[iso6]	C59H108O6	NO	metabo_info.php?molid=37047
37070	935.79897	[M+Na]+	912.815	5	TG(17:1(9Z)/17:2(9Z,12Z)/22:0)[iso6]	C59H108O6	NO	metabo_info.php?molid=37070
37073	935.79897	[M+Na]+	912.815	5	TG(17:0/17:2(9Z,12Z)/22:1(13Z))[iso6]	C59H108O6	NO	metabo_info.php?molid=37073
37074	935.79897	[M+Na]+	912.815	5	TG(17:1(9Z)/17:1(9Z)/22:1(13Z))[iso3]	C59H108O6	NO	metabo_info.php?molid=37074
37075	935.79897	[M+Na]+	912.815	5	TG(16:0/18:0/22:3(10Z,13Z,16Z))[iso6]	C59H108O6	NO	metabo_info.php?molid=37075
37126	935.79897	[M+Na]+	912.815	5	TG(16:0/18:3(9Z,12Z,15Z)/22:0)[iso6]	C59H108O6	NO	metabo_info.php?molid=37126
37127	935.79897	[M+Na]+	912.815	5	TG(16:1(9Z)/18:2(9Z,12Z)/22:0)[iso6]	C59H108O6	NO	metabo_info.php?molid=37127
37131	935.79897	[M+Na]+	912.815	5	TG(16:0/18:2(9Z,12Z)/22:1(13Z))[iso6]	C59H108O6	NO	metabo_info.php?molid=37131
37132	935.79897	[M+Na]+	912.815	5	TG(16:1(9Z)/18:1(9Z)/22:1(13Z))[iso6]	C59H108O6	NO	metabo_info.php?molid=37132
37141	935.79897	[M+Na]+	912.815	5	TG(17:0/17:1(9Z)/22:2(13Z,16Z))[iso6]	C59H108O6	NO	metabo_info.php?molid=37141
37149	935.79897	[M+Na]+	912.815	5	TG(18:3(9Z,12Z,15Z)/19:0/19:0)[iso3]	C59H108O6	NO	metabo_info.php?molid=37149
37185	935.79897	[M+Na]+	912.815	5	TG(17:0/18:3(9Z,12Z,15Z)/21:0)[iso6]	C59H108O6	NO	metabo_info.php?molid=37185
37186	935.79897	[M+Na]+	912.815	5	TG(17:1(9Z)/18:2(9Z,12Z)/21:0)[iso6]	C59H108O6	NO	metabo_info.php?molid=37186

37187	935.79897	[M+Na]+	912.815	5	TG(17:2(9Z,12Z)/18:1(9Z)/21:0)[iso6]	C59H108O6	NO	metabo_info.php?molid=37187
37203	935.79897	[M+Na]+	912.815	5	TG(16:0/18:1(9Z)/22:2(13Z,16Z))[iso6]	C59H108O6	NO	metabo_info.php?molid=37203
37204	935.79897	[M+Na]+	912.815	5	TG(16:1(9Z)/18:0/22:2(13Z,16Z))[iso6]	C59H108O6	NO	metabo_info.php?molid=37204
98865	935.79897	[M+Na]+	912.815	5	TG(17:1(9Z)/17:1(9Z)/22:1(11Z))[iso3]	C59H108O6	NO	metabo_info.php?molid=98865
98891	935.79897	[M+Na]+	912.815	5	TG(18:1(9Z)/19:1(9Z)/19:1(9Z))[iso3]	C59H108O6	NO	metabo_info.php?molid=98891
98920	935.79897	[M+Na]+	912.815	5	TG(18:3(6Z,9Z,12Z)/19:0/19:0)[iso3]	C59H108O6	NO	metabo_info.php?molid=98920
99508	935.79897	[M+Na]+	912.815	5	TG(12:0/22:0/22:3(10Z,13Z,16Z))[iso6]	C59H108O6	NO	metabo_info.php?molid=99508
99512	935.79897	[M+Na]+	912.815	5	TG(12:0/22:1(11Z)/22:2(13Z,16Z))[iso6]	C59H108O6	NO	metabo_info.php?molid=99512
99967	935.79897	[M+Na]+	912.815	5	TG(13:0/21:0/22:3(10Z,13Z,16Z))[iso6]	C59H108O6	NO	metabo_info.php?molid=99967
100345	935.79897	[M+Na]+	912.815	5	TG(14:0/20:0/22:3(10Z,13Z,16Z))[iso6]	C59H108O6	NO	metabo_info.php?molid=100345
100356	935.79897	[M+Na]+	912.815	5	TG(14:0/20:1(11Z)/22:2(13Z,16Z))[iso6]	C59H108O6	NO	metabo_info.php?molid=100356
100366	935.79897	[M+Na]+	912.815	5	TG(14:0/20:2(11Z,14Z)/22:1(11Z))[iso6]	C59H108O6	NO	metabo_info.php?molid=100366
100375	935.79897	[M+Na]+	912.815	5	TG(14:0/20:3(8Z,11Z,14Z)/22:0)[iso6]	C59H108O6	NO	metabo_info.php?molid=100375
100750	935.79897	[M+Na]+	912.815	5	TG(14:1(9Z)/20:0/22:2(13Z,16Z))[iso6]	C59H108O6	NO	metabo_info.php?molid=100750
100761	935.79897	[M+Na]+	912.815	5	TG(14:1(9Z)/20:1(11Z)/22:1(11Z))[iso6]	C59H108O6	NO	metabo_info.php?molid=100761
100771	935.79897	[M+Na]+	912.815	5	TG(14:1(9Z)/20:2(11Z,14Z)/22:0)[iso6]	C59H108O6	NO	metabo_info.php?molid=100771
101095	935.79897	[M+Na]+	912.815	5	TG(15:0/19:0/22:3(10Z,13Z,16Z))[iso6]	C59H108O6	NO	metabo_info.php?molid=101095

101108	935.79897	[M+Na]+	912.815	5	TG(15:0/19:1(9Z)/22:2(13Z,16Z))[iso6]	C59H108O6		NO	metabo_info.php?molid=101108
101151	935.79897	[M+Na]+	912.815	5	TG(15:0/20:3(8Z,11Z,14Z)/21:0)[iso6]	C59H108O6		NO	metabo_info.php?molid=101151
101445	935.79897	[M+Na]+	912.815	5	TG(15:1(9Z)/19:0/22:2(13Z,16Z))[iso6]	C59H108O6		NO	metabo_info.php?molid=101445
101458	935.79897	[M+Na]+	912.815	5	TG(15:1(9Z)/19:1(9Z)/22:1(11Z))[iso6]	C59H108O6		NO	metabo_info.php?molid=101458
101492	935.79897	[M+Na]+	912.815	5	TG(15:1(9Z)/20:2(11Z,14Z)/21:0)[iso6]	C59H108O6		NO	metabo_info.php?molid=101492
101582	935.79897	[M+Na]+	912.815	5	TG(16:0/18:2(9Z,12Z)/22:1(11Z))[iso6]	C59H108O6		NO	metabo_info.php?molid=101582
101594	935.79897	[M+Na]+	912.815	5	TG(16:0/18:3(6Z,9Z,12Z)/22:0)[iso6]	C59H108O6		NO	metabo_info.php?molid=101594
101669	935.79897	[M+Na]+	912.815	5	TG(16:1(9Z)/18:1(9Z)/22:1(11Z))[iso6]	C59H108O6		NO	metabo_info.php?molid=101669
101748	935.79897	[M+Na]+	912.815	5	TG(17:0/17:2(9Z,12Z)/22:1(11Z))[iso6]	C59H108O6		NO	metabo_info.php?molid=101748
101771	935.79897	[M+Na]+	912.815	5	TG(17:0/18:3(6Z,9Z,12Z)/21:0)[iso6]	C59H108O6		NO	metabo_info.php?molid=101771
101802	935.79897	[M+Na]+	912.815	5	TG(17:0/19:1(9Z)/20:2(11Z,14Z))[iso6]	C59H108O6		NO	metabo_info.php?molid=101802
101884	935.79897	[M+Na]+	912.815	5	TG(17:1(9Z)/19:1(9Z)/20:1(11Z))[iso6]	C59H108O6		NO	metabo_info.php?molid=101884
101962	935.79897	[M+Na]+	912.815	5	TG(17:2(9Z,12Z)/19:1(9Z)/20:0)[iso6]	C59H108O6		NO	metabo_info.php?molid=101962
102002	935.79897	[M+Na]+	912.815	5	TG(18:0/18:3(6Z,9Z,12Z)/20:0)[iso6]	C59H108O6		NO	metabo_info.php?molid=102002
102173	935.79897	[M+Na]+	912.815	5	TG(18:2(9Z,12Z)/19:0/19:1(9Z))[iso6]	C59H108O6		NO	metabo_info.php?molid=102173
63114	373.21779	[M+H]+	372.211	1	13,14-dihydro-16,16-difluoro PGJ2	C20H30F2O4	NA	YES	metabo_info.php?molid=63114

70392	373.21779	[M+Na]+	350.226	7	5alpha-Androstane-2beta-fluoro-17beta-ol-3-one acetate	C21H31FO3	C14889	NO	metabo_info.php?molid=70392
70564	373.21779	[M+Na]+	350.226	7	6beta-Fluoro-5alpha-hydroxypregnane-3,20-dione	C21H31FO3	C15071	NO	metabo_info.php?molid=70564
70605	373.21779	[M+Na]+	350.226	7	9-Fluoro-11beta,17beta-dihydroxy-2,17-dimethylandrost-4-en-3-one	C21H31FO3	C15113	NO	metabo_info.php?molid=70605
70656	373.21779	[M+Na]+	350.226	7	5alpha-Androstane-2alpha-fluoro-17beta-ol-3-one acetate	C21H31FO3	C15166	NO	metabo_info.php?molid=70656
